# Mechanistic study of nucleophilic fluorination for the synthesis of fluorine-18 labeled fluoroform with high molar activity from *N*-difluoromethyltriazolium triflate†

**DOI:** 10.1039/d0ra09827b

**Published:** 2021-02-03

**Authors:** Jin Young Chai, Hyojin Cha, Sung-Sik Lee, Young-Ho Oh, Sungyul Lee, Dae Yoon Chi

**Affiliations:** Department of Chemistry, Sogang University 35 Baekbeomro Mapogu Seoul 04107 Korea dychi@sogang.ac.kr; Department of Applied Chemistry, Kyung Hee University 1732 Deogyeong-daero, Giheung-gu Yongin-si Gyeonggi-do 17104 Korea sylee@khu.ac.kr

## Abstract

The synthesis of fluorine-18 labeled fluoroform with high molar activity has grown in importance for the development of fluorine-18 labeled aryl-CF_3_ radiopharmaceuticals that are useful as diagnostic radiotracers for the powerful technique of positron emission tomography (PET). We designed a strategy of synthesizing fluorine-18 labeled fluoroform from *N*1-difluoromethyl-*N*3-methyltriazolium triflate (1) *via* S_N_2 fluorination without stable fluorine isotope scrambling. Fluoroform was generated at rt in 10 min by fluorination of the triazolium precursor with TBAF (6 equiv.). We propose three routes (a), (b), and (c) for this fluorination. Quantum chemical calculations have been carried out to elucidate the mechanism of experimentally observed nucleophilic attack of fluoride at difluoromethyl group *via* route (a), not *N*3-methyl *via* route (b). ^1^H and ^19^F NMR studies using deuterium source have been performed to examine the competition between S_N_2 fluorination (route (a)) and the formation of difluorocarbene (route (c)). The observed superiority of S_N_2 pathway to formation of difluorocarbene in the reaction of the precursor using CsF in (CD_3_CN/(CD_3_)_3_COD (17.8 : 1)) gives the possibility of preparing the fluorine-18 labeled fluoroform in high molar activity.

## Abstract 2

Route a: desired S_N_2 reaction of fluoride to form fluoroform with high molar activity; route b: side reaction to form methyl fluoride; route c: side reaction to form difluorocarbene to give fluoroform with lower molar activity.

## Graphical abstract

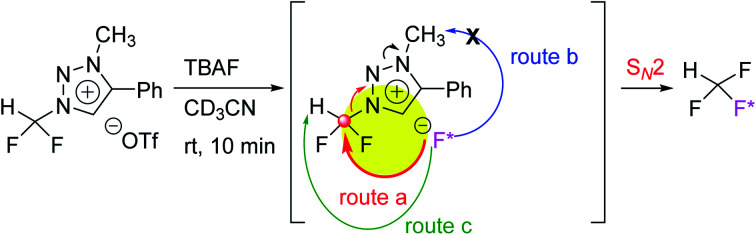

## Introduction

Aryl-CF_3_ is a moiety widely found in the structure of drugs.^1^ Among the fluorine containing drugs approved by the Food and Drug Administration during 1950–2014, twenty-one percent contained aryl-CF_3_ and 1% contained alkyl-CF_3_.^2^ As the positron emission tomography (PET) gains more and more importance as a powerful technique for probing the distribution of injected radiopharmaceuticals labeled with positron emitter radionuclides in the human body,^3^ the synthesis of radiopharmaceuticals becomes a critical task to apply this robust method to a variety of medical problems. Since fluorine-18 has relatively low energy (638 keV) and the approximately 110 min of half-life, the radioisotope fluorine-18 is considered to be the best positron emitter.^4^ Synthesis of [^18^F]aryl-CF_3_ radiopharmaceuticals^5,6^ makes it possible to easily measure the biodistribution of drugs with PET, consequently shortening the drug development period. It is especially important that introduction of fluorine-18 into drugs should proceed under mild conditions, *i.e.*, at low temperatures, and be carried out fast due to the short half-life of fluorine-18 radionuclide.^7^

**Scheme 1:**
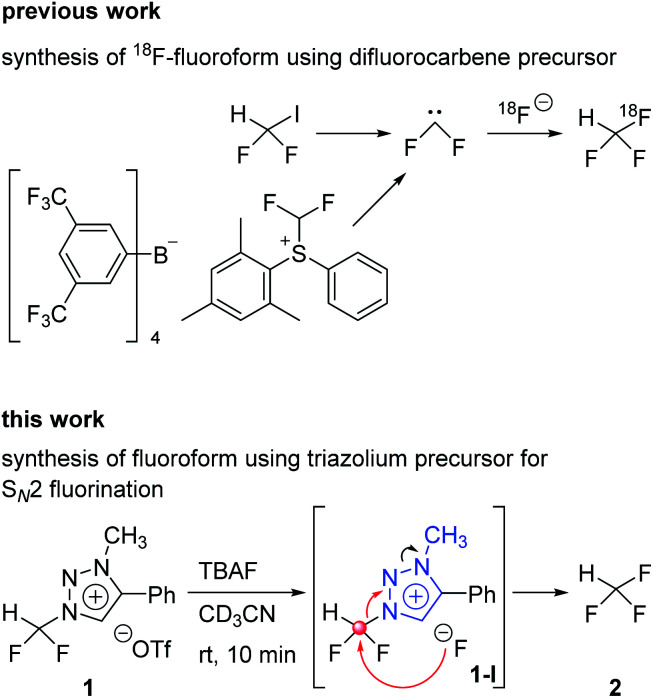
Previous work and this work.

A recent method to prepare aryl-CF_3_ is to use trifluoromethyl anion generated from fluoroform for trifluoromethylation reaction.^8^ The aryl-[^18^F]CF_3_ is prepared through the formation of [^18^F]CuCF_3_ intermediate from [^18^F]fluoroform. Among the [^18^F]fluoroform synthesis methods, that using difluoroiodomethane or difluoromethylsulfonium salt as a difluorocarbene precursor has been studied (Scheme 1).^9,10^ However, due to the isotopic dilution phenomenon,^10^ the difluorocarbene pathway gave a low molar activity (*A*_m_) of [^18^F]fluoroform (*A*_m_ = 1–32 GBq μmol^−1^).^9^ A method of synthesizing [^18^F]fluoroform with improved *A*_m_ has been recently reported, which was achieved by limiting the difluorocarbene formation using [^18^F]fluoromethane and CoF_3_ (*A*_m_ = maximum 163 GBq μmol^−1^ and average of 38 ± 35 GBq μmol^−1^ (*n* = 20)).^11^ In addition, a study of producing [^18^F]fluoroform with increased *A*_m_ has been reported, based on the strategy using [^18^F]triflyl fluoride under less basic condition to reduce the decomposition of difluoroiodomethane (*A*_m_ = 97 ± 20 GBq μmol^−1^ (*n* = 3)).^12^

In 2013, we reported that the triazolium group of 1,2,3-triazolium triflate functioned as a very good nucleofuge for displacement by the fluoride ion.^13^ The experimental conditions of this method were so mild that it could be used for the preparation of PET radiopharmaceuticals bearing *O*-[^18^F]fluoromethyl groups. As mentioned earlier, the synthesis of [^18^F]fluoroform with high molar activity is very critical. We have designed a strategy of synthesizing [^18^F]fluoroform from *N*-difluoromethyltriazolium triflate 1, utilizing the fact that 1,2,3-triazolium group may act as an excellent leaving group in fluorination reactions (Scheme 1). If this reaction proceeds *via* S_N_2 mechanism without stable fluorine isotope scrambling, the synthesis of [^18^F]fluoroform with high *A*_m_ would be possible. Here, we consider two plausible mechanisms for the synthesis of fluoroform from triazolium precursor 1 – either *via* S_N_2 fluorination or *via* the formation of difluorocarbene. We investigated: (1) the stability of triazolium precursor 1 with respect to temperature and solvents, (2) optimal fluorination conditions, (3) the nucleophilic selectivity toward *N*1-difluoromethyl and *N*3-methyl groups of triazolium precursor, (4) the effect of hydrogen bonding with C5-hydrogen and fluoride in the fluorination of triazolium precursor, (5) the mechanism (pre-reaction complex, transition state, post-reaction complex) of the reactions along with the energetics of triazolium precursor by quantum chemical calculations, and (6) the confirmation of the S_N_2 reaction mechanism by NMR study of fluorination.

## Results and discussion

### Stability of triazolium precursor 1

To confirm the stability of triazolium precursor 1, experiments were performed by changing solvents and temperature (Table 1). Decomposition of precursor 1 was not observed when 1 was heated at 80 °C for 60 h in acetonitrile. Precursor 1 was very stable in acetonitrile at high temperature (entry 1). DMF (bp = 153 °C) was used to confirm the stability of 1 at higher temperature in the following attempts: when 1 was heated at 110 °C for 36 h in DMF, demethylation occurred, resulting in 3 (entry 3). Following the reaction at 110 °C, the temperature was raised to 150 °C. Then the demethylation occurred in a total yield of 32% when 1 was heated for additional 36 h (entry 5). The precursor 1 decomposed very slowly in DMF at high temperature. Compound 3 was confirmed by observing the signals of the CF_2_H and C5 proton in ^1^H NMR (see ESI†).

**Table 1:** Investigation of stability of precursor 1^a^

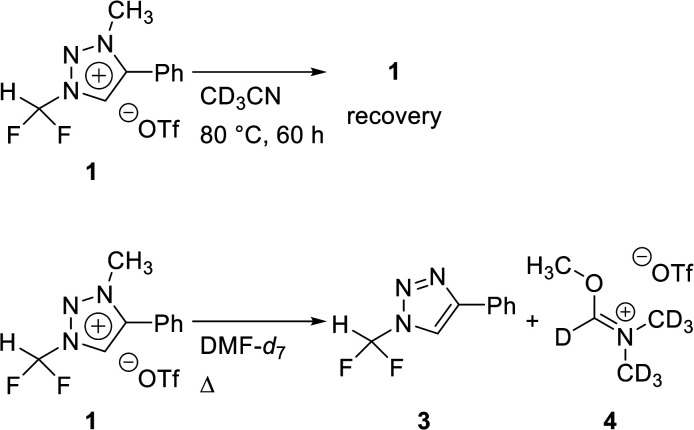					
Entry	Solvent	Temp. (°C)	Time (h)	Yield^b^ (%)	
				1	3
1	CD_3_CN	80	60	100	0
2	DMF-*d*_7_	110	1	100	0
3	DMF-*d*_7_	110	36	94	6
4	DMF-*d*_7_	110 to 150	48	81	19
5	DMF-*d*_7_	110 to 150	72	68	32

a All reactions were carried out on a 0.084 mmol reaction scale of triazolium precursor 1 in 0.75 mL of solvent in a sealed NMR tube.

b ^1^H NMR yields.

### Optimization of fluorination from triazolium precursor 1

Difluoroiodomethane is a gas, whereas triazolium precursor 1 is a bench-stable solid. Thus, triazolium precursor 1 is more convenient to weigh and to quantify the experimental observations. We searched efficiently the optimal condition of the synthesis of fluoroform. The reaction conditions of fluorination were investigated by using CsF or TBAF (Table 2). When CsF (2 equiv.) was used, the reaction proceeded 70% after 1 h and it was complete after 8 h (entries 1 and 4). When CsF was increased to 6 equiv., the reaction proceeded 77% after 1 h and it was complete after 5 h (entries 5 and 6). When TBAF (6 equiv.) was used instead of CsF, fluoroform 2 was generated faster. After 10 min at 80 °C, the reaction was complete (entry 7). The ^1^H NMR signal of *N*3-methyl group of 1 decreased and the signal of methyl group of 5 as a leaving group increased (see ESI†). A quartet from fluoroform 2 was observed in ^1^H NMR. Fluorination using TBAF was faster, indicating that reactivity of F^−^ in TBAF was higher than that in CsF. When reaction temperature was lowered to rt, the reaction proceeded 86% after 10 min (entry 8). The reaction rate decreased when TBAF was reduced to 2 equiv. (entries 9 and 10). When DMF-*d*_7_ was used as solvent, the reaction was complete in 10 min at 80 °C (entry 11).

**Table 2:** Optimization of reaction conditions using precursor 1^a^

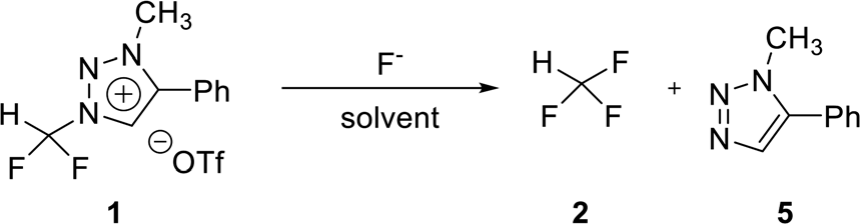					
Entry	F^−^ source (equiv.)	Solvent	Temp. (°C)	Time	Yield 5^b^ (%)
1	CsF (2.0)	CD_3_CN	80	1 h	70
2	CsF (2.0)	CD_3_CN	80	2 h	81
3	CsF (2.0)	CD_3_CN	80	4 h	93
4	CsF (2.0)	CD_3_CN	80	8 h	100
5	CsF (6.0)	CD_3_CN	80	1 h	77
6	CsF (6.0)	CD_3_CN	80	5 h	100
7	TBAF (6.0)	CD_3_CN	80	10 min	100
8	TBAF (6.0)	CD_3_CN	rt	10 min	86
9	TBAF (2.0)	CD_3_CN	rt	10 min	51
10	TBAF (2.0)	CD_3_CN	rt	1 h	64
11	TBAF (2.0)	DMF-*d*_7_	80	10 min	100

a All reactions were carried out on a 0.084 mmol reaction scale of triazolium precursor 1 in 0.75 mL of solvent in a sealed NMR tube.

b ^1^H NMR yields.

### Nucleophilic selectivity toward triazolium precursor 1

We propose three competing routes (a), (b), and (c). Route (a) is the desired route, in which the fluoride attacks the carbon of *N*1-difluoromethyl *via* S_N_2 pathway, producing fluoroform. In route (b), which is a side route, F^−^ attacks the carbon of *N*3-methyl. The last route (c) is an undesired route, in which the nucleophile F^−^ attacks the hydrogen atom of *N*1-difluoromethyl, consequently giving fluoroform *via* the formation of difluorocarbene.

Triazolium precursor 1 was also allowed to react with 1-phenylpiperazine to study the nucleophilic selectivity toward 1 (Table 3). If the nucleophile 1-phenylpiperazine attacks the difluoromethyl group, 6 and methyltriazole 5 will be formed through route (a). On the other hand, if the nucleophile 1-phenylpiperazine attacks *N*3-methyl, difluoromethyltriazole 3 will be formed through route (b). When precursor 1 reacted with 1-phenylpiperazine as a nucleophile at 80 °C, demethylation of *N*3-methyl group occurred predominantly than route (a).

**Table 3:** Reactions of triazolium precursor 1 with 1-phenylpiperazine^a^

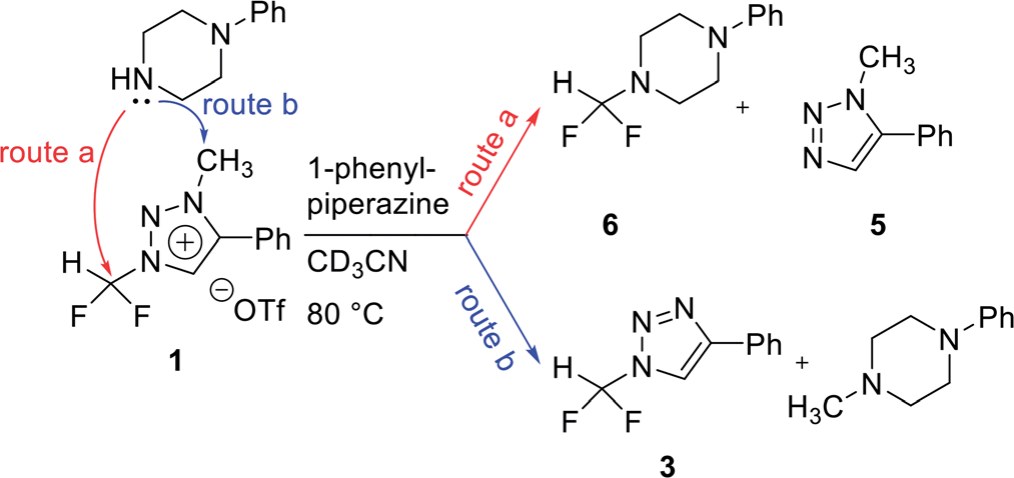				
Entry	Time (h)	Yield^b^ (%)		
		1	3	5
1	5.5	68	25	7
2	10	60	32	8
3	24	43	47	10
4	48	28	59	13

a All reactions were carried out on a 0.084 mmol reaction scale of triazolium precursor 1 in 0.75 mL of CD_3_CN in a sealed NMR tube. 1-Phenylpiperazine (3 equiv.) was used as a nucleophile.

b ^1^H NMR yields.

When F^−^ was used as a nucleophile (Table 2), only route (a) proceeded, producing fluoroform (2). On the other hand, when 1-phenylpiperazine (3 equiv.) was used as a nucleophile, route (b) was followed, giving 3 as the main product (Table 3). There are two reasons why demethylation of *N*3-methyl group occurred. First, the difluoromethyl group has a steric hindrance compared to methyl group. The difluoromethyl group is difficult to react with 1-phenylpiperazine *via* S_N_2 due to the steric hindrance of fluorine, which is slightly larger in volume than hydrogen. However, as *N*3-methyl has only hydrogen atoms having smaller volume than a fluorine, S_N_2 reaction can occur well. Fluoride produced fluoroform well because the fluoride received less steric hindrance toward difluoromethyl group than 1-phenylpiperazine did. Since fluoride is a nucleophile of smaller size than 1-phenylpiperazine, it attacks the difluoromethyl group, producing fluoroform. On the contrary, 1-phenylpiperazine attacked *N*3-methyl with less steric hindrance, and demethylation occurred. Second, there is a difference in the efficiency of exchange of the nucleophile with triflate anion. It may be speculated that fluoride was able to selectively produce fluoroform because of the increased access to difluoromethyl, after the fluoride anion exchanged with triflate anion of triazolium precursor. In the case of 1-phenylpiperazine, it is also presumed that demethylation occurred in relatively accessible *N*3-methyl, since there was no anion exchange between the triflate anion and the nucleophile.

### Reactions of benzotriazolium precursor 7

To confirm the effects of hydrogen bonding of C5 proton of triazolium precursor 1 with fluoride anion, the reaction of benzotriazolium precursor 7 with no C5 proton of triazolium precursor 1 was investigated (Table 4). When CsF reacted with benzotriazolium precursor 7 at 80 °C for 24 h, 47% of starting material 7 remained, confirming that the reactivity of 7 was lower than that of triazolium precursor 1 (entry 1).

**Table 4:** Nucleophilic reactions of benzotriazolium precursor 7^a^

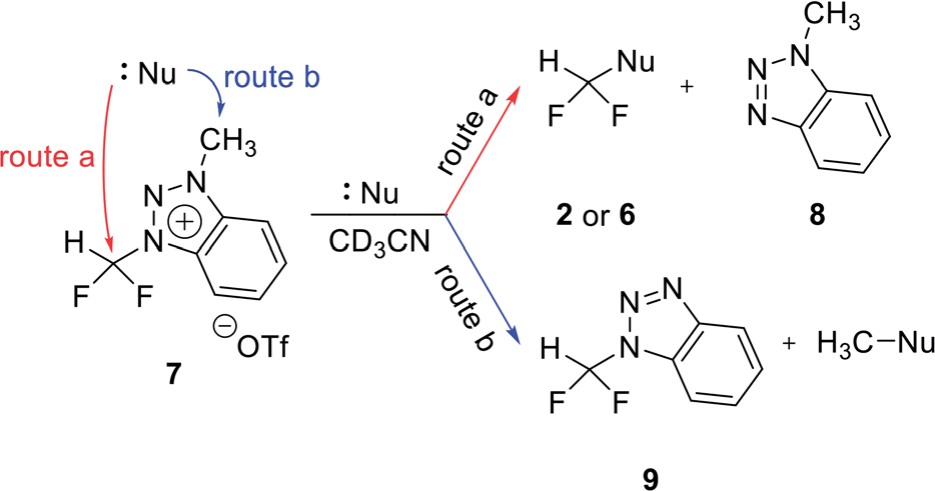						
Entry	Nucleophile (equiv.)	Temp. (°C)	Time	Yield^b^ (%)		
				7	9	8
1	CsF (2.0)	80	24 h	47	27	26
2	TBAF (2.0)	rt	10 min	46	1	53
3	TBAF (2.0)	rt	3.5 h	28	2	70
4	TBAF (2.0)	rt	27.5 h	0	3	97
5	1-Phenylpiperazine (3.0)	80	5.5 h	78	19	3
6	1-Phenylpiperazine (3.0)	80	10 h	65	31	4
7	1-Phenylpiperazine (3.0)	80	24 h	45	51	4
8	1-Phenylpiperazine (3.0)	80	48 h	27	68	5

a All reactions were carried out on a 0.090 mmol reaction scale of benzotriazolium precursor 7 in 0.75 mL of CD_3_CN in a sealed NMR tube.

b ^1^H NMR yields.

The positive charge of the benzotriazolium leaving group seems to be delocalized, because it can be distributed over both triazole and benzene rings. Thus, the benzotriazolium leaving group is difficult to fall off than triazolium. In addition, fluoroform (2) and methylbenzotriazole 8 were generated by fluorination reaction *via* route (a) as shown in Table 4. Difluoromethylbenzotriazole 9 was generated by demethylation through route (b). Unlike triazolium precursor 1, demethylation occurred in the reaction with CsF, because fluoride is less likely to produce fluoroform due to the lack of hydrogen bond involving C5 proton of triazolium salt 1. It is known that in nucleophilic fluorination, *tert*-alcohols exhibit the effects of weakening the ionic bond of CsF by the formation of hydrogen bonding with fluoride of CsF, thus making fluoride a good nucleophile by solvating it.^14^ The p*K*_a_ of C5 proton of *N*3-alkylated 1,2,3-triazolium salt is about 24,^15^ lower in acidity to *tert*-alcohol (p*K*_a_ = 19). Therefore, the C5 proton of triazolium precursor 1 seems to increase the nucleophilicity of fluoride through hydrogen bonding.

When TBAF reacted with 7 at rt, the formation of fluoroform proceeded in 53% of yield *via* route (a) after 10 min, and demethylation occurred only 1% (entry 2). We suggest that the observed low demethylation indicates that the fluoride of TBAF is more nucleophilic than CsF, so the fluoroform production reaction occurs quickly and predominantly. In reactions of 7 with 1-phenylpiperazine, demethylation *via* route (b) occurred more readily than fluoroform formation (route (a)) (entries 5–8), similar to the case of triazolium precursor 1.

### Quantum chemical analysis of reaction mechanism

**Fig. 1:**
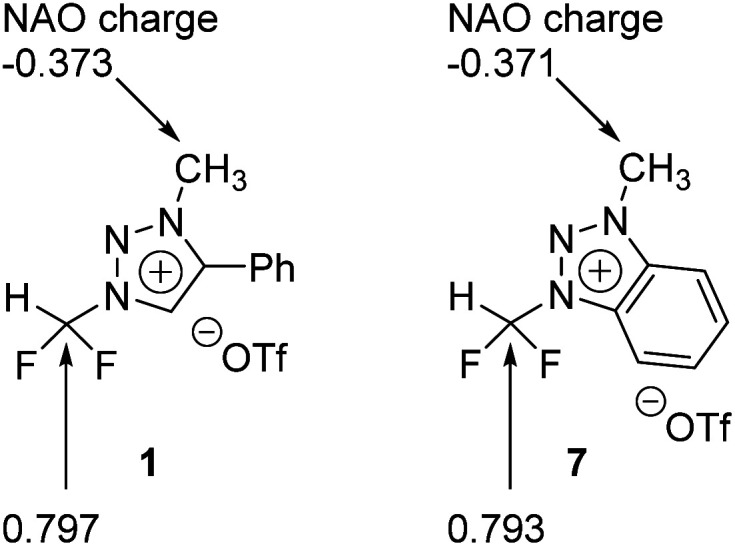
Natural atomic orbital analysis of precursors 1 and 7.

We carried out Natural Atomic Orbital (NAO)^16^ analysis, finding that the carbon atom of CF_2_H has partial positive charge (∼+0.79) because of the strong electron-withdrawing inductive effects of two Fs, whereas the electric charge of the methyl C atom is negative (∼−0.37) because of electron-donating property of the methyl group (Fig. 1). This may well explain the fact that fluoride reacts selectively to CF_2_H of triazolium precursor 1 when CsF or TBAF is used as a nucleophile. However, it seems to be difficult to explain why the S_N_2 reaction also occurred toward the methyl side when 1-phenylpiperazine was used as a nucleophile.

**Fig. 2:**
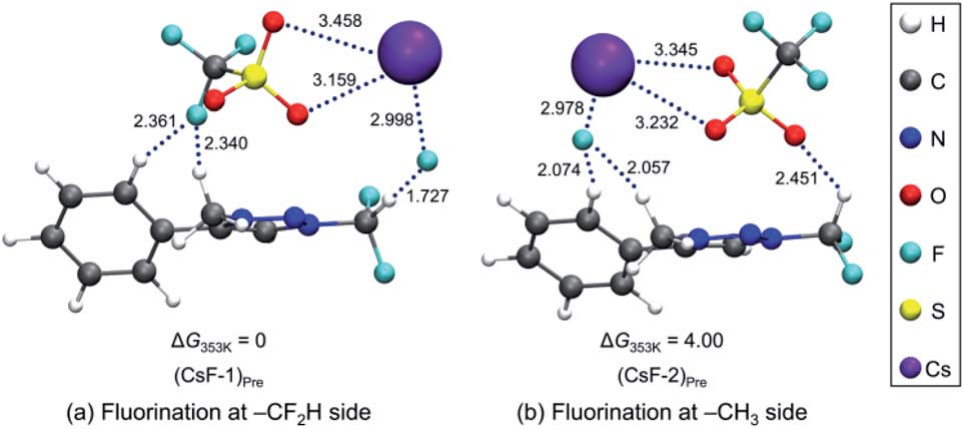
Pre-reaction complexes of reactions using CsF (a) at –CF_2_H and (b) –CH_3_ side.

To elucidate the mechanism of these experimentally observed reactions using triazolium precursor 1, we carried out systematic quantum chemical calculations using the M06-2X^17^ method with 6-311+G** basis set for H, C, N, O, S atoms, as implemented in Gaussian 09.^18^ The LANL2DZ pseudo-potential^19^ and its corresponding basis set is used for Cs atom. We found that all reactions proceeded by S_N_2 mechanism *via* single transition state, as it was the case for nucleophilic fluorination using CsF in various solvent/promoters. Fig. 2 depicts the pre-reaction complexes of fluorination using CsF at –CF_2_H and at –CH_3_ side. In (CsF-1)_Pre_, in which the nucleophile F^−^ attacks the –CF_2_H side, F^−^ forms strong hydrogen bond with H of the –CF_2_H group with a distance of 1.727 Å. Cs^+^ lies close to F and two O atoms of –OTf above the triazolium ring. In (CsF-2)_Pre_, F^−^ approaches the carbon center at –CH_3_. The local environment of Cs^+^ with respect to F and two O atoms is similar to (CsF-1)_Pre_, with the distances of 2.978, 3.232, 3.345 Å, respectively. The distance between F^−^ and methyl H is 2.057 Å, which is somewhat larger than that between F^−^ and H of CF_2_H of (CsF-1)_Pre_ because of the less polarized –CH_3_ in the absence of the inductive effects of the F atoms. Consequently, F^−^ interacts slightly more strongly with the Cs atom (*R*_Cs⋯F_ = 2.978 Å) as compared with (CsF-1)_Pre_ (*R*_Cs⋯F_ = 2.998 Å).

Our calculations show that the Gibbs free energy of the pre-reaction complex for fluorination at –CF_2_H is much lower (by 4.0 kcal mol^−1^) than that for the reaction at –CH_3_ side, indicating that the former complex is much more stable. This seems to be the result of much stronger electrostatic attraction between the nucleophile and the partially positive charged (+0.797) C center in –CF_2_H than that in –CH_3_ whose carbon center is partially *negative* (−0.373). The relative thermodynamic stability of (CsF-1)_Pre_ with respect to (CsF-2)_Pre_ (Δ*G* = 4.0 kcal mol^−1^) indicates that the population of pre-reaction (CsF-1)_Pre_ at thermal equilibrium (that is, according to the Boltzmann distribution *P*_1_/*P*_2_ = exp(−(*G*_1_ − *G*_2_)/*RT*)) would be much larger (by a factor of ∼250 : 1, suggesting that the relative population of (CsF-2)_Pre_ is essentially vanishing), well accounting for the experimentally observed exclusive formation of compound 5 (100% in 8 h, Table 2, entry 4) resulting from the reaction at –CF_2_H side.

**Fig. 3:**
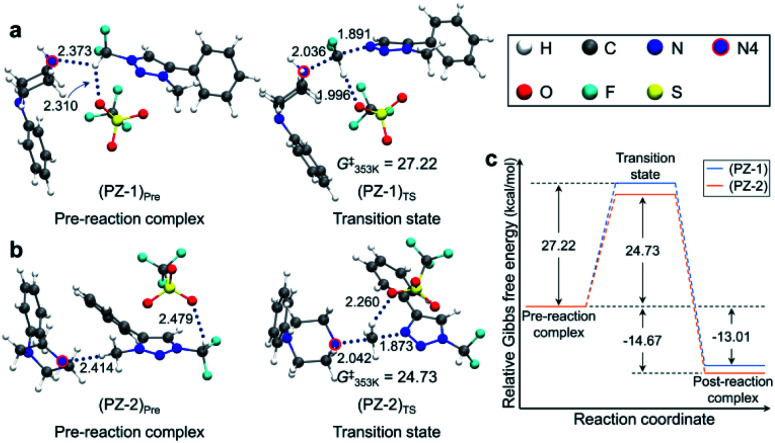
Pre-reaction complexes and transition states of reactions using 1-phenylpiperazine (a) at –CF_2_H and (b) –CH_3_ side and (c) energetics of reaction.

Pre-reaction complexes and transition states for the [triazolium][piperazine] system are shown in Fig. 3 along with the energetics of reaction. In (PZ-1)_Pre_, the pre-reaction complex for reaction at –CF_2_H site, due to the inductive effect of ‘CF_2_’, the highly polarized C–H forms hydrogen bond with lone pair electrons of N4 nitrogen of 1-phenylpiperazine and with an O atom of –OTf. In (PZ-2)_Pre_, the nucleophile (1-phenylpiperazine) reacts at the –CH_3_ group. The piperazine moiety is on the opposite side of –OTf of the phenyl ring of triazolium molecule. The distance between N4 and H of CH_3_ group is 2.414 Å which value is larger than that of (PZ-1)_Pre_ (*R*_N⋯H_ = 2.373 Å). In both structures the –OTf group lies above the triazolium, forming hydrogen bond with the –CF_2_H group. The Gibbs free energies at 353 K for these two structures are very similar.

Fig. 3 also presents the transition states. In (PZ-1)_TS_, The N4 lone pair electrons point toward C of –CF_2_H group due to the hydrogen bond. The distance *R*_H⋯O_ between –OTf and C–H of the –CF_2_H group decreases from 2.310 Å in the pre-reaction complex (PZ-1)_Pre_ to 1.996 Å, indicating that the hydrogen bond is strengthened in the transition state. The O atoms of –OTf are near the phenyl hydrogens of the triazolium molecule and of piperazine moiety. In (PZ-2)_TS_, however, the –OTf group is above the triazolium ring and far away from the piperazine moiety. The N4 of 1-phenylpiperazine in (PZ-1)_TS_ is slightly closer to the C of CF_2_H group with the distance of 2.036 Å in comparison with the corresponding distance (2.042 Å) in (PZ-2)_TS_.

The activation barrier of reaction at –CF_2_H and –CH_3_ side is 27.2 and 24.7 kcal mol^−1^, respectively (Fig. 3c). Lower (by 2.5 kcal mol^−1^) activation barrier at –CH_3_ than at –CF_2_H side indicates that the rate of the former reaction (at –CH_3_) is larger than that at –CF_2_H side, in excellent agreement with the experimental observations of much larger yield (59%) of reaction using 1-phenylpiperazine at –CH_3_ compared with that (13%) at –CF_2_H side after 48 h at 80 °C (Table 3, entry 4). This observation is especially conspicuous, because the Natural Atomic Orbital (NAO)^16^ charge of the C atom on which the nucleophile (1-phenylpiperazine) carries out the nucleophilic attack is −0.373 (+0.797) for reactions at –CH_3_ (–CF_2_H) group in the pre-reaction complex (PZ-1)_Pre_ ((PZ-2)_Pre_). Common sense would predict that 1-phenylpiperazine would prefer –CF_2_H to –CH_3_ side, whose carbon atom even exhibits a partial *negative* charge. The origin of this stark contrast of reaction yields using 1-phenylpiperazine compared with those using CsF, in which no reaction at –CH_3_ side is observed, is highly intriguing, and necessitates a systematic mechanistic elucidation.

Because the two pre-reaction complexes are essentially degenerate, this difference in activation barriers at –CF_2_H and –CH_3_ side seems to originate from the structures of the transition states. Most notable difference in (PZ-1)_TS_ and (PZ-2)_TS_ shown in Fig. 3 is the location of the –OTf group with respect to the 1-phenylpiperazine and triazolium moieties: in (PZ-1)_TS_ the –OTf group lies between these two latter functional groups, and the electronegative O atom in –OTf exerts attractive force on the piperazine unit, pulling N4 toward –CF_2_H. In (PZ-2)_TS_, no such effects of –OTf exists, because it lies above triazolium far from the piperazine molecule in (PZ-2)_TS_. This difference can be seen from the distance *R*_N–C_ in (PZ-1)_TS_ (2.036 Å) and in (PZ-2)_TS_ (2.042 Å). As compared with (PZ-2)_TS_, the better proximity of N4 to the carbon center in –CF_2_H in the transition state (PZ-1)_TS_ against the electrostatic repulsion by the electronegative F atoms in –CF_2_H renders the activation barrier for reaction at the –CF_2_H side larger than that at –CH_3_ side.

### Competition between S_N_2 and difluorocarbene pathways

**Scheme 2:**
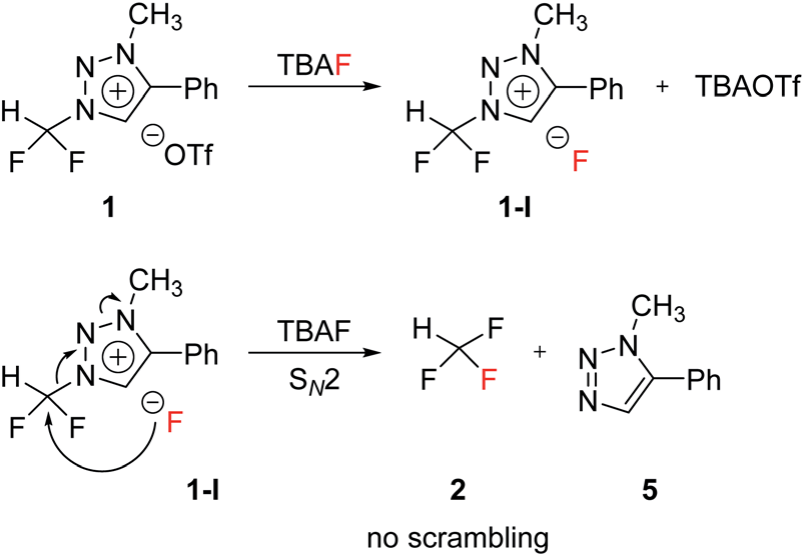
Proposed mechanism.

The mechanism of fluorination of 1 was as proposed in Scheme 2. Triflate ion was substituted with F^−^ in TBAF. Because of high nucleophilicity of F^−^ substituted on the triazolium ring, fluorination could proceed *via* S_N_2 pathway. We expect that S_N_2 fluorination will generate [^18^F]fluoroform with higher *A*_m_.

When triazolium precursor 1 reacted with CsF or TBAF, fluoroform (2) was formed. There are two possible pathways to form fluoroform: (1) fluoride attacks the carbon of difluoromethyl *via* S_N_2, and/or (2) fluoride deprotonates proton of difluoromethyl, leading to formation of difluorocarbene. To examine the competition between two pathways, ^1^H and ^19^F NMR studies using either CD_3_OD, CD_3_CN/CD_3_OD or CD_3_CN/(CD_3_)_3_COD were performed (Table 5), because nucleophilic fluorination goes well in non-polar protic solvent (*tert*-alcohol) rather than polar protic solvent (methanol).^14^ If reaction proceeds *via* S_N_2 fashion (route (a)), the proton of difluoromethyl will not be attacked and thus CF_3_H will be formed. In contrast, if difluorocarbene is produced *via* route (c), 14 will react with deuterium source and thus CF_3_D will be generated. Also, if 14 reacts with H_2_O in the reaction mixture, CF_3_H will be formed. Thus, the total yield of fluoroform (2) is the sum of the yield of 2*via* route (a) only and the yield of 2 from 14. Thus, we modified the calculated values (Table 6) from Table 5 using the ratio of exchangeable deuterium source and H_2_O (see ESI, Table S1†). As shown in Table 6, we indicated the yield of 2*via* S_N_2 (route (a)) only, and the yield of 11 from 14 if reaction mixture contains only exchangeable deuterium source and no H_2_O.

**Table 5:** Experimental evidence^a^^,^^b^

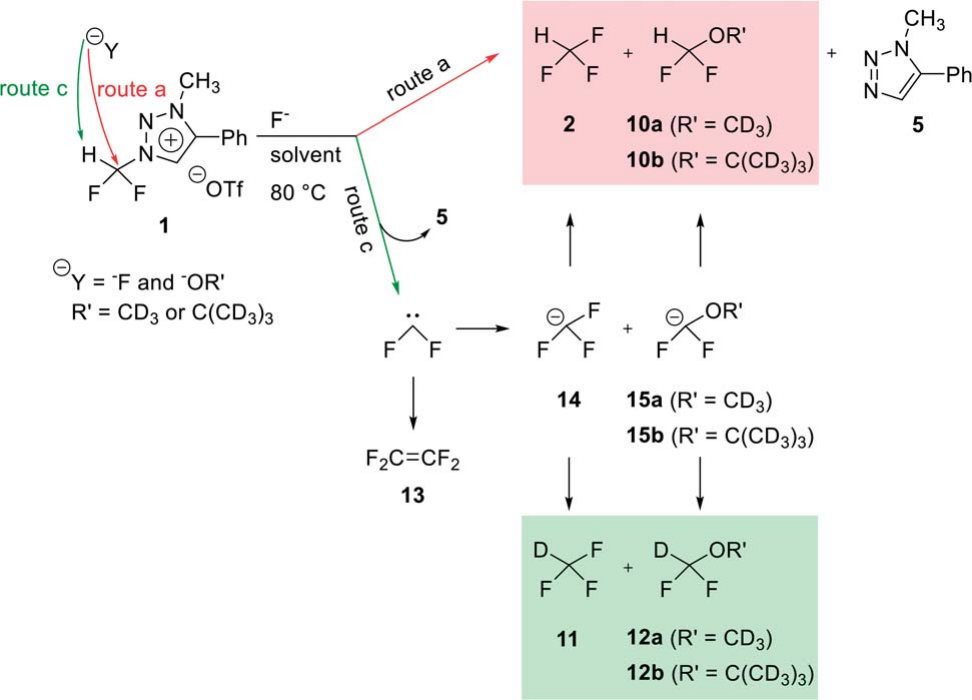								
Entry	F^−^	Solvent	Time	Yield^c^ (%)				
				2	11	10a	12a	13
1	TBAF	CD_3_OD	17 h	10	22	10	41	17
2	TBAF	CD_3_CN/CD_3_OD (6.5 : 1)	20 min	36	23	21	15	5
3	CsF	CD_3_CN/CD_3_OD (6.5 : 1)	5 h	7.8	51.5	4.2	34.9	1.6
4	CsF	CD_3_CN/CD_3_OD (17.8 : 1)	5 h	34	38	12	15	1
5	CsF	CD_3_CN/(CD_3_)_3_COD (6.5 : 1)	5 h	31.5	65.9	—	—	0
6	CsF	CD_3_CN/(CD_3_)_3_COD (17.8 : 1)	5 h	65	35	—	—	0

a All reactions were carried out on a 0.084 mmol reaction scale of triazolium precursor 1 in 0.75 mL of solvent in a sealed NMR tube. TBAF·3H_2_O or CsF (6 equiv.) was used as F^−^ source.

b Traces of 10b and 12b were detected by ^19^F NMR.

c ^19^F NMR yield.

**Table 6:** Modified data from Table 5^a^^,^^b^

Entry	F^−^	Solvent	Time	Yield^c^ (%)				
				2	11	10a	12a	13
1	TBAF	CD_3_OD	17 h	4.9	27.1	0.4	50.6	17
2	TBAF	CD_3_CN/CD_3_OD (6.5 : 1)	20 min	21.5	37.5	11.6	24.4	5
3	CsF	CD_3_CN/CD_3_OD (6.5 : 1)	5 h	6.6	52.7	3.4	35.7	1.6
4	CsF	CD_3_CN/CD_3_OD (17.8 : 1)	5 h	32.5	39.5	11.4	15.6	1
5	CsF	CD_3_CN/(CD_3_)_3_COD (6.5 : 1)	5 h	22.9	74.5	—	—	0
6	CsF	CD_3_CN/(CD_3_)_3_COD (17.8 : 1)	5 h	58.3	41.7	—	—	0

a All reactions were carried out on a 0.084 mmol reaction scale of triazolium precursor 1 in 0.75 mL of solvent in a sealed NMR tube. TBAF·3H_2_O or CsF (6 equiv.) was used as F^−^ source.

b Traces of 10b and 12b were detected by ^19^F NMR.

c Modified values from Table 5, except for 13: yields of 2 and 10a*via* route (a) only; yields of 11 and 12a from 14 and 15a respectively if reaction mixture contains only deuterium source and no H_2_O.

When 1 reacted with TBAF in CD_3_OD, 2 and 10a were observed in ^1^H NMR (see ESI†). When S_N_2 reaction occurred between 1 and CD_3_O^−^, 10a would be formed. We observed that in ^19^F NMR experiments, not only 2 and 10a*via* route (a), but also 11 and 12a*via* route (c) were obtained. 11 was formed in larger quantity than 2, and more of 12a was formed than 10a (Table 6, entry 1). When solvent was changed from CD_3_OD to CD_3_CN/CD_3_OD (6.5 : 1), the S_N_2 reaction rates significantly increased, the reaction being complete after 20 min at 80 °C, as 2 and 10a were detected by ^1^H NMR. Thus it seems that increasing the amount of acetonitrile leads to faster S_N_2 reaction. To reduce the amount of moisture in the reaction mixture, we carried out the reaction using CsF as the source of F^−^ under the Ar atmosphere. It was found that the reaction was complete after 5 h in CD_3_CN/CD_3_OD (6.5 : 1) (entry 3).

Compared to the reaction of entry 2, production of 2 was slower probably because the nucleophilicity of F^−^ in CsF was lower than that in TBAF. To further increase the rates of S_N_2 reactions, we changed the ratio of CD_3_CN/CD_3_OD to 17.8 : 1 (entry 4). Compared to the reaction of entry 3, the formation of 2*via* only route (a) increased to 32.5% and the yield of 10a increased to 11.4%. As 2 : 11 was 1 : 1.22, the yields of the two products were similar.

We have reported in our previous work^14^ that the rates of fluorination increased as the steric hindrance of alcoholic solvent increased. Thus we changed the deuterium source from CD_3_OD to (CD_3_)_3_COD. When CD_3_CN/(CD_3_)_3_COD (6.5 : 1) was used, compared to the reaction of entry 3, the yield of 2*via* only route (a) increased from 6.6% to 22.9%, and 2 : 11 was formed in a ratio of 1 : 3.25 (entry 5). When CD_3_CN/(CD_3_)_3_COD (17.8 : 1) was used, compared to the reaction of entry 4, the yield of 2 increased from 32.5% to 58.3% (entry 6). We found that 2*via* only route (a) was formed predominantly than 11, as 2 : 11 was formed in the ratio of 1.40 : 1.

Thus, in CD_3_CN/(CD_3_)_3_COD (17.8 : 1) as solvent, S_N_2 pathway (route (a)) was superior to route (c). A small amount of *t*-butanol-*d*_10_ was used because the degree of route (c) (formation of difluorocarbene) could not be measured without it.

## Conclusions

In summary, the fluorination reactions have been developed for the triazolium precursor to generate [^18^F]fluoroform presumably having high molar activity *via* S_N_2 pathway. The precursor was very stable in acetonitrile and decomposed very slowly in DMF. Fluoroform was formed rapidly at rt in 10 min by fluorination of the precursor with TBAF (6 equiv.). Fluoride attacked the electropositive carbon of difluoromethyl group, whereas 1-phenylpiperazine attacked *N*3-methyl group that was less sterically hindered. We also observed the nucleophilic selectivity toward the precursor. When benzotriazolium precursor and CsF were used, we confirmed that C5 proton acting as a hydrogen bond donor affected the selective generation of fluoroform. Moreover, NMR studies were in favor of S_N_2 pathway that was superior to formation of difluorocarbene in the reaction of the triazolium precursor using CsF and CD_3_CN/(CD_3_)_3_COD (17.8 : 1). Fluorination of the precursor *via* S_N_2 will be possible to generate [^18^F]fluoroform with improved molar activity. Based on these very positive preliminary results, our investigations for the synthesis of [^18^F]fluoroform with high *A*_m_ are ongoing.

## Conflicts of interest

There are no conflicts to declare.

## Supplementary Material

RA-011-D0RA09827B-s001
