# Supplementary material for: Mechanistic study of nucleophilic fluorination for the synthesis of fluorine-18 labeled fluoroform with high molar activity from N-difluoromethyltriazolium triflate
Source: RSC Adv. 2021 Feb 3;11(11):6099–106. doi: 10.1039/d0ra09827b (PMC8694808; doi:10.1039/d0ra09827b)
Supplement: RA-011-D0RA09827B-s001 [file RA-011-D0RA09827B-s001.pdf]

Electronic Supplementary Material (ESI) for RSC Advances.
This journal is © The Royal Society of Chemistry 2020

Supporting Information

Mechanistic Study of Nucleophilic Fluorination for the Synthesis of Fluorine-18 Labeled Fluoroform with High Molar Activity from *N*-Difluoromethyltriazolium Triflate

Jin Young Chai,[a] Hyojin Cha,[a] Sung-Sik Lee,[b] Young-Ho Oh,[b] Sungyul Lee,[b,] and Dae Yoon Chi[a,*]*

[a]Department of Chemistry, Sogang University, 35 Baekbeomro Mapogu, Seoul 04107, Korea

[b]Department of Applied Chemistry, Kyung Hee University, 1732 Deogyeong-daero, Giheung-gu, Yongin-si, Gyeonggi-do 17104, Korea

dychi@sogang.ac.kr

Table of Contents

1. General information ………...……………………………………………………………… S2

2. Experimental section ………..……………………………………………………………..…. S2

3. References ………...……………………………………………………………………...…. S6

4. ^{1}H NMR experiments for Table 1–4………..………………………………………………… S7

5. ^{1}H NMR experiments, ^{19}F NMR, and Table S1 for Table 5……………...………………….…..... S21

6. Determination of modified values for Table 6.……………...…………………….…....................... S28

7. NMR spectra of compounds **1**, **5**, **7**, **8**…………………...……...…………………………...…..… S29

8. Cartesian coordinates……………………………………………………………………..S34

9. Natural population analysis…………………………………………………………………S48

1. General information

All the chemicals were purchased from commercial sources and used without further purification. Flash column chromatography was performed on Merck silica gel 60 (230–400 mesh). Melting points were determined on a Kruss melting point apparatus. ^{1}H and ^{13}C NMR spectra were recorded on Varian 400 MHz NMR spectrometer. ^{19}F NMR spectra were recorded on Varian 500 MHz NMR spectrometer. ^{1}H NMR chemical shifts were determined relative to deuterated solvent peaks (δ = CD_3CN 1.94 ppm, $CDCl_3$ 7.26 ppm and CD_3OD 3.31 ppm). ^{13}C NMR chemical shifts were determined relative to deuterated solvent peaks (δ = CD_3CN 1.32 ppm and $CDCl_3$ 77.16 ppm). ^{19}F NMR chemical shifts were determined relative to $PhCF_3$ at δ -64.24 ppm (in CD_3OD) or δ -63.10 ppm (in CD_3CN).[1] The High resolution mass spectra were obtained from Bruker Compact Ultra High Resolution ESI Q-TOF mass spectrometer at Organic Chemistry Research Center of Sogang University. The elemental analysis was performed on Thermo Scientific FLASH 2000 elemental analyzer at Organic Chemistry Research Center of Sogang University.

2. Experimental section

Synthesis of Precursors 1 and 7.

*1-(Difluoromethyl)-4-phenyl-1H-1,2,3-triazole (**3**).*

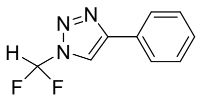

Prepared by a literature procedure.[2]

*1-(Difluoromethyl)-3-methyl-4-phenyl-1H-1,2,3-triazol-3-ium Triflate (**1**).*

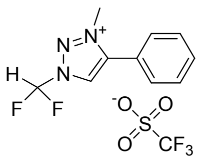

To 1-(difluoromethyl)-4-phenyl-1*H*-1,2,3-triazole (**3**, 500 mg, 2.56 mmol) dissolved in CH_2Cl_2 (10 mL) in a pressure tube was added methyl trifluoromethanesulfonate (MeOTf, 0.820 mL, 7.48 mmol) and the pressure tube was tightly capped. The reaction mixture was stirred at 50 °C for 3 h. The mixture was transferred to a 20 mL vial and dried in vacuo to obtain 727 mg of the product as a white solid (79%): mp 104–109 °C; ^{1}H NMR (400 MHz, CD_3CN) δ 8.95 (s, 1H), 7.94 (t, J = 57.4 Hz, 1H), 7.75–7.64 (m, 5H), 4.29 (s, 3H); ^{13}C NMR (100 MHz, CD_3CN) δ 145.4, 133.3, 130.64, 130.61, 127.9, 122.5, 121.9 (q, J = 318.7 Hz), 111.0 (t, J = 264.3 Hz), 40.7; ^{19}F NMR (470 MHz, CD_3CN) δ -79.24 (s, 3F), -99.91 (d, J = 57.8 Hz, 2F). HRMS (ESI/Q-TOF) m/z: $[M-OTf]^+$ Calcd for $C_{10}H_{10}F_2N_3$ 210.0837; Found 210.0831. Anal. Calcd for $C_{11}H_{10}F_5N_3O_3S$: C, 36.77; H, 2.81; N, 11.70. Found: C, 36.68; H, 2.97; N, 11.68.

*1-(Difluoromethyl)-1H-benzo[d][1,2,3]triazole (**9**).*

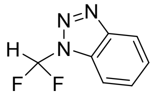

Prepared by a literature procedure.[2]

1-(Difluoromethyl)-3-methyl-1H-benzo[d][1,2,3]triazol-3-ium Triflate (7).

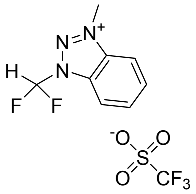

To 1-(difluoromethyl)-1*H*-benzo[*d*][1,2,3]triazole (**9**, 84.6 mg, 0.50 mmol) dissolved in CH_2Cl_2 (2 mL) in a screw top vial was added MeOTf (0.160 mL, 1.46 mmol) and the screw top vial was tightly capped. The reaction mixture was stirred at 50 °C for 50 min. The mixture was transferred to a 20 mL vial and dried in vacuo to obtain 156 mg of the product as a brown solid (93%): mp 125–138 °C; ^{1}H NMR (400 MHz, CD_3CN) δ 8.27-8.23 (m, 2H), 8.26 (t, *J* = 56.8 Hz, 1H), 8.14-8.05 (m, 2H), 4.67 (s, 3H); ^{13}C NMR (100 MHz, CD_3CN) δ 137.2, 134.8, 133.6, 133.2, 121.9 (q, *J* = 319.0 Hz), 115.6, 114.3, 112.1 (t, *J* = 261.5 Hz), 40.2; ^{19}F NMR (470 MHz, CD_3CN) δ -79.16 (s, 3F), -99.52 (d, *J* = 56.9 Hz, 2F). HRMS (ESI/Q-TOF) m/z: [M-OTf]$^+$ Calcd for $C_8H_8F_2N_3$ 184.0681; Found 184.0682.

General Procedure for Fluorination Using Precursor 1.

Method A (nucleophile: CsF, Table 2, entry 1)

To CsF (25.5 mg, 0.168 mmol) in an NMR tube was added CD_3CN (0.15 mL). To precursor **1** (30.0 mg, 0.084 mmol) in a 20 mL vial was added CD_3CN (0.6 mL). The solution of the 20 mL vial was transferred to the NMR tube. The NMR tube was sealed with a cap and placed at 80 °C for 1 h. The reaction mixture was analyzed by ^{1}H NMR.

Method B (nucleophile: TBAF, Table 2, entry 8)

To TBAF·H_2O (131.8 mg, 0.504 mmol) in an NMR tube was added CD_3CN (0.4 mL). To precursor **1** (30.0 mg, 0.084 mmol) in a 20 mL vial was added CD_3CN (0.35 mL). The solution of the 20 mL vial was transferred to the NMR tube. The NMR tube was sealed with a cap and placed at rt for 10 min. The reaction mixture was analyzed by ^{1}H NMR.

*Fluoroform (**2**).*[3]

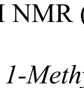

^{1}H NMR (400 MHz, CD_3CN) δ 6.79 (q, *J* = 79.6 Hz, 1H); ^{19}F NMR (470 MHz, CD_3OD) δ -80.43 (d, *J* = 79.4 Hz, 3F).

*1-Methyl-5-phenyl-1H-1,2,3-triazole (**5**).*[4]

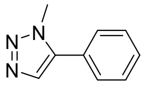

Purification by flash column chromatography (hexane/EtOAc = 50/50). Yellow oil: ^{1}H NMR (400 MHz, $CDCl_3$) δ 7.67 (s, 1H), 7.48–7.44 (m, 3H), 7.42–7.38 (m, 2H), 4.03 (s, 3H); ^{13}C NMR (100 MHz, $CDCl_3$) δ 138.1, 132.9, 129.5, 129.1, 128.6, 127.0, 35.5. HRMS (ESI/Q-TOF) m/z: $[M + Na]^+$ Calcd for $C_9H_9N_3Na$ 182.0689; Found 182.0688. (CAS 15966-55-9)

Procedure for Table 3, entry 1 (nucleophile: 1-phenylpiperazine)

To precursor **1** (30.0 mg, 0.084 mmol) in a 20 mL vial was added CD_3CN (0.4 mL). The solution of the 20 mL vial was transferred to an NMR tube. To 1-phenylpiperazine (40.9 mg, 0.252 mmol) in a 4 mL vial was added CD_3CN (0.35 mL). The solution of the 4 mL vial was transferred to the NMR tube. The NMR tube was sealed with a cap and placed at 80 °C for 5.5 h. The reaction mixture was analyzed by ^{1}H NMR.

Procedure for Experimental Evidence (Table 5, entry 1)

To TBAF·$3H_2O$ (159.0 mg, 0.504 mmol) in an NMR tube was added CD_3OD (0.4 mL). To precursor **1** (30.0 mg, 0.084 mmol) in a 20 mL vial was added CD_3OD (0.35 mL) and $PhCF_3$ (10.31 μL). The solution of the 20 mL vial was transferred to the NMR tube. The NMR tube was sealed with a cap and placed at 80 °C for 17 h. The reaction mixture was analyzed by ^{1}H NMR and ^{19}F NMR.

Procedure for Table 5, entry 2

To TBAF·$3H_2O$ (159.0 mg, 0.504 mmol) in an NMR tube was added CD_3OD (0.1 mL) and CD_3CN (0.3 mL). To precursor **1** (30.0 mg, 0.084 mmol) in a 20 mL vial was added CD_3CN (0.35 mL) and $PhCF_3$ (10.31 μL). The solution of the 20 mL vial was transferred to the NMR tube. The NMR tube was sealed with a cap and placed at 80 °C for 20 min. The reaction mixture was analyzed by ^{1}H NMR and ^{19}F NMR.

General Procedure for Experimental Evidence Using CsF

Method C (Table 5, entry 3)

An NMR tube was taken into the glovebox, and CsF (76.6 mg, 0.504 mmol) was added. The NMR tube was capped with a rubber septum and taken out of the glovebox. To precursor **1** (30.0 mg, 0.084 mmol) in a 20 mL vial was added CD_3CN (0.65 mL), CD_3OD (0.1 mL) and $PhCF_3$ (10.31 μL). The solution of the 20 mL vial was transferred to the NMR tube via a syringe. The rubber septum was replaced with a cap. The NMR tube was sealed with the cap and placed at 80 °C for 5 h. The reaction mixture was analyzed by ^{1}H NMR and ^{19}F NMR.

Procedure for Table 5, entry 4

Method C. To precursor **1** (30.0 mg, 0.084 mmol) in a 20 mL vial was added CD_3CN (0.71 mL), CD_3OD (40 μL) and $PhCF_3$ (10.31 μL).

Procedure for Table 5, entry 5

Method C. To precursor **1** (30.0 mg, 0.084 mmol) in a 20 mL vial was added CD_3CN (0.65 mL), $(CD_3)_3COD$ (0.1 mL) and $PhCF_3$ (10.31 μL).

Procedure for Table 5, entry 6

Method C. To precursor **1** (30.0 mg, 0.084 mmol) in a 20 mL vial was added CD_3CN (0.71 mL), $(CD_3)_3COD$ (40 μL) and $PhCF_3$ (10.31 μL).

*Fluoroform-d (**11**).*[5]

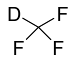

^{19}F NMR (470 MHz, CD_3OD) δ -81.19 (t, J = 12.2 Hz, 3F).

*(Difluoromethoxy)methane-d_3 (**10a**).*

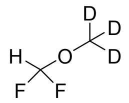

^{1}H NMR (400 MHz, CD_3OD) δ 6.34 (t, J = 75.8 Hz, 1H); ^{19}F NMR (470 MHz, CD_3OD) δ -87.95 (d, J = 75.7 Hz, 2F).

*(Difluoromethoxy)methane-d_4 (**12a**).*

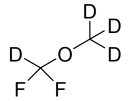

^{19}F NMR (470 MHz, CD_3OD) δ -88.78 (t, J = 11.8 Hz, 2F).

*2-(Difluoromethoxy)-2-(methyl-d_3)propane-1,1,1,3,3,3-d_6 (**10b**).*

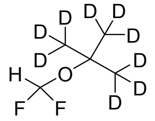

^{1}H NMR (400 MHz, $CD_3CN/(CD_3)_3COD$ (v/v= 6.5:1)) δ 6.45 (t, J = 77.2 Hz, 1H); ^{19}F NMR (470 MHz, $CD_3CN/(CD_3)_3COD$ (v/v= 6.5:1)) δ -77.01 (d, J = 77.1 Hz, 2F).

*2-(Difluoromethoxy-d)-2-(methyl-d_3)propane-1,1,1,3,3,3-d_6 (**12b**).*

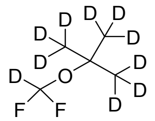

^{19}F NMR (470 MHz, $CD_3CN/(CD_3)_3COD$ (v/v= 6.5:1)) δ -77.77 (t, J = 11.8 Hz, 2F).

Procedure for Reaction Using Precursor 7 (Nucleophile: CsF, Table 4, entry 1)

To precursor **7** (30.0 mg, 0.090 mmol) in a 20 mL vial was added CD_3CN (0.75 mL). The solution of the 20 mL vial was transferred to an NMR tube. CsF (27.3 mg, 0.180 mmol) was added to the NMR tube. The NMR tube was sealed with a cap and placed at 80 °C for 24 h. The reaction mixture was analyzed by ^{1}H NMR.

*1-Methyl-1H-benzo[d][1,2,3]triazole (**8**).*

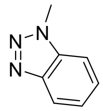

Purification by flash column chromatography (hexane/EtOAc = 70/30). Yellow solid: mp 62–64 °C; ^{1}H NMR (400 MHz, $CDCl_3$) δ 7.95 (d, *J* = 8.4 Hz, 1H), 7.44–7.37 (m, 2H), 7.28 (t, *J* = 8.2 Hz, 1H), 4.19 (s, 3H); ^{13}C NMR (100 MHz, $CDCl_3$) δ 145.8, 133.4, 127.2, 123.8, 119.7, 109.1, 34.1. HRMS (ESI/Q-TOF) m/z: $[M + Na]^+$ Calcd for $C_7H_7N_3Na$ 156.0532; Found 156.0532. (CAS 13351-73-0)

3. References

1. C. P. Rosenau, B. J. Jelier, A. D. Gossert and A. Togni, *Angew. Chem. Int. Ed.*, 2018, **57**, 9528-9533.
2. J. Y. Chai, H. Cha, H. B. Kim and D. Y. Chi, *Tetrahedron*, 2020, **76**, 131370.
3. (a) A. Zanardi, M. A. Novikov, E. Martin, J. Benet-Buchholz and V. V. Grushin, *J. Am. Chem. Soc.*, 2011, **133**, 20901-20913; (b) N. S. Golubev, G. S. Denisov, S. Macholl, S. N. Smirnov, I. G. Shenderovich and P. M. Tolstoy, *Z. Phys. Chem.*, 2008, **222**, 1225-1245; (c) J. Lin, Z. Li, J. Kan, S. Huang, W. Su and Y. Li, *Nat. Commun.*, 2017, **8**, 14353.
4. S. Koguchi and K. Izawa, *ACS Comb. Sci.*, 2014, **16**, 381-385.
5. (a) E. A. F. Fordyce, Y. Wang, T. Luebbers and H. W. Lam, *Chem. Commun.*, 2008, 1124-1126; (b) C. P. Johnston, T. H. West, R. E. Dooley, M. Reid, A. B. Jones, E. J. King, A. G. Leach and G. C. Lloyd-Jones, *J. Am. Chem. Soc.*, 2018, **140**, 11112-11124.

4. ^{1}H NMR experiments for Table 1–4

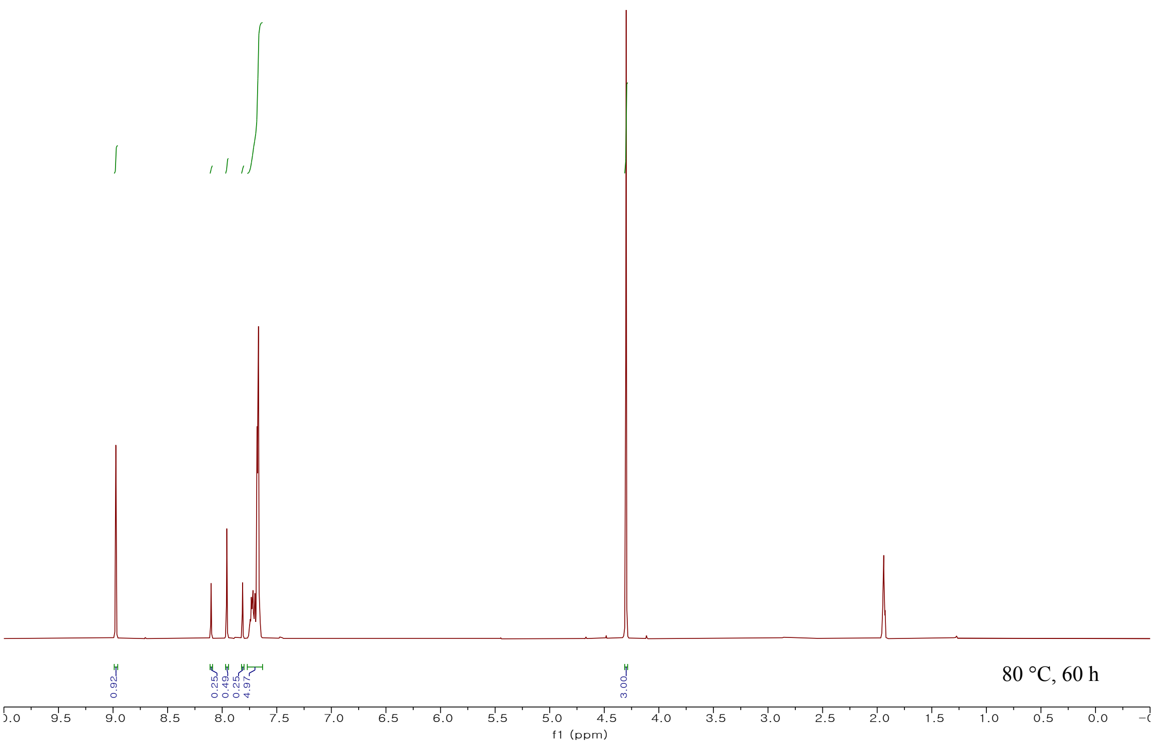

Figure S1. ^{1}H NMR experiment in CD_3CN to investigate stability of precursor **1** for Table 1, entry 1.

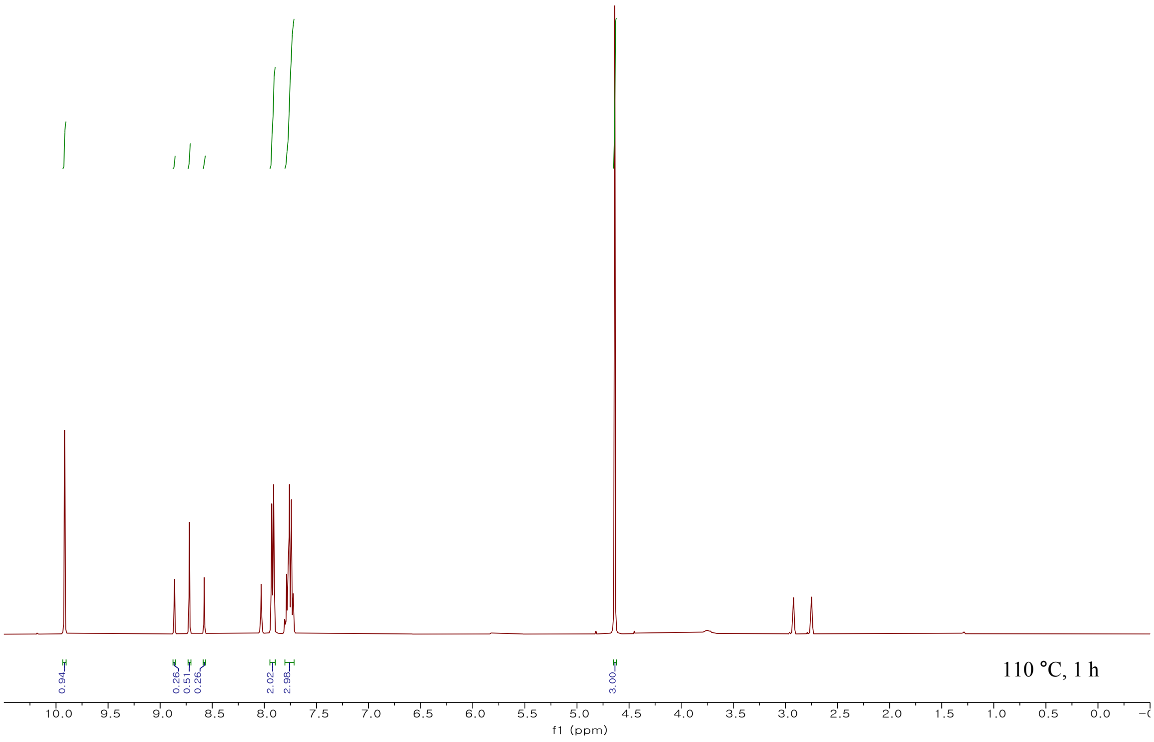

Figure S2. ^{1}H NMR experiment in DMF-d_7 to investigate stability of precursor **1** for Table 1, entry 2.

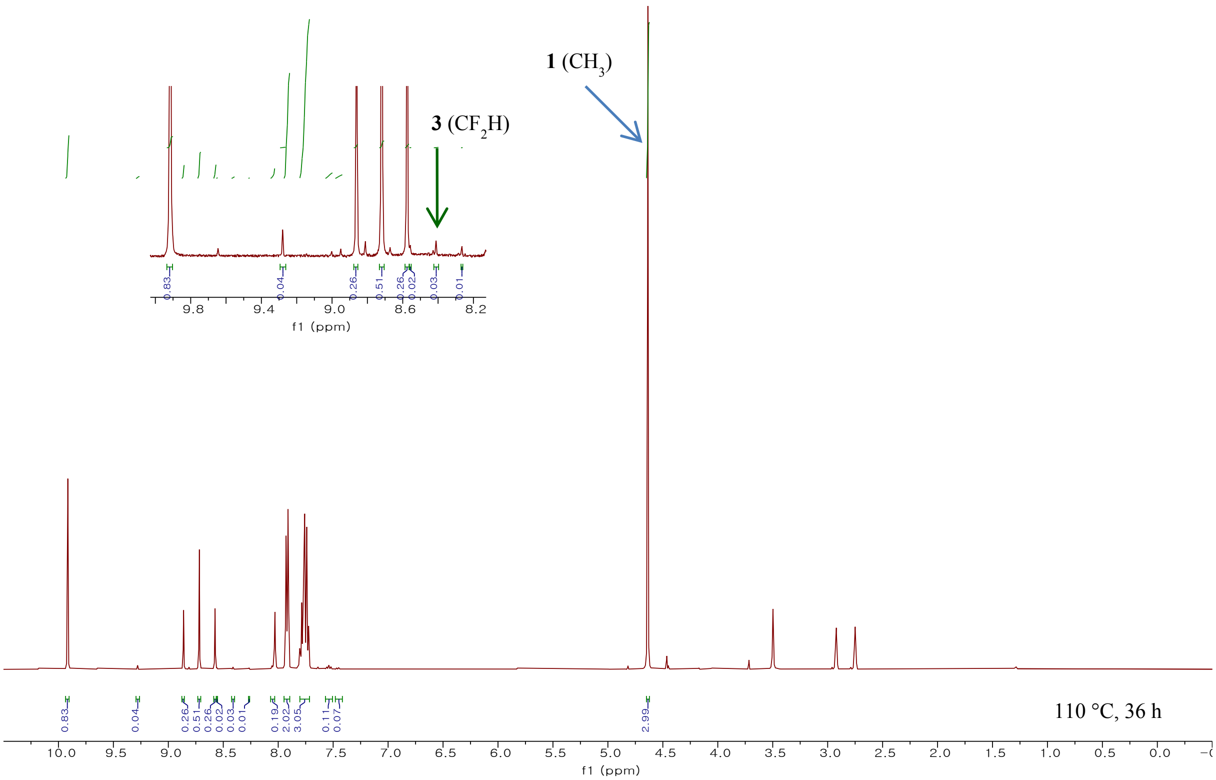

Figure S3. ^{1}H NMR experiment in DMF-d_7 to investigate stability of precursor **1** for Table 1, entry 3.

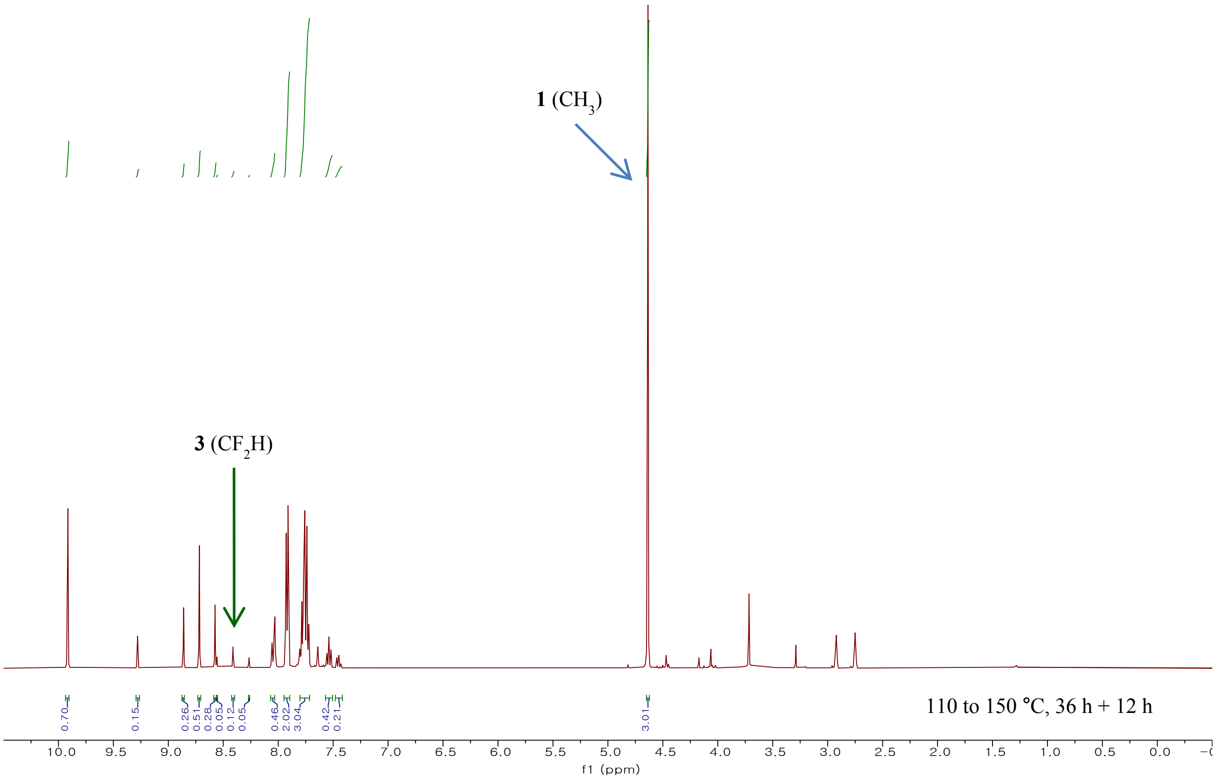

Figure S4. ^{1}H NMR experiment in DMF-d_7 to investigate stability of precursor **1** for Table 1, entry 4.

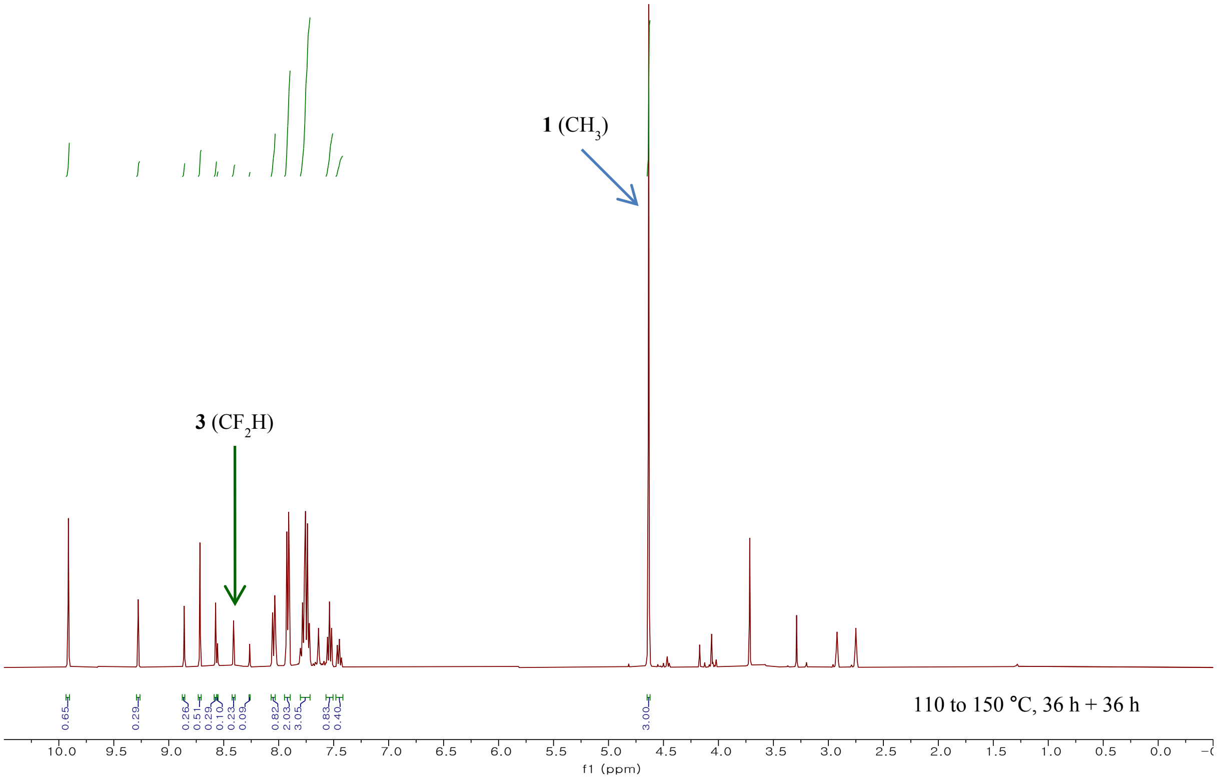

Figure S5. ^{1}H NMR experiment in DMF-d_7 to investigate stability of precursor **1** for Table 1, entry 5.

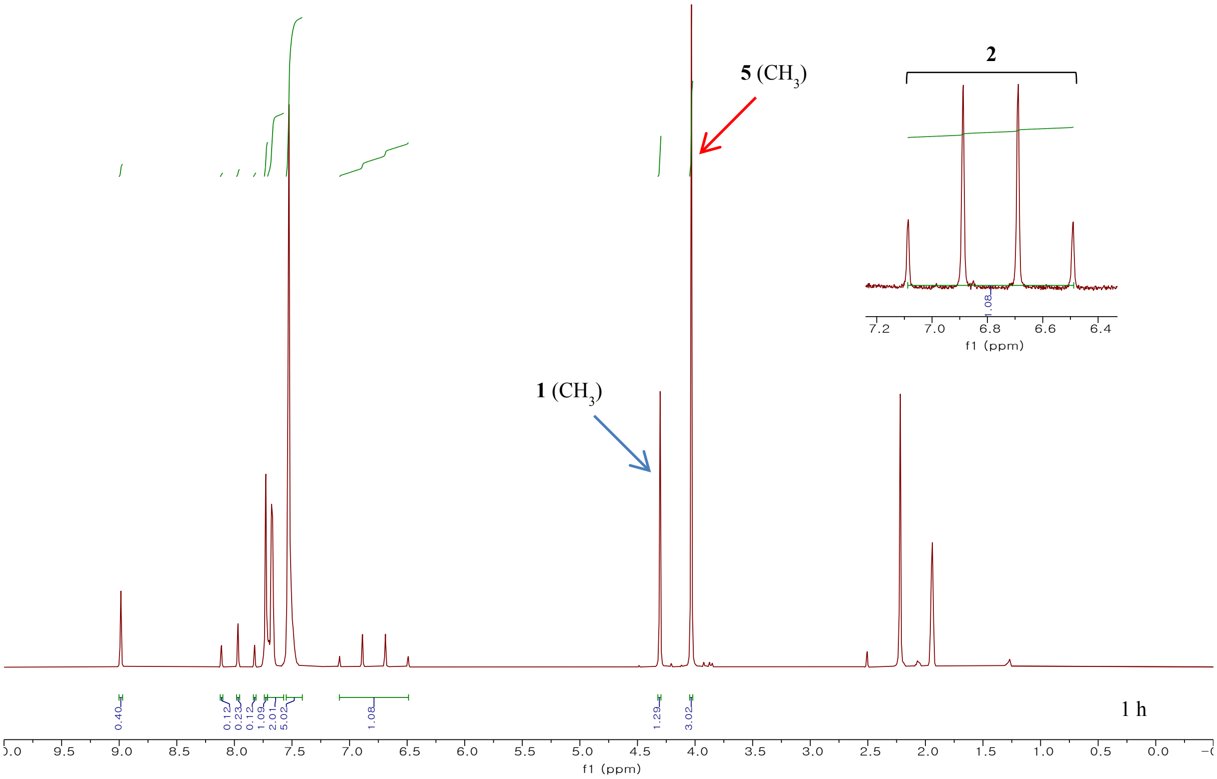

Figure S6. ^{1}H NMR experiment for optimization using precursor **1** for Table 2, entry 1.

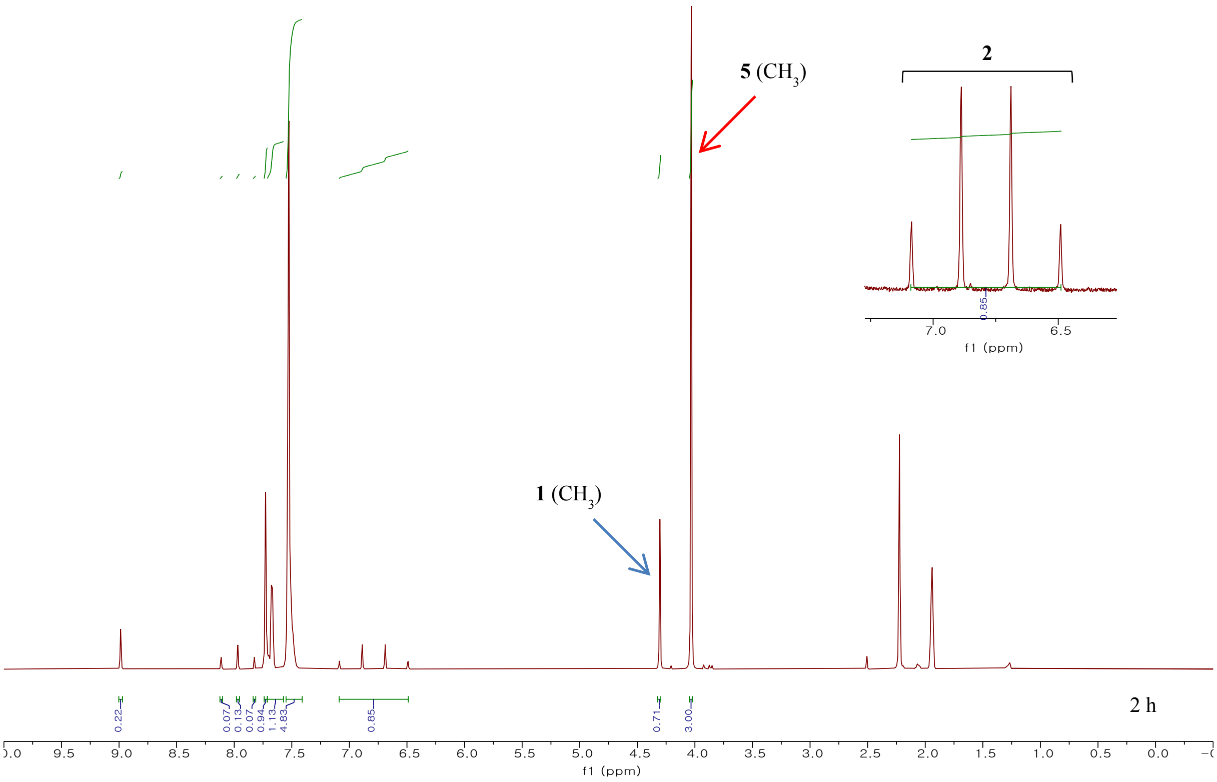

Figure S7. ^{1}H NMR experiment for optimization using precursor **1** for Table 2, entry 2.

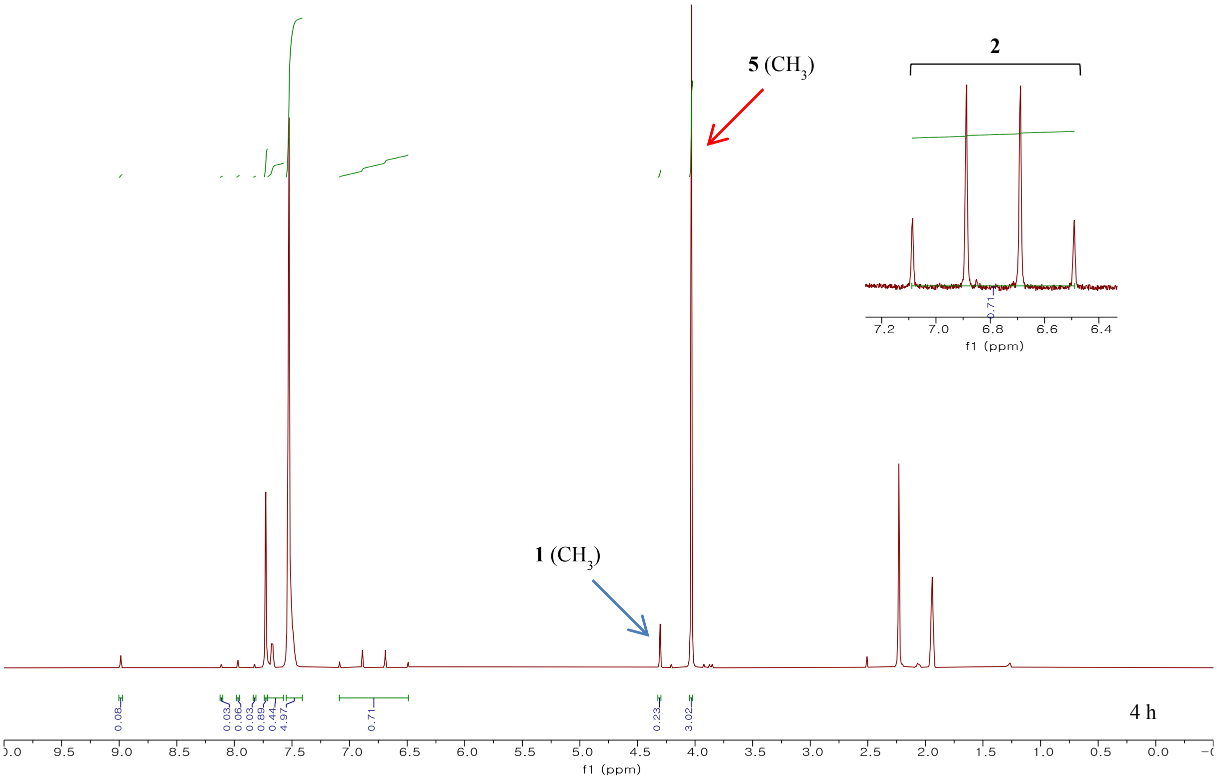

Figure S8. ^{1}H NMR experiment for optimization using precursor **1** for Table 2, entry 3.

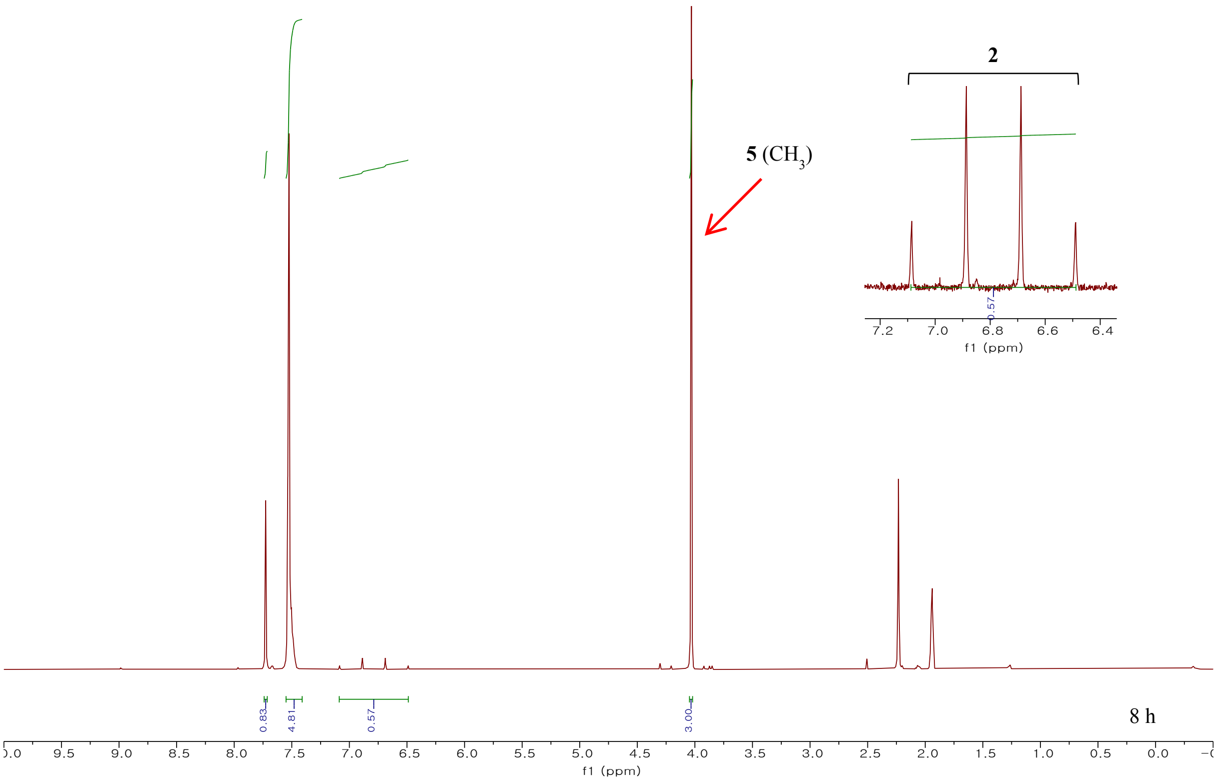

Figure S9. ^{1}H NMR experiment for optimization using precursor **1** for Table 2, entry 4.

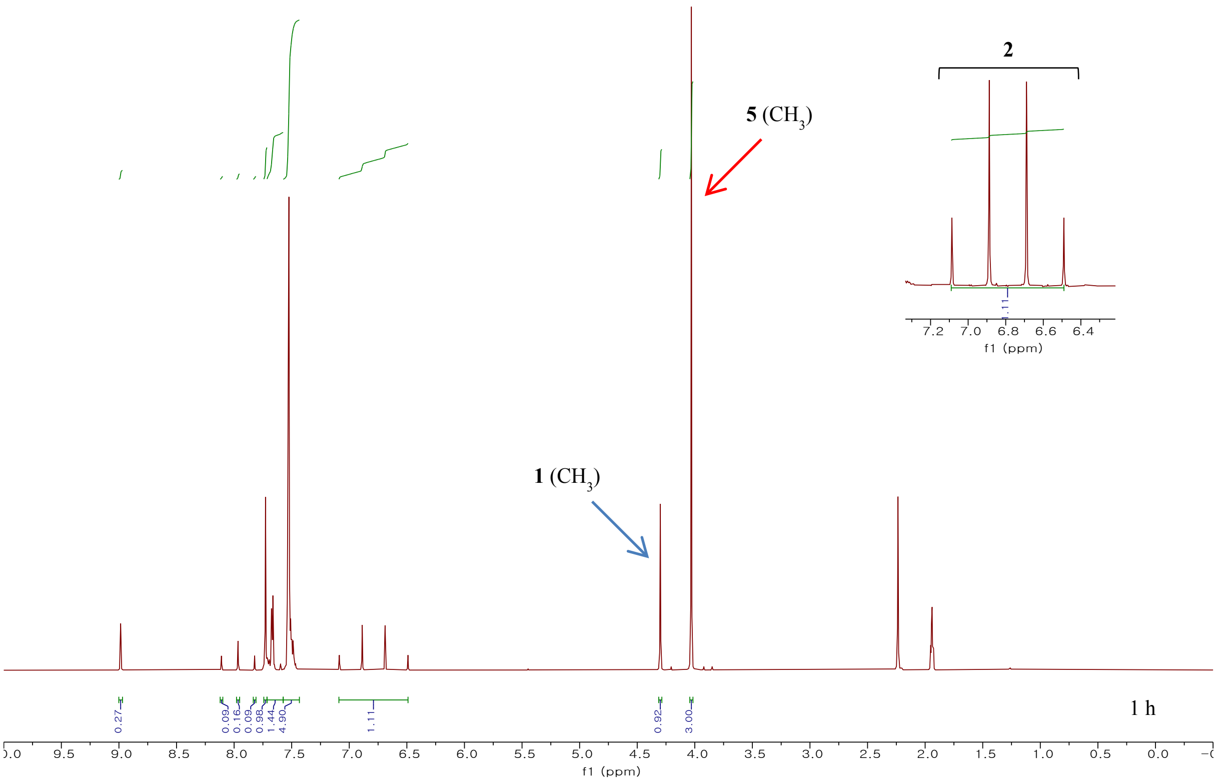

Figure S10. ^{1}H NMR experiment for optimization using precursor **1** for Table 2, entry 5.

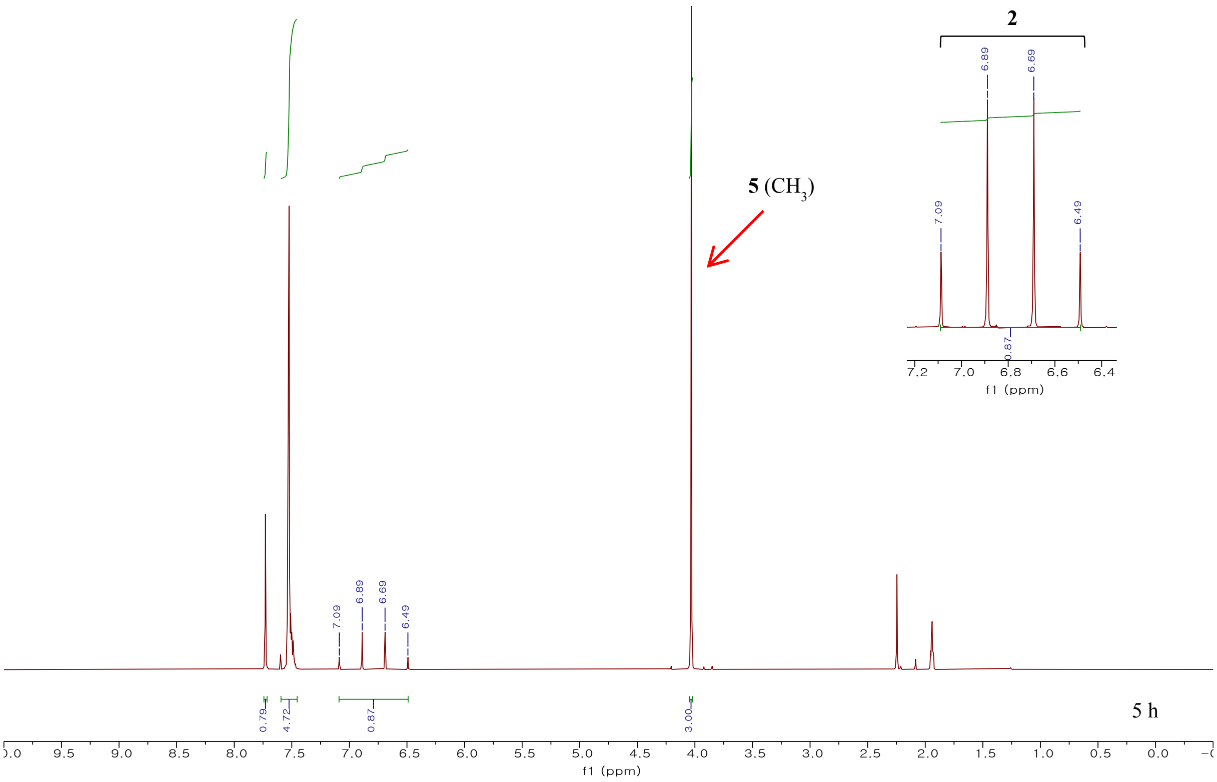

Figure S11. ^{1}H NMR experiment for optimization using precursor **1** for Table 2, entry 6.

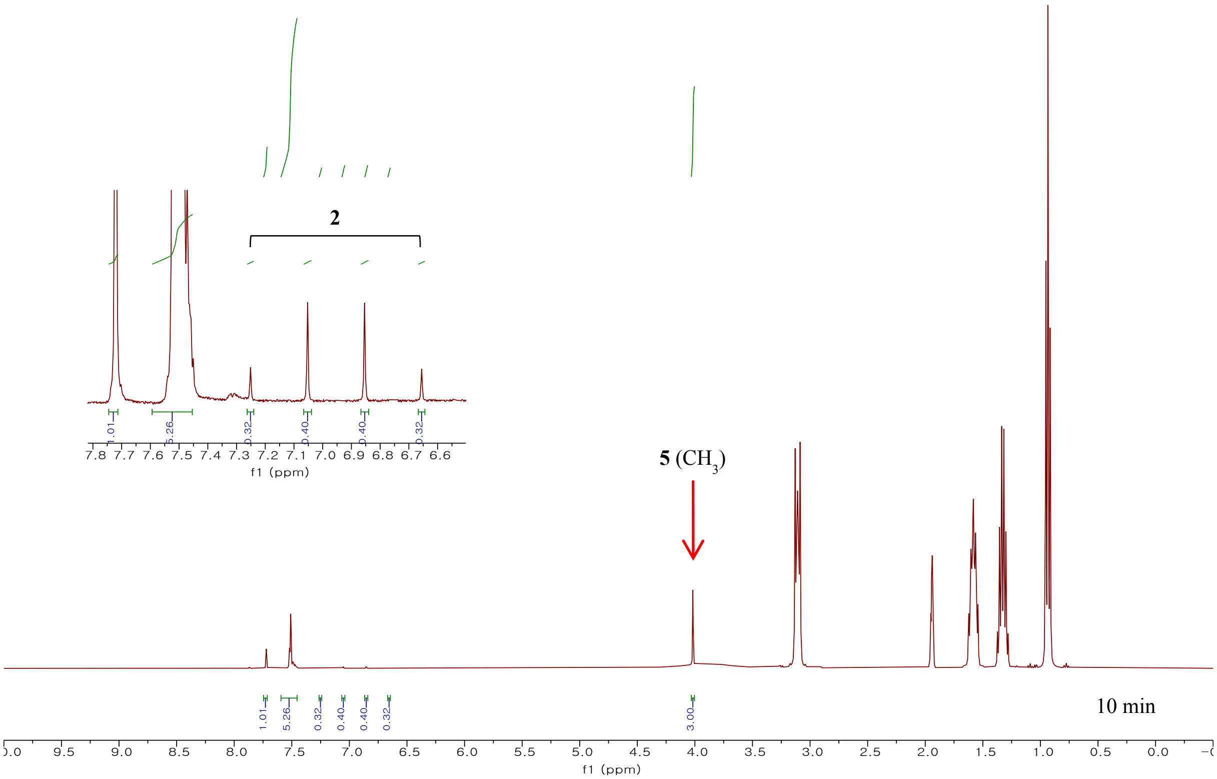

Figure S12. ¹H NMR experiment for optimization using precursor **1** for Table 2, entry 7.

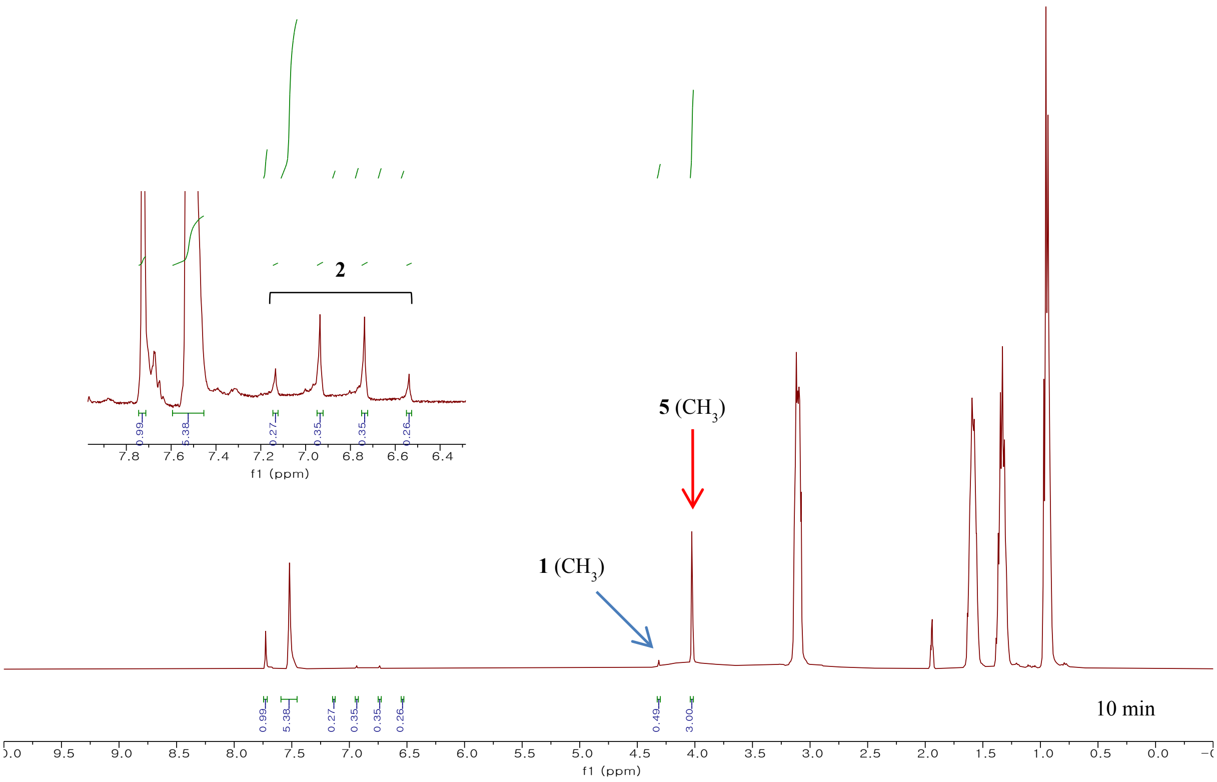

Figure S13. ¹H NMR experiment for optimization using precursor **1** for Table 2, entry 8.

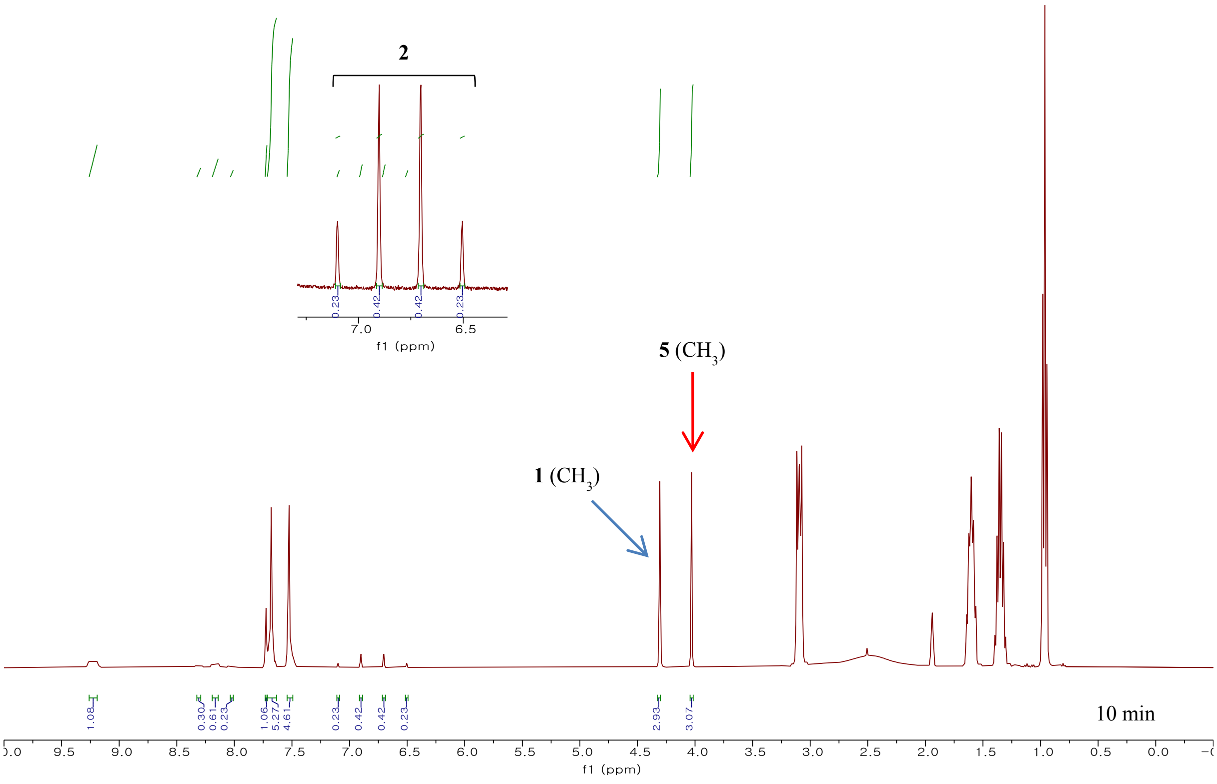

Figure S14. ^{1}H NMR experiment for optimization using precursor **1** for Table 2, entry 9.

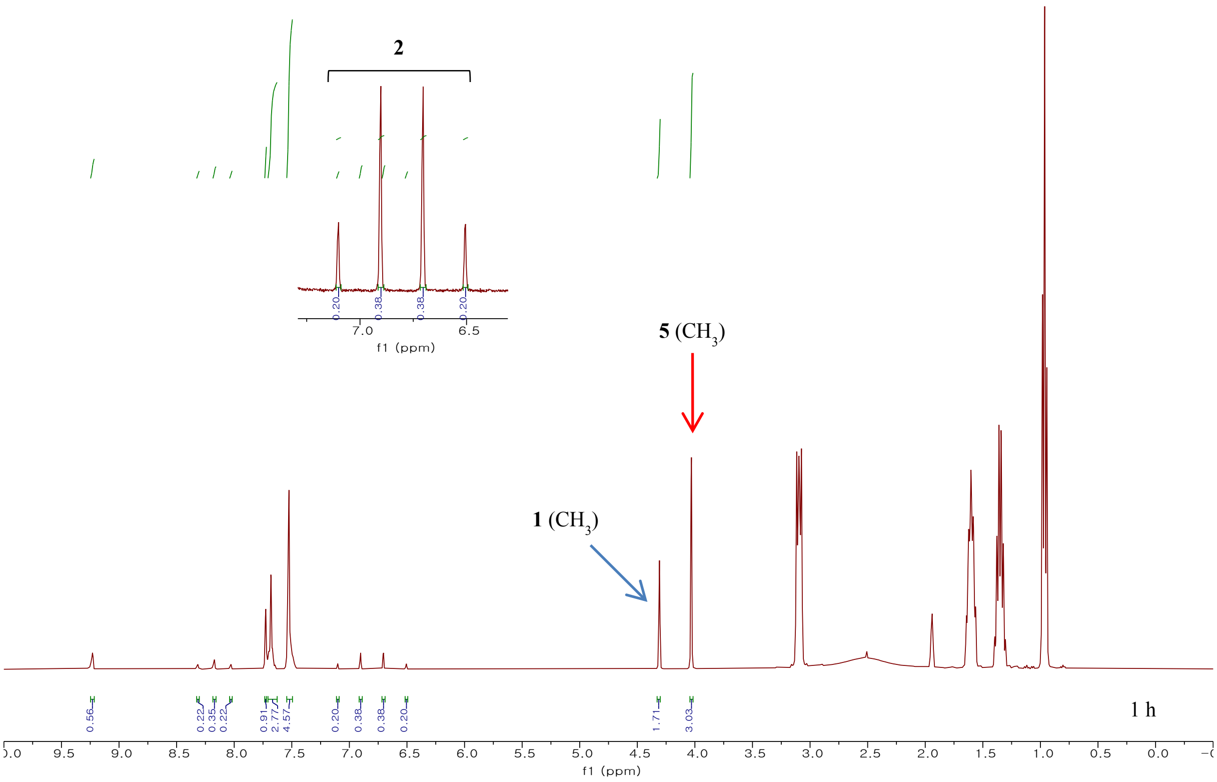

Figure S15. ^{1}H NMR experiment for optimization using precursor **1** for Table 2, entry 10.

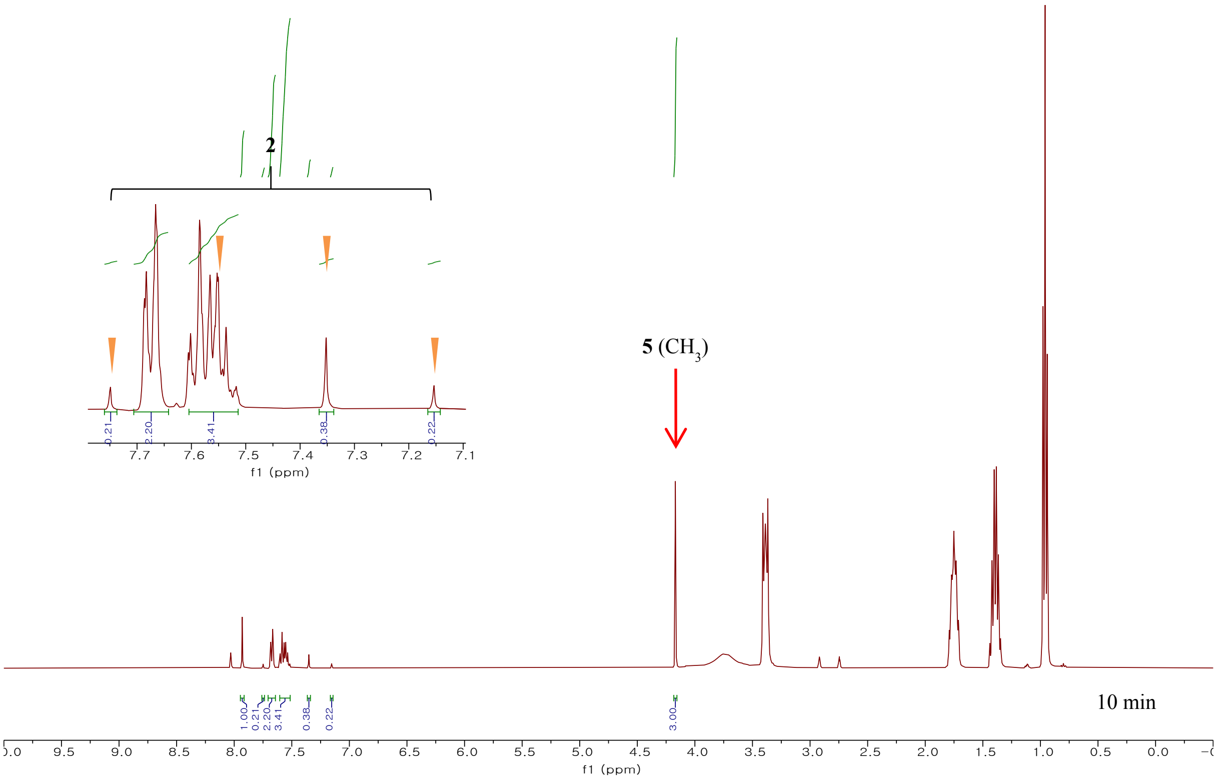

Figure S16. ^{1}H NMR experiment for optimization using precursor **1** for Table 2, entry 11.

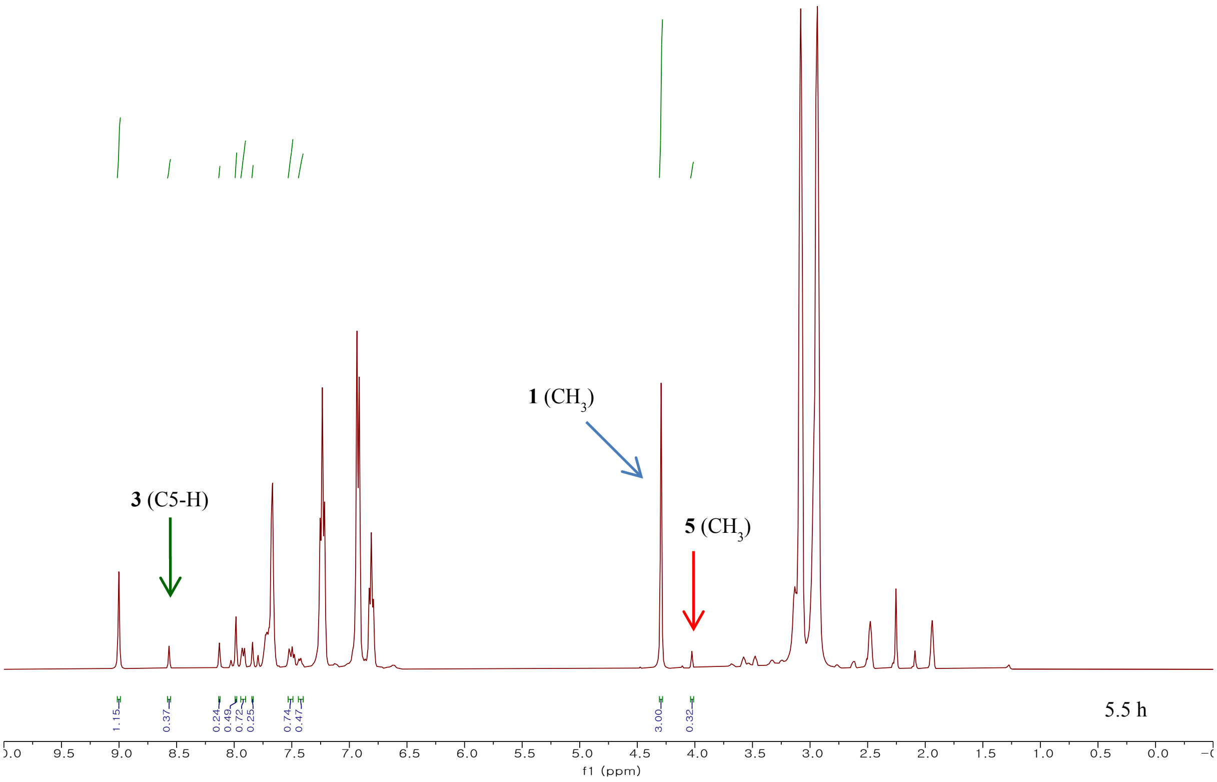

Figure S17. ^{1}H NMR experiment using precursor **1** for Table 3, entry 1 (nucleophile: 1-phenylpiperazine).

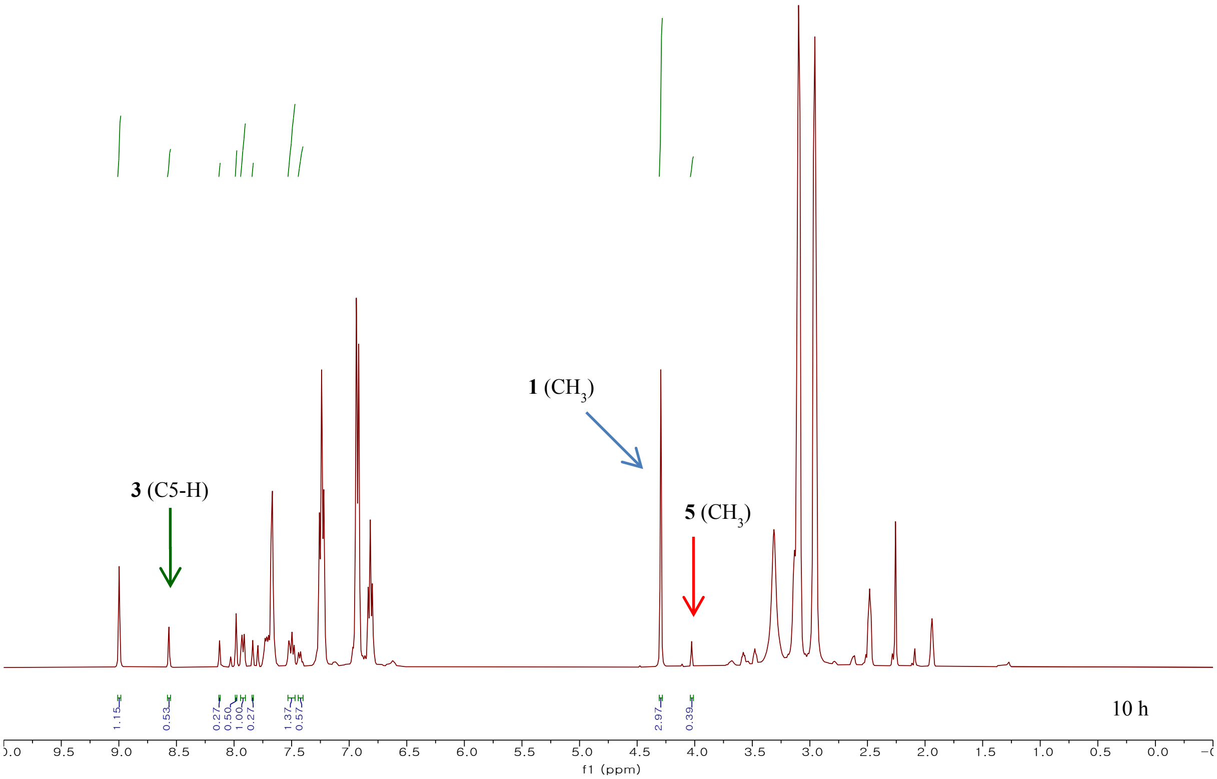

Figure S18. ^{1}H NMR experiment using precursor **1** for Table 3, entry 2 (nucleophile: 1-phenylpiperazine).

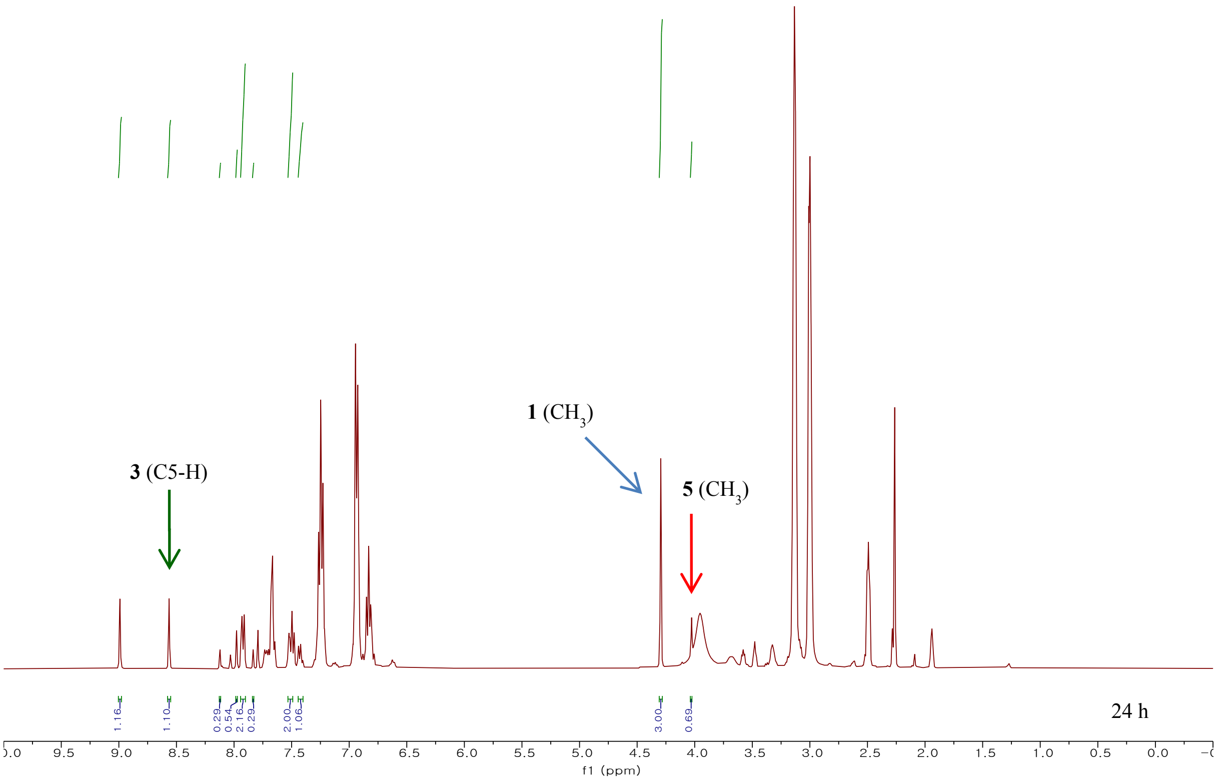

Figure S19. ^{1}H NMR experiment using precursor **1** for Table 3, entry 3 (nucleophile: 1-phenylpiperazine).

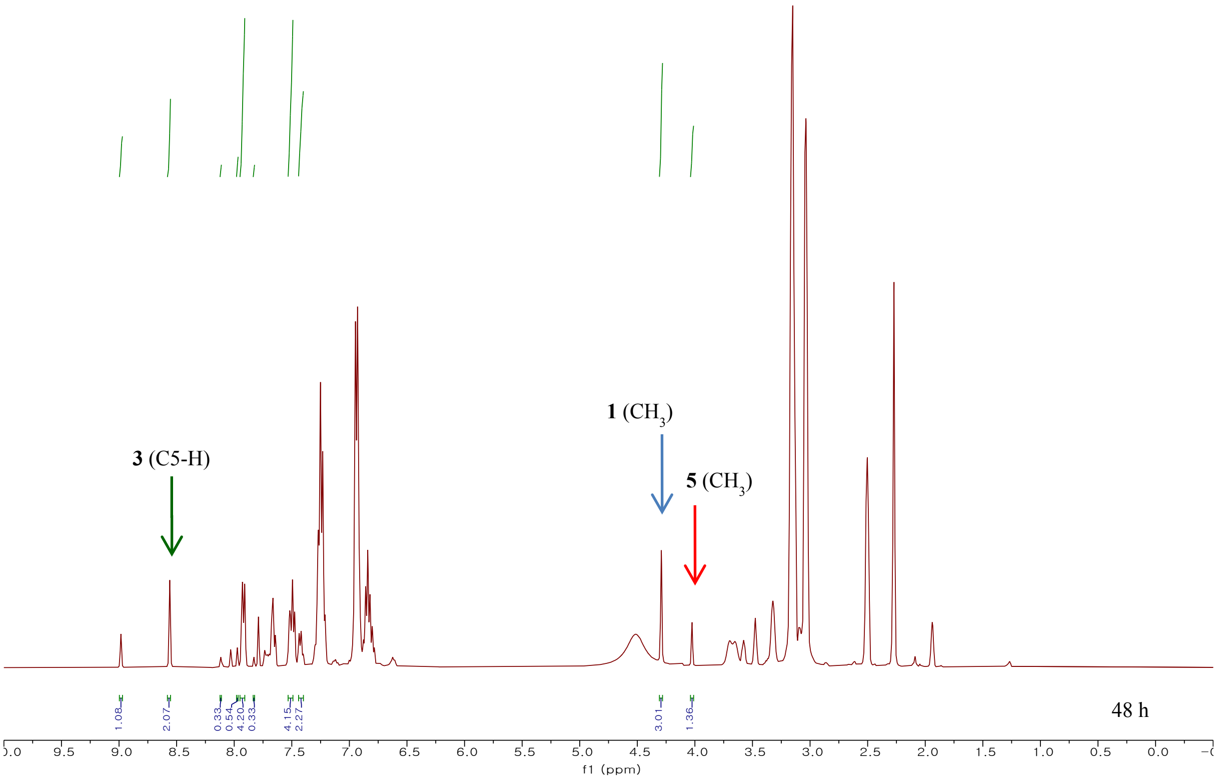

Figure S20. ^{1}H NMR experiment using precursor **1** for Table 3, entry 4 (nucleophile: 1-phenylpiperazine).

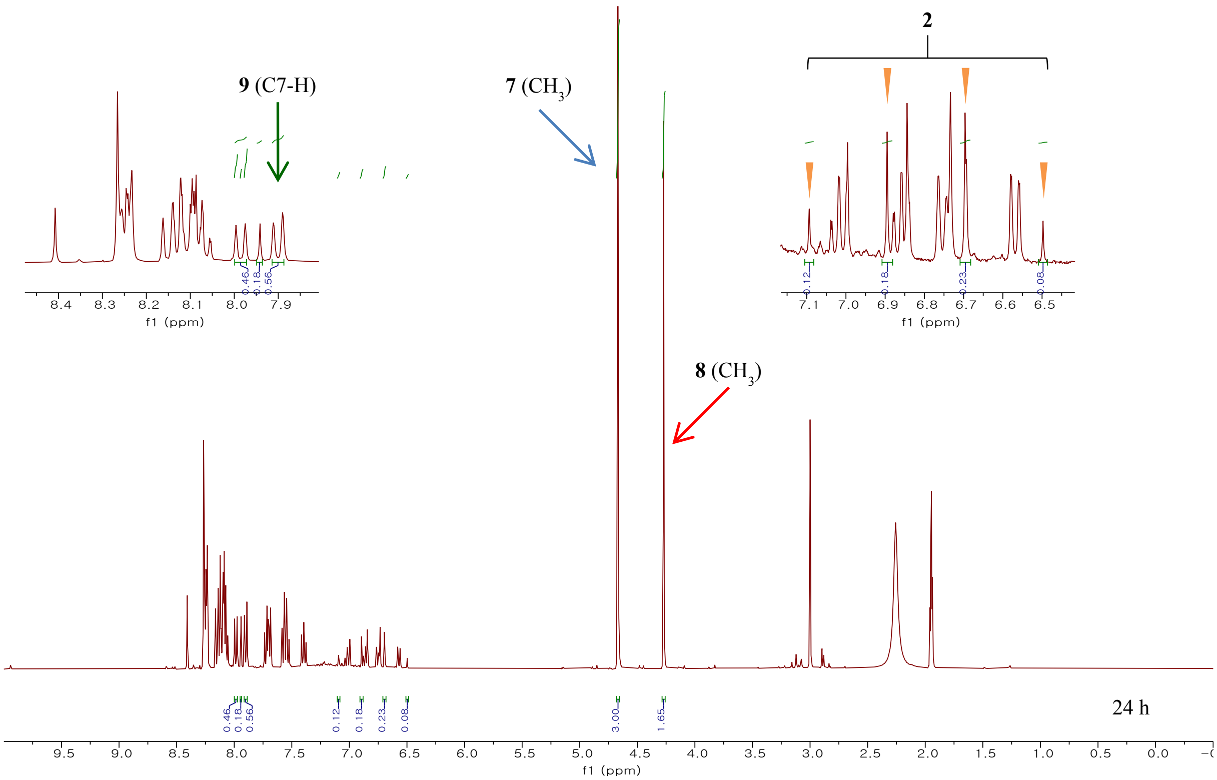

Figure S21. ^{1}H NMR experiment using precursor **7** for Table 4, entry 1 (nucleophile: CsF).

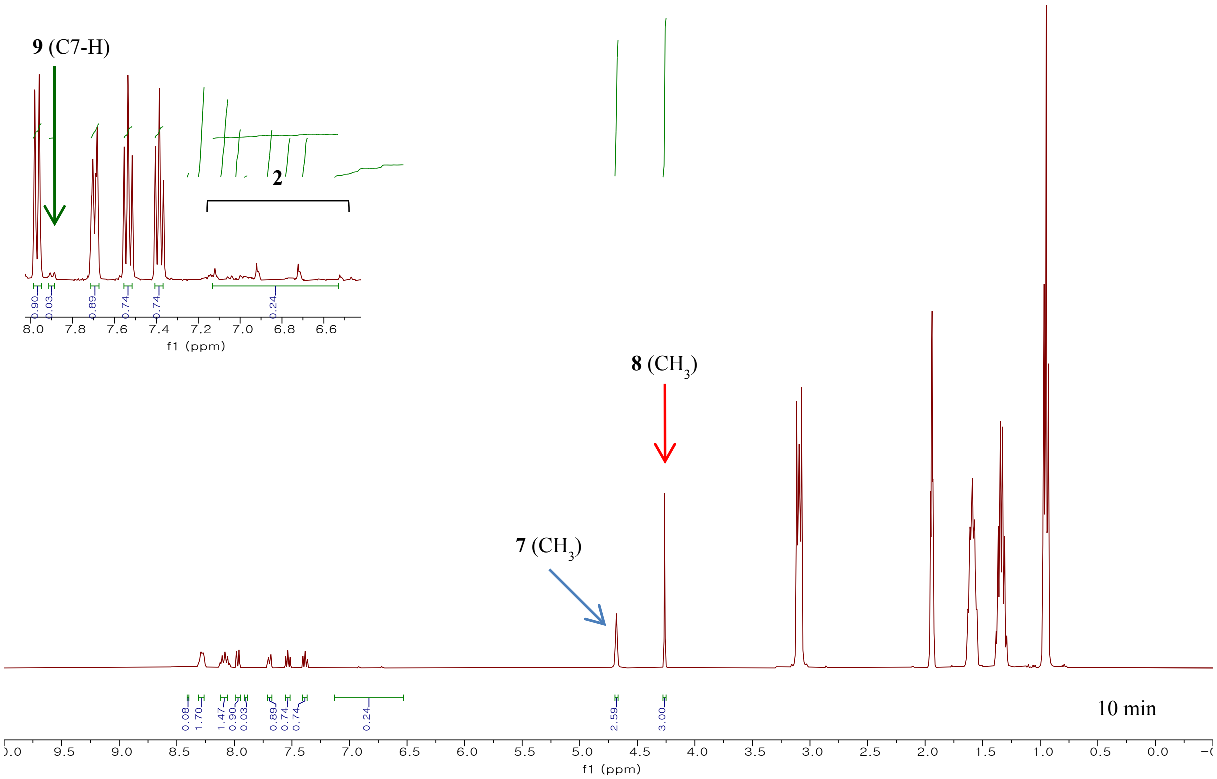

Figure S22. ^{1}H NMR experiment using precursor **7** for Table 4, entry 2 (nucleophile: TBAF).

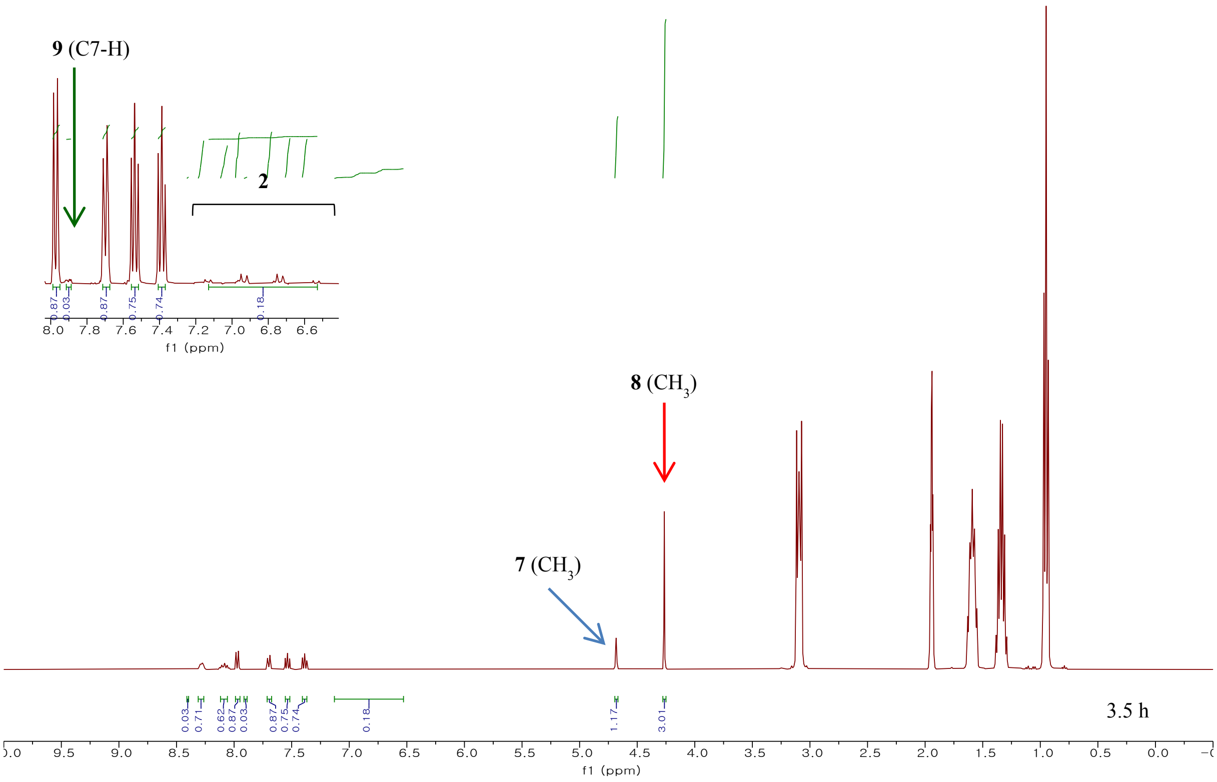

Figure S23. ^{1}H NMR experiment using precursor **7** for Table 4, entry 3 (nucleophile: TBAF).

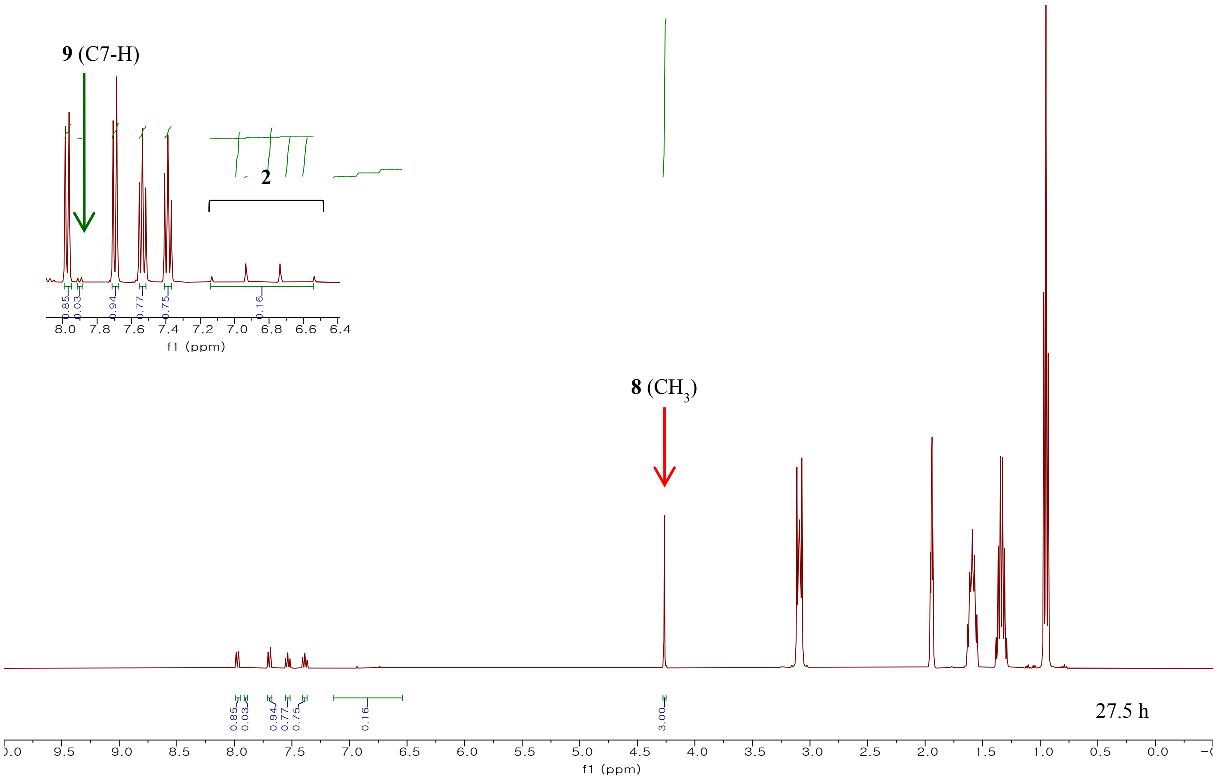

Figure S24. ^{1}H NMR experiment using precursor **7** for Table 4, entry 4 (nucleophile: TBAF).

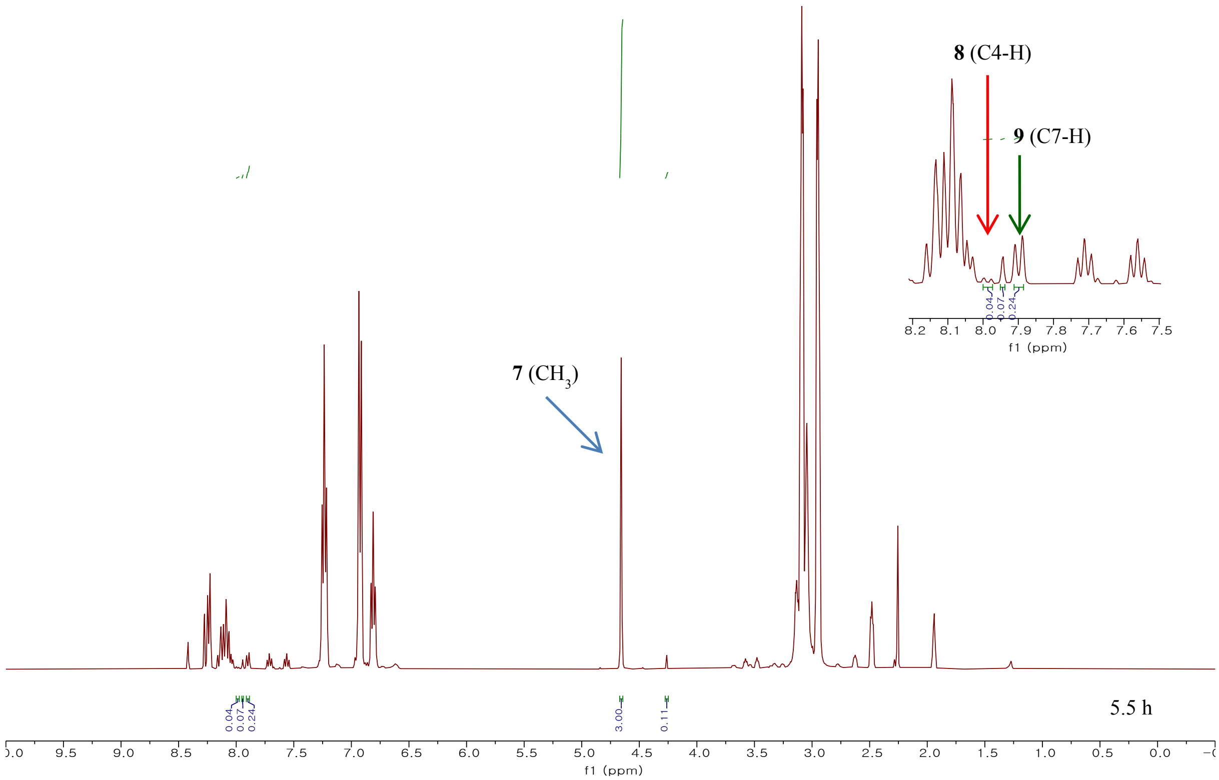

Figure S25. ^{1}H NMR experiment using precursor **7** for Table 4, entry 5 (nucleophile: 1-phenylpiperazine).

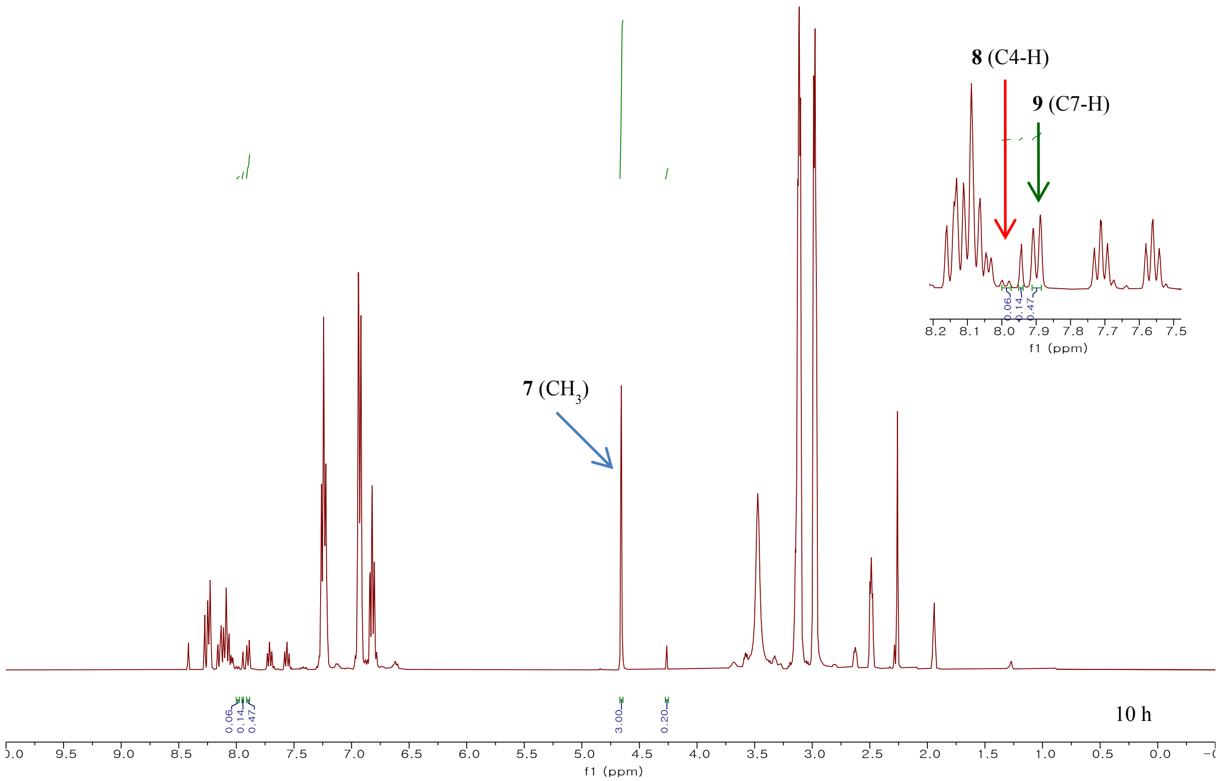

Figure S26. ^{1}H NMR experiment using precursor **7** for Table 4, entry 6 (nucleophile: 1-phenylpiperazine).

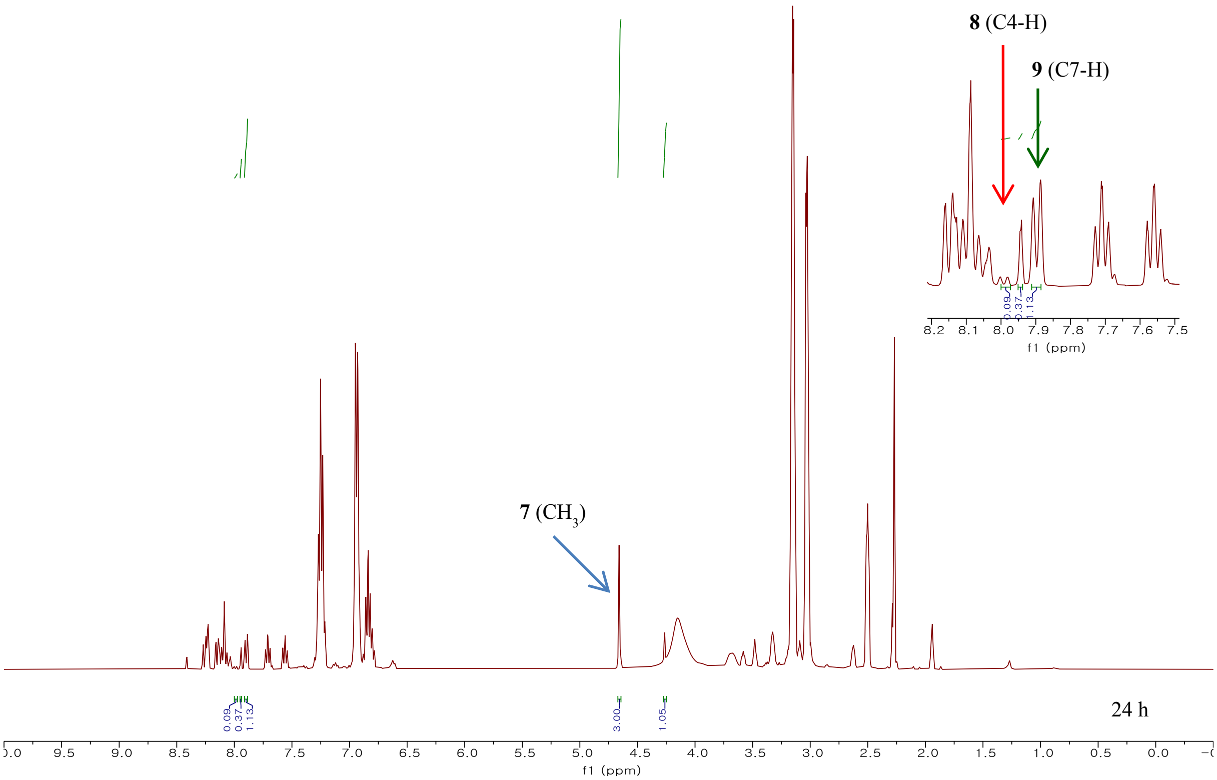

Figure S27. ^{1}H NMR experiment using precursor **7** for Table 4, entry 7 (nucleophile: 1-phenylpiperazine).

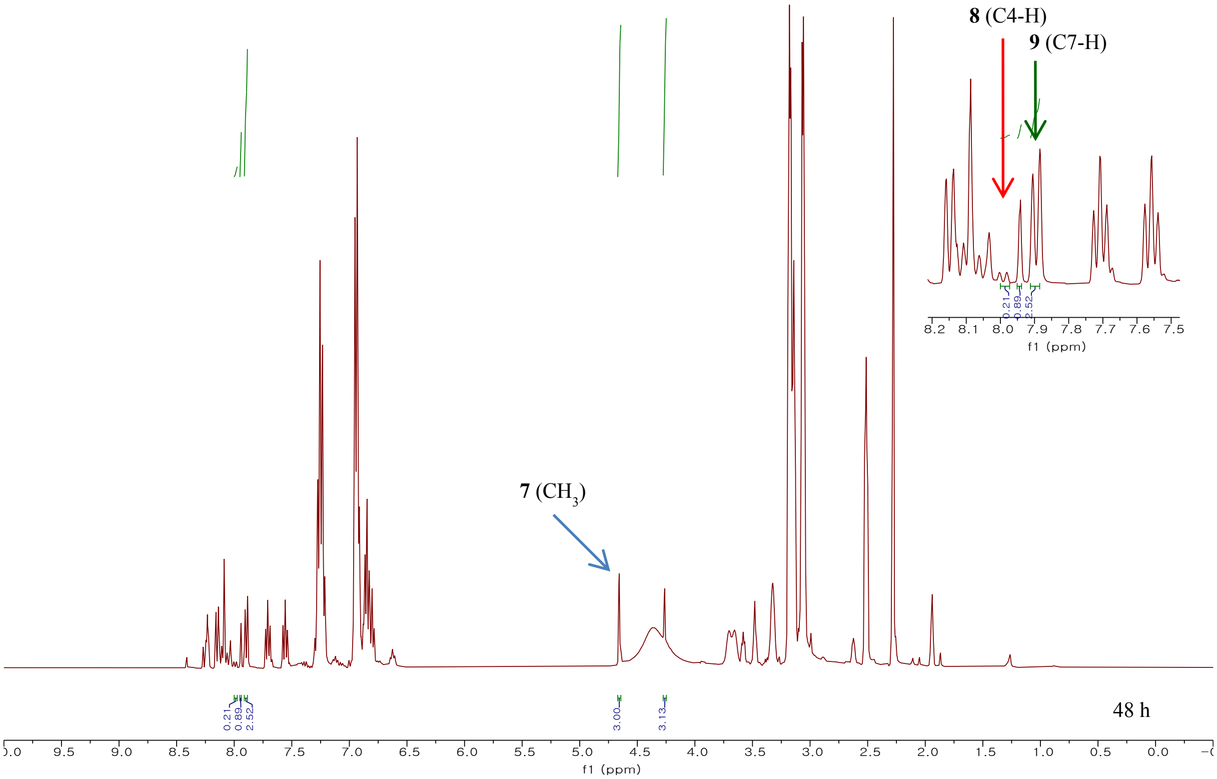

Figure S28. ^{1}H NMR experiment using precursor **7** for Table 4, entry 8 (nucleophile: 1-phenylpiperazine).

5. ^{1}H NMR experiments, ^{19}F NMR, and Table S1 for Table 5

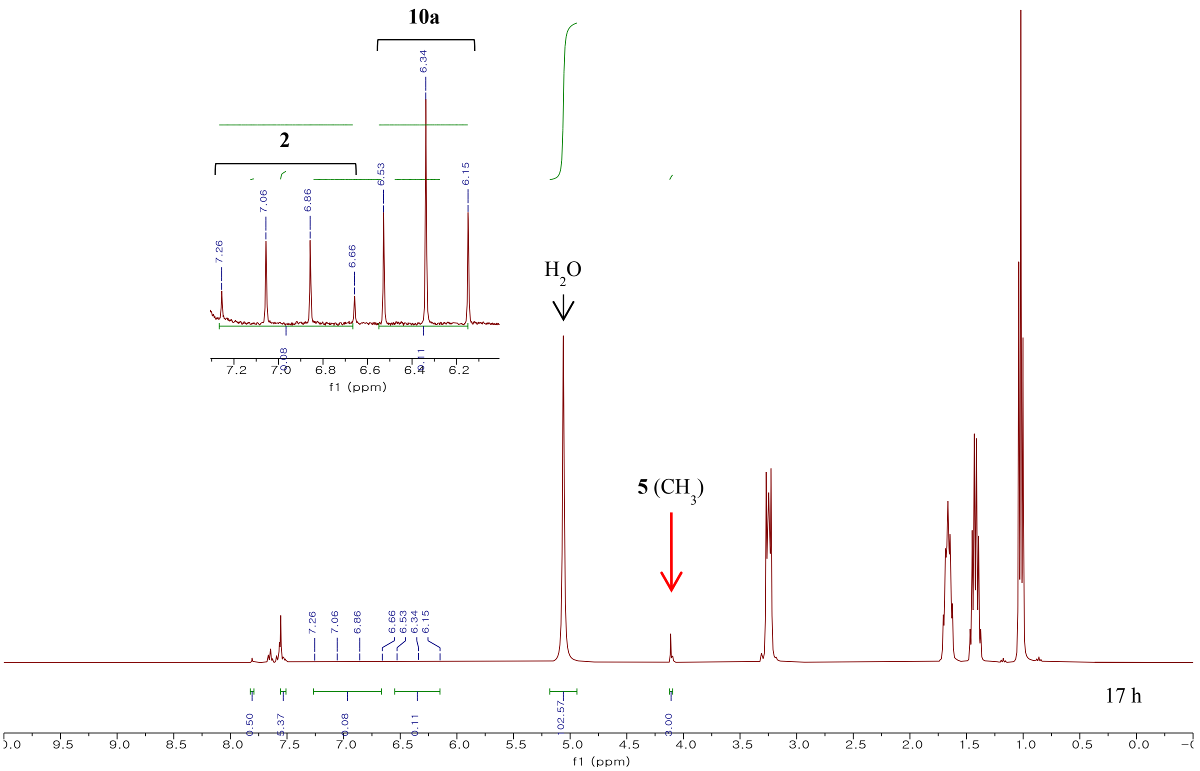

Figure S29. ^{1}H NMR experiment using precursor **1** and TBAF for Table 5, entry 1.

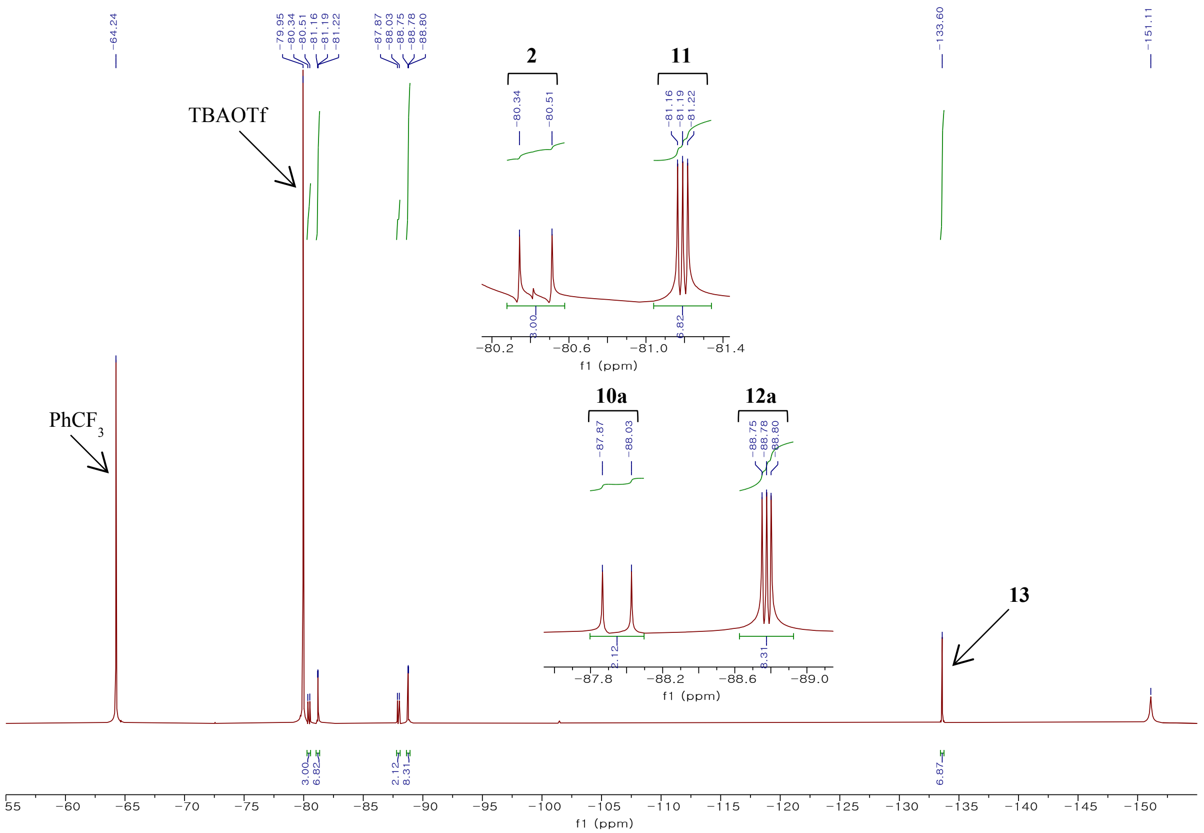

Figure S30. ^{19}F NMR spectrum of the reaction mixture (Table 5, entry 1).

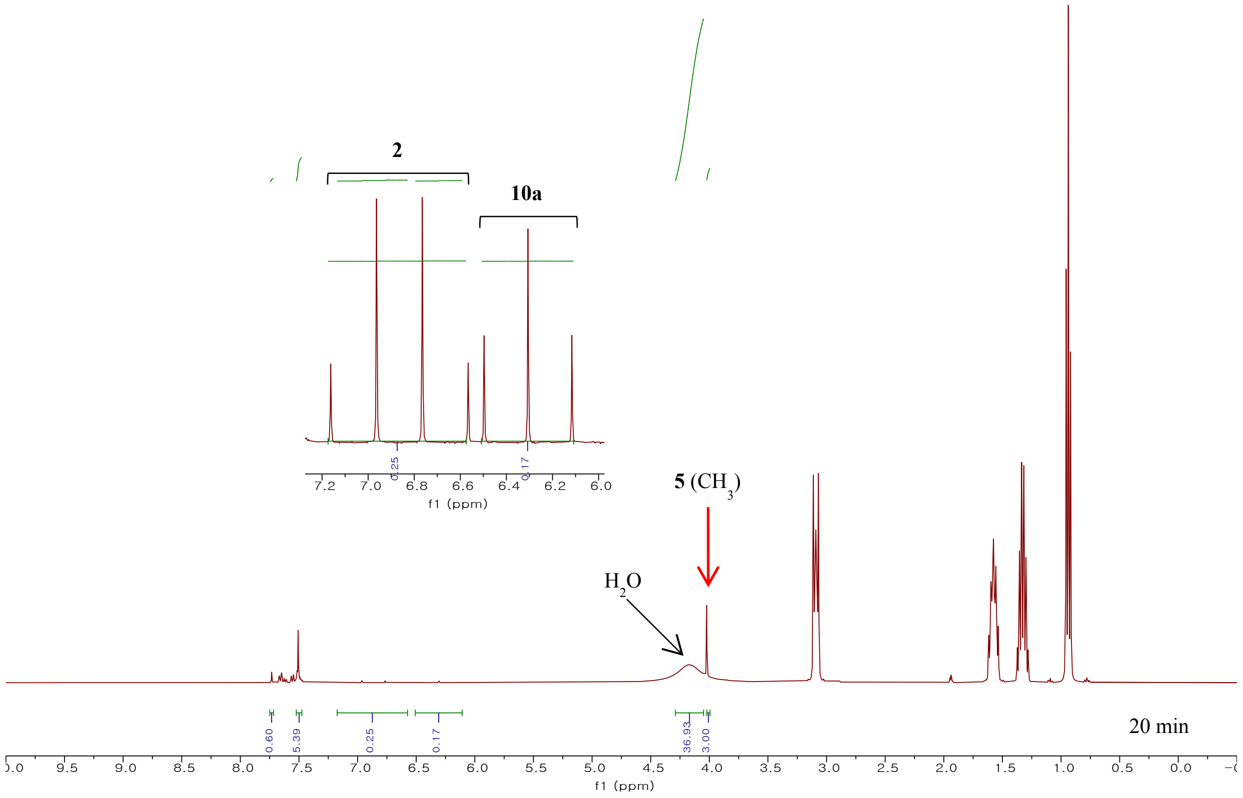

Figure S31. ^{1}H NMR experiment using precursor **1** and TBAF for Table 5, entry 2.

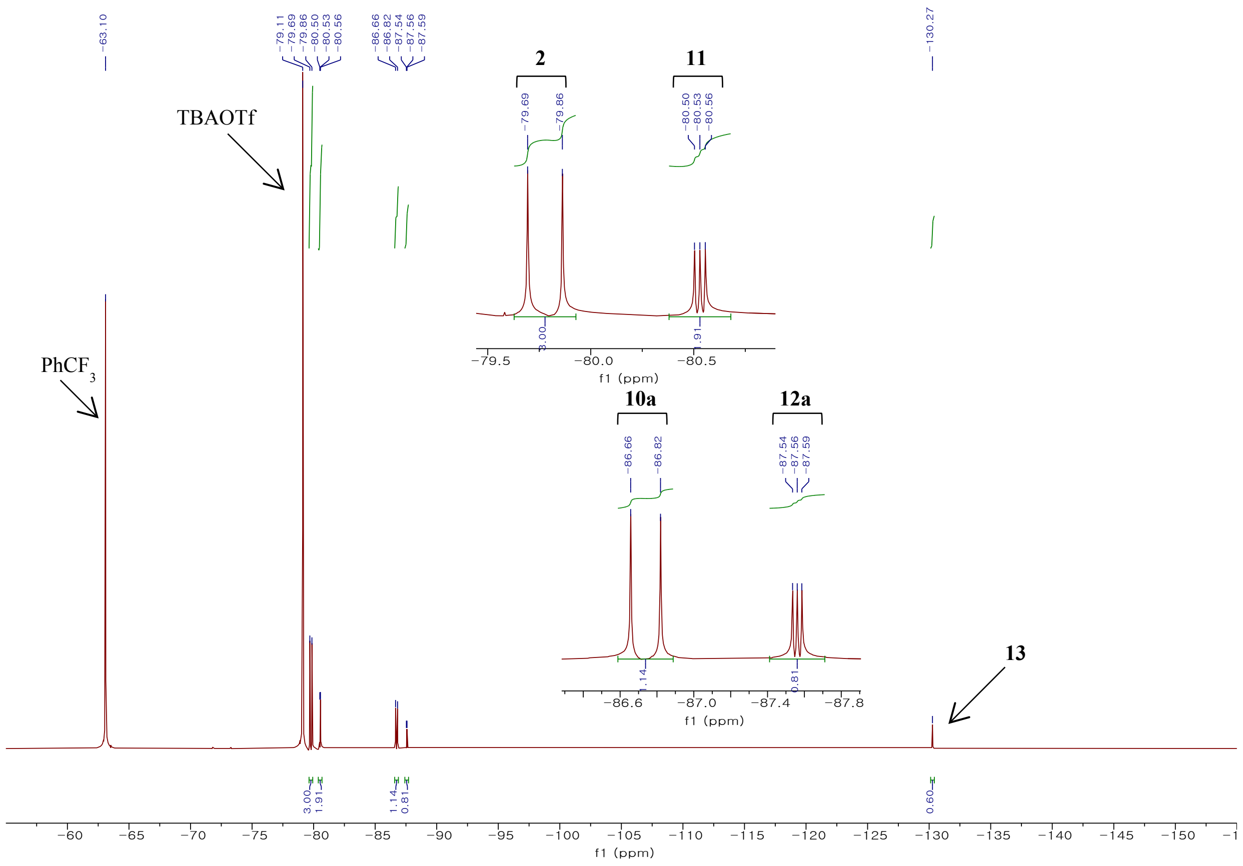

Figure S32. ^{19}F NMR spectrum of the reaction mixture (Table 5, entry 2).

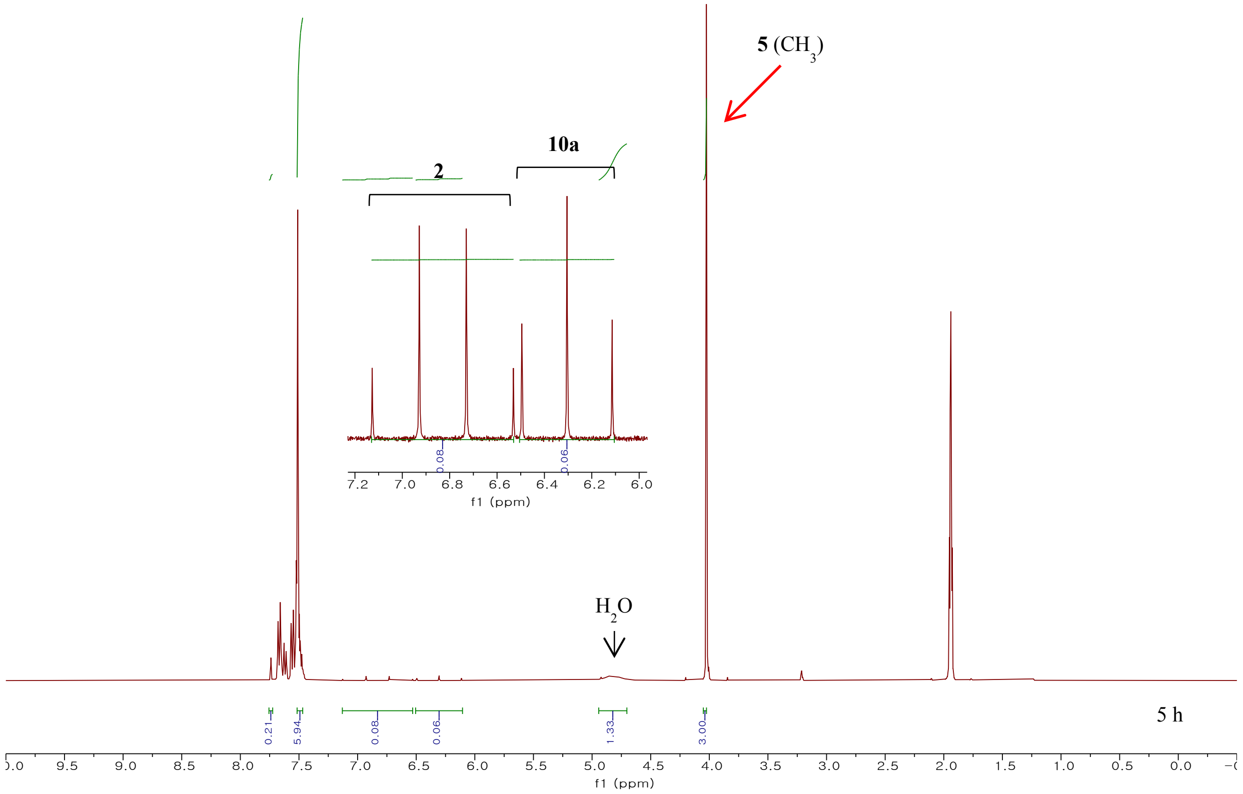

Figure S33. ^{1}H NMR experiment using precursor **1** and CsF for Table 5, entry 3.

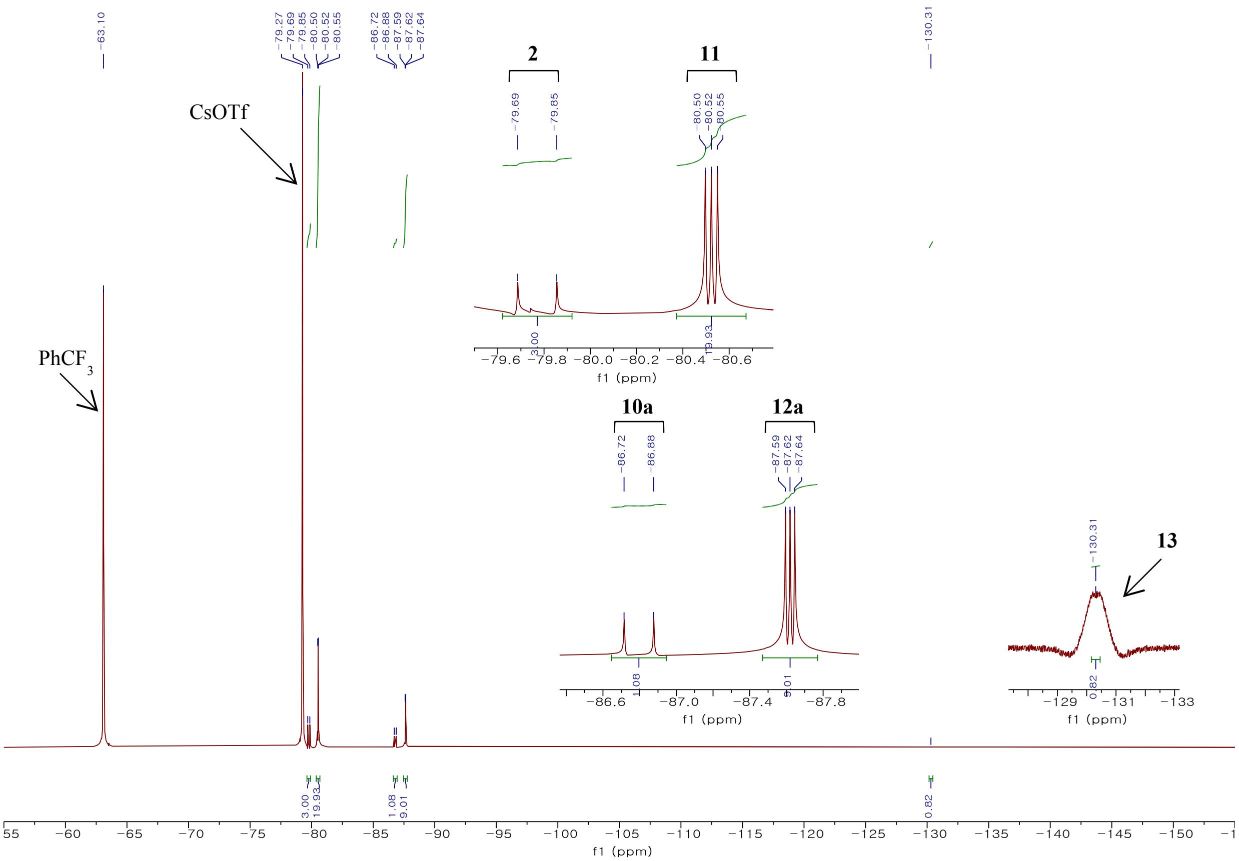

Figure S34. ^{19}F NMR spectrum of the reaction mixture (Table 5, entry 3).

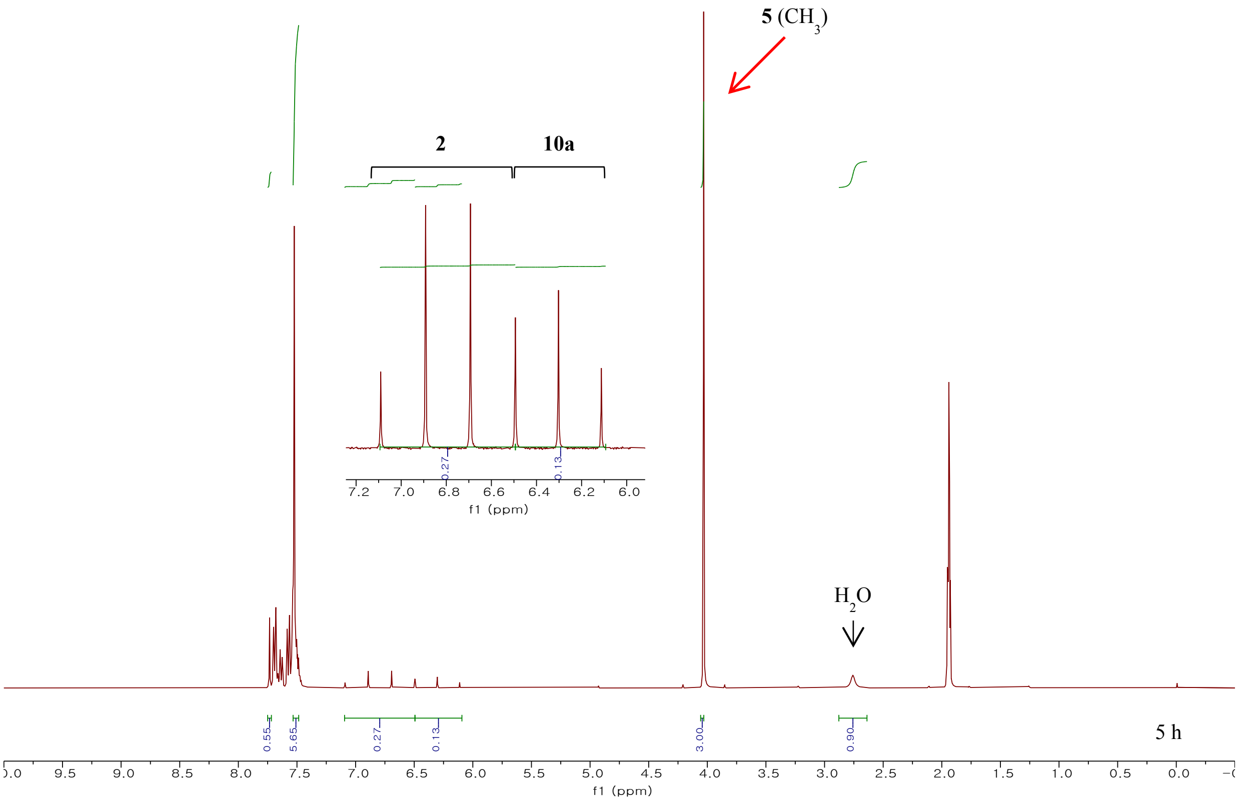

Figure S35. ^{1}H NMR experiment using precursor **1** and CsF for Table 5, entry 4.

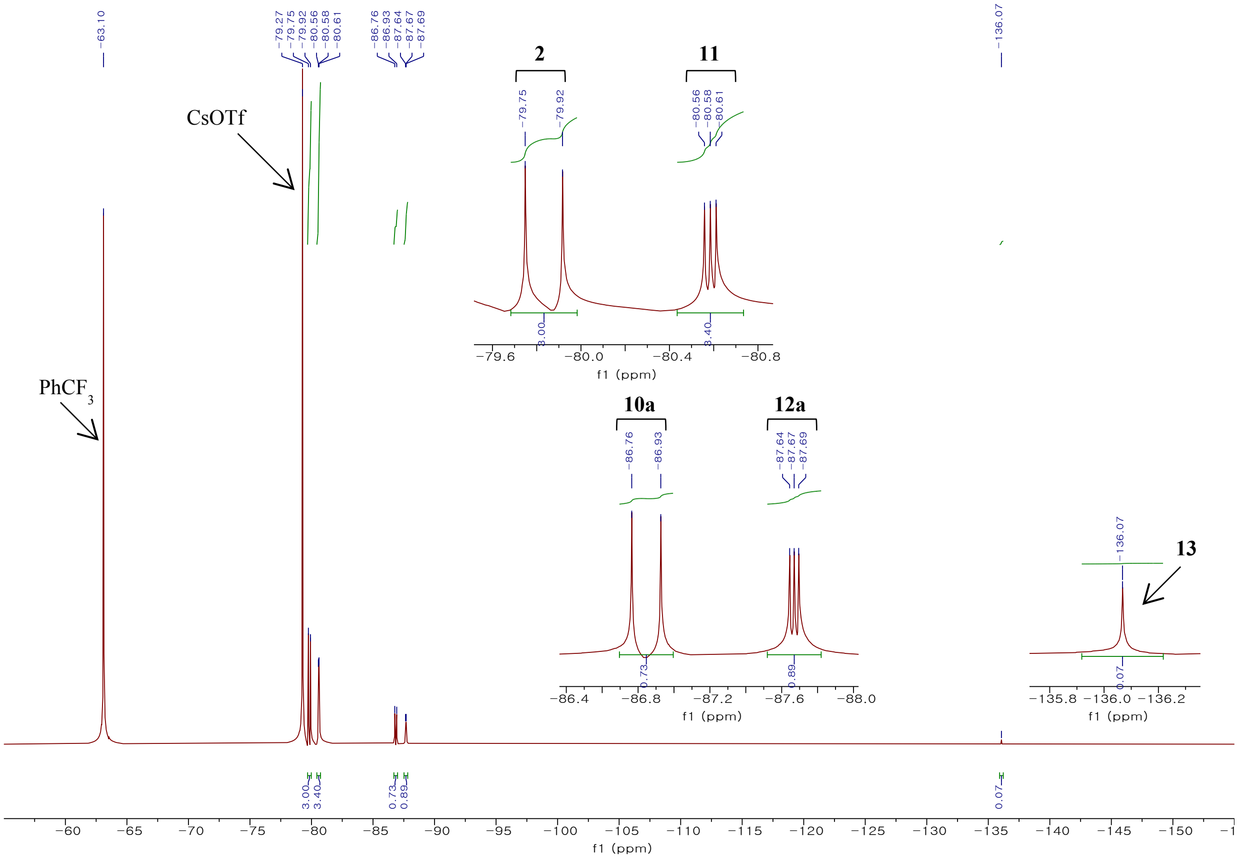

Figure S36. ^{19}F NMR spectrum of the reaction mixture (Table 5, entry 4).

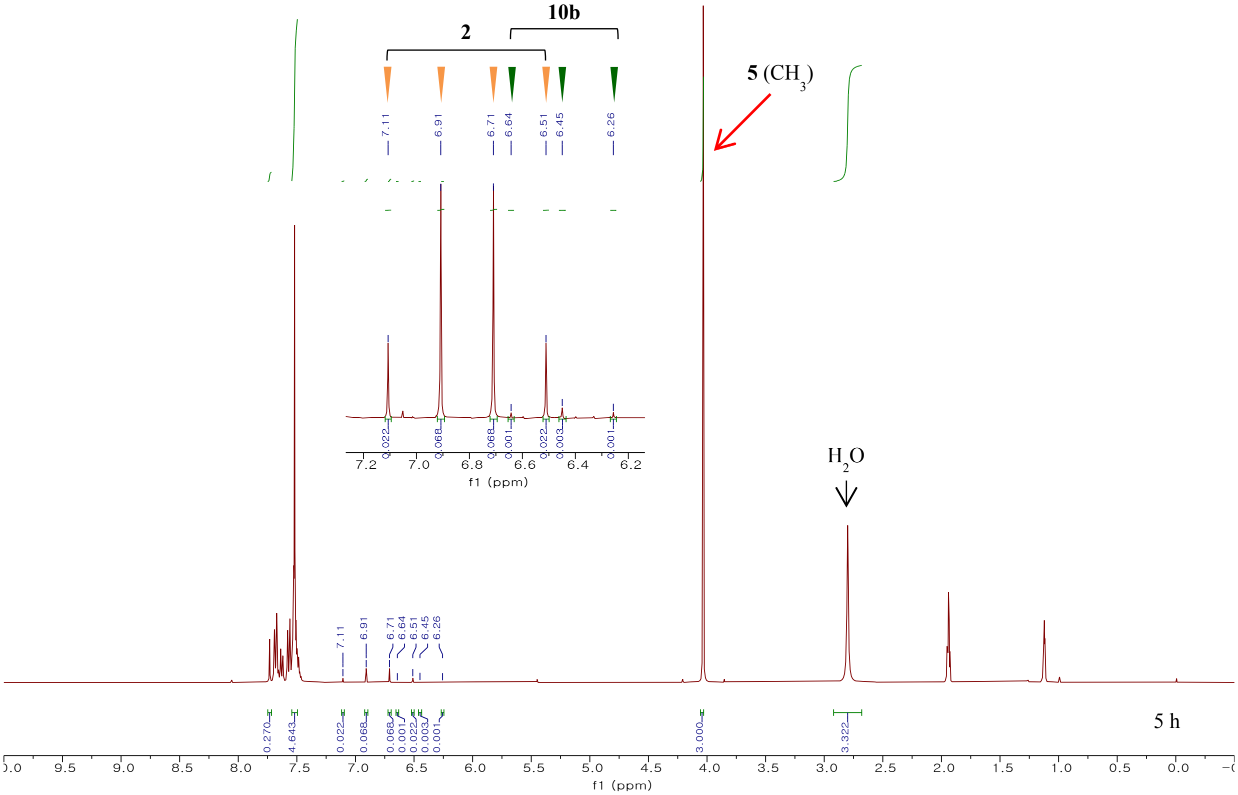

Figure S37. ^{1}H NMR experiment using precursor **1** and CsF for Table 5, entry 5.

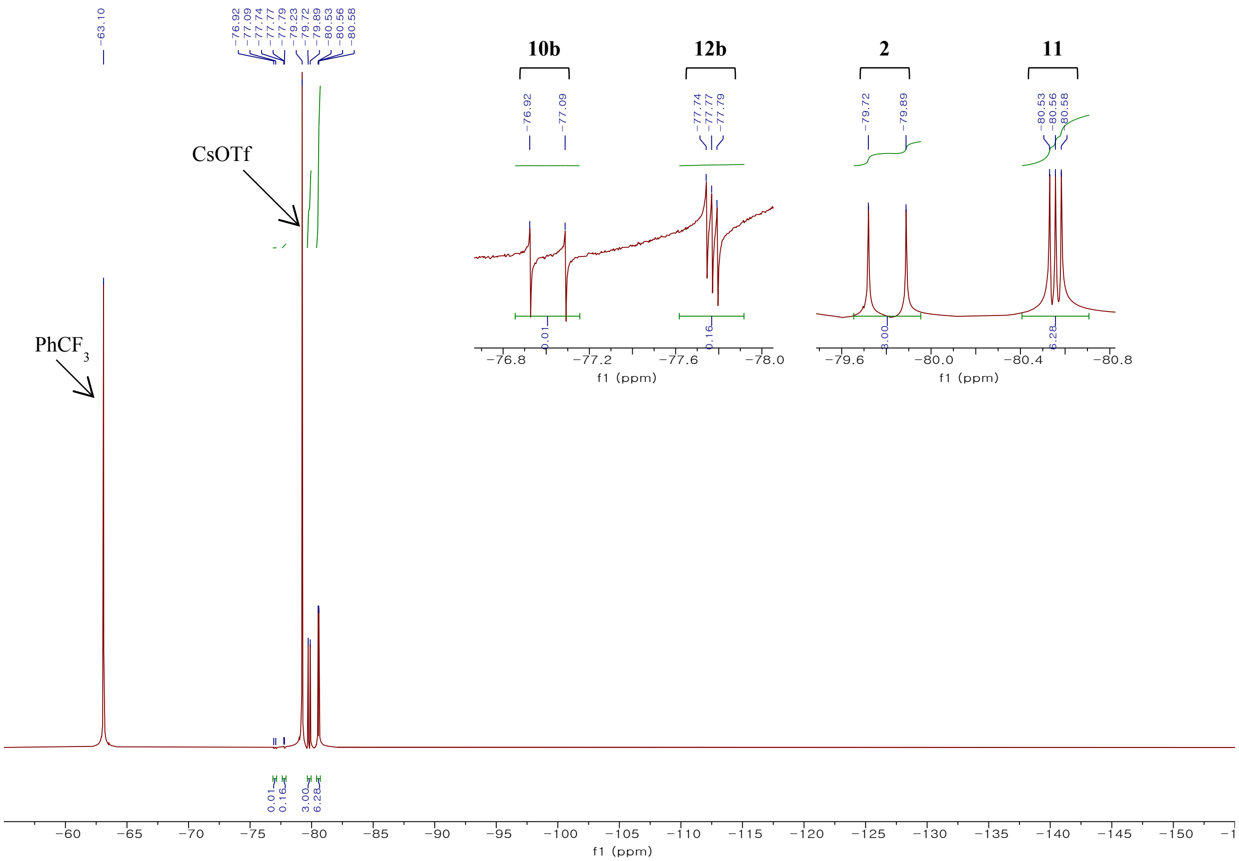

Figure S38. ^{19}F NMR spectrum of the reaction mixture (Table 5, entry 5).

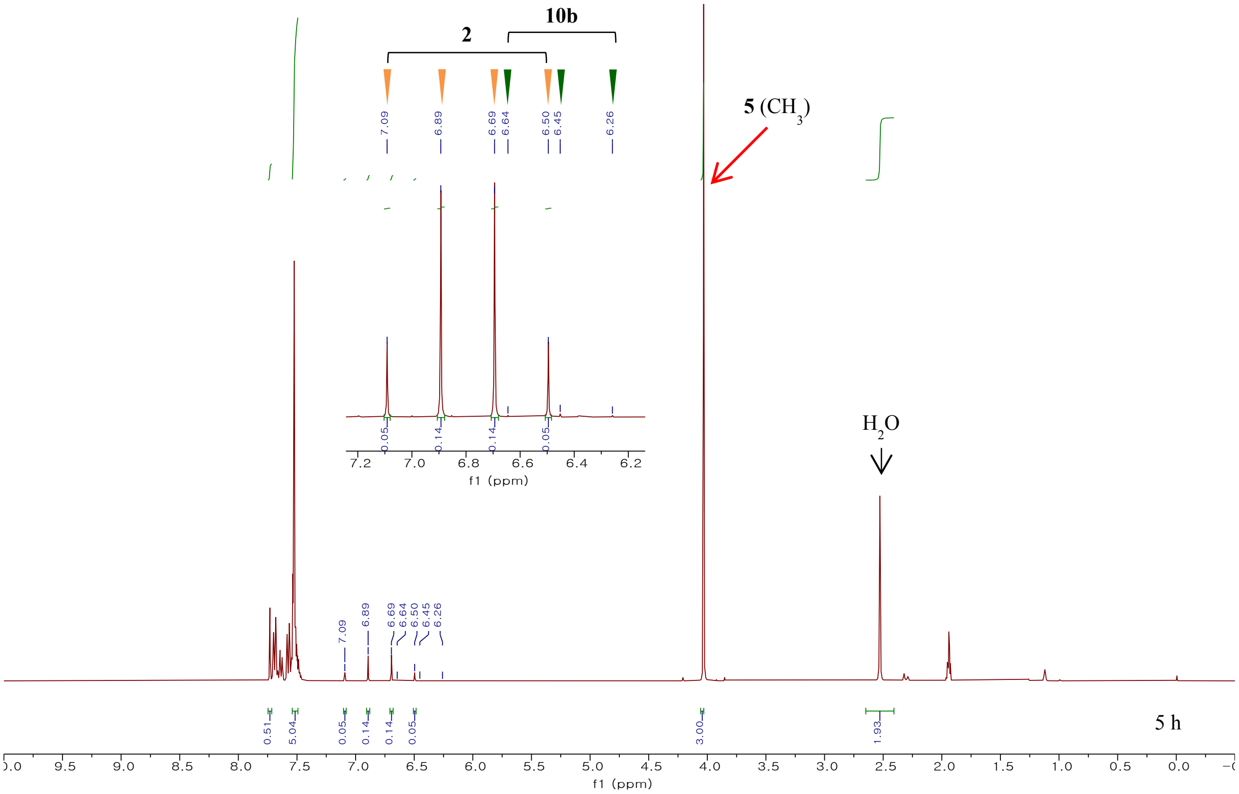

Figure S39. ^{1}H NMR experiment using precursor **1** and CsF for Table 5, entry 6.

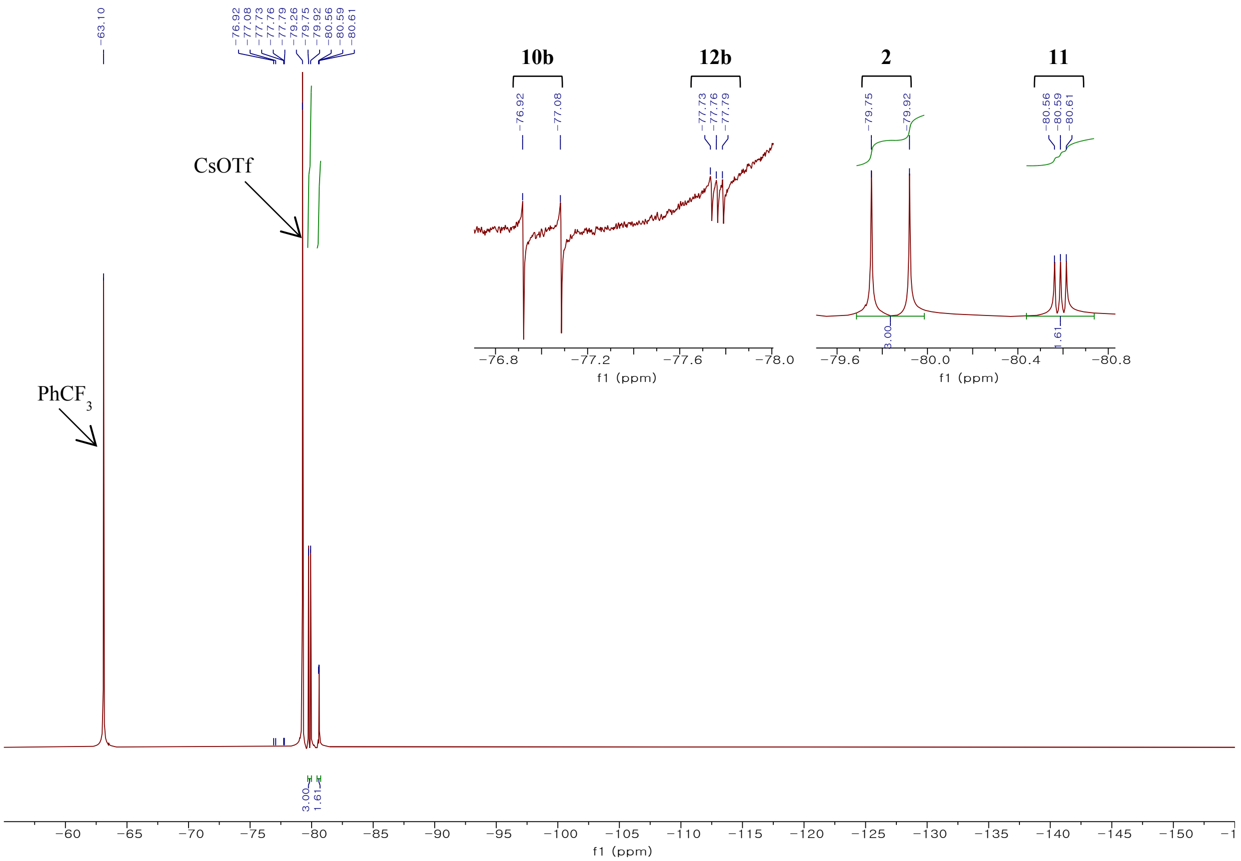

Figure S40. ^{19}F NMR spectrum of the reaction mixture (Table 5, entry 6).

Table S1. Ratio of deuterium source/H_2O in reaction mixture for Table 5[a]

entry	F^- source	solvent	deuterium source: CD_3OD (mmol)	deuterium source: $(CD_3)_3COD$ (mmol)	ratio of **5**/H_2O[b]	H_2O (mmol)[c]	ratio of deuterium source/H_2O
1	TBAF	CD_3OD	18.464	-	1 : 51.285	4.308	4.29 : 1
2	TBAF	CD_3CN/CD_3OD (6.5:1)	2.462	-	1 : 18.465	1.551	1.59 : 1
3	CsF	CD_3CN/CD_3OD (6.5:1)	2.462	-	1 : 0.665	0.056	43.96 : 1
4	CsF	CD_3CN/CD_3OD (17.8:1)	0.985	-	1 : 0.450	0.038	25.92 : 1
5	CsF	$CD_3CN/(CD_3)_3COD$ (6.5:1)	-	1.061	1 : 1.660	0.139	7.63 : 1
6	CsF	$CD_3CN/(CD_3)_3COD$ (17.8:1)	-	0.424	1 : 0.965	0.081	5.23 : 1

[a]All reactions were carried out on a 0.084 mmol reaction scale of **1** in 0.75 mL of solvent in a sealed NMR tube. [b]Ratio was determined by ^{1}H NMR. [c]Complete conversion of **1** into **5** was observed in ^{1}H NMR.

6. Determination of modified values for Table 6

Table S2. Factors for equations S1–S4

entry	F^- source	ratio of deuterium source/H_2O	^{19}F NMR yield				modified value			
			**2**	**11**	**10a**	**12a**	**2**	**11**	**10a**	**12a**
1	TBAF	$x_d : x_w$	f_2	f_{11}	f_{10a}	f_{12a}	y_2	z_{11}	y_{10a}	z_{12a}

The ratio of deuterium source and H_2O was indicated in Table S1. ^{19}F NMR yields of products were indicated in Table 5. Modified values in Table 6 were calculated from equations S1–S4.

z_{11} and y_2 were calculated from equations S1 and S2, respectively.

z_{11} = yield of **14** via route (c) (= yield of **11** from **14** if reaction mixture contains only deuterium source and no H_2O)

y_2 = yield of **2** via only route (a)

$z_{11}(x_d/(x_d + x_w)) = f_{11}$ (eqn. S1)

$y_2 + (x_w/(x_d + x_w))z_{11} = f_2$ (eqn. S2)

z_{12a} and y_{10a} were calculated from equations S3 and S4, respectively.

z_{12a} = yield of **15a** via route (c) (= yield of **12a** from **15a** if reaction mixture contains only deuterium source and no H_2O)

y_{10a} = yield of **10a** via only route (a)

$z_{12a}(x_d/(x_d + x_w)) = f_{12a}$ (eqn. S3)

$y_{10a} + (x_w/(x_d + x_w))z_{12a} = f_{10a}$ (eqn. S4)

7. NMR spectra of compounds 1, 5, 7, 8

^1H, ^{13}C and ^{19}F NMR spectra of **1** (400, 100, 470 MHz, CD_3CN)

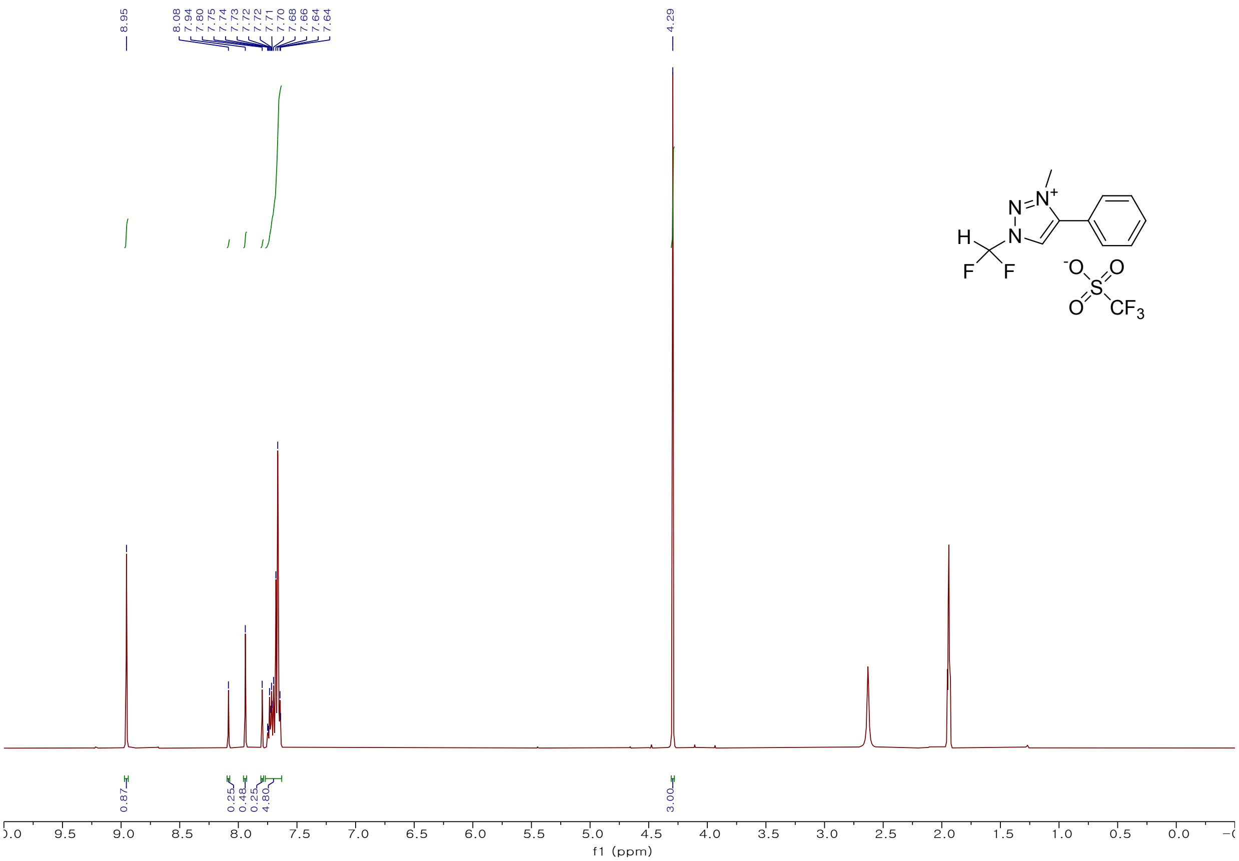
8.95
8.08
7.94
7.80
7.75
7.74
7.73
7.72
7.72
7.71
7.70
7.68
7.66
7.64
7.64
4.29
0.87
0.25
0.48
0.25
4.80
3.00
f1 (ppm)

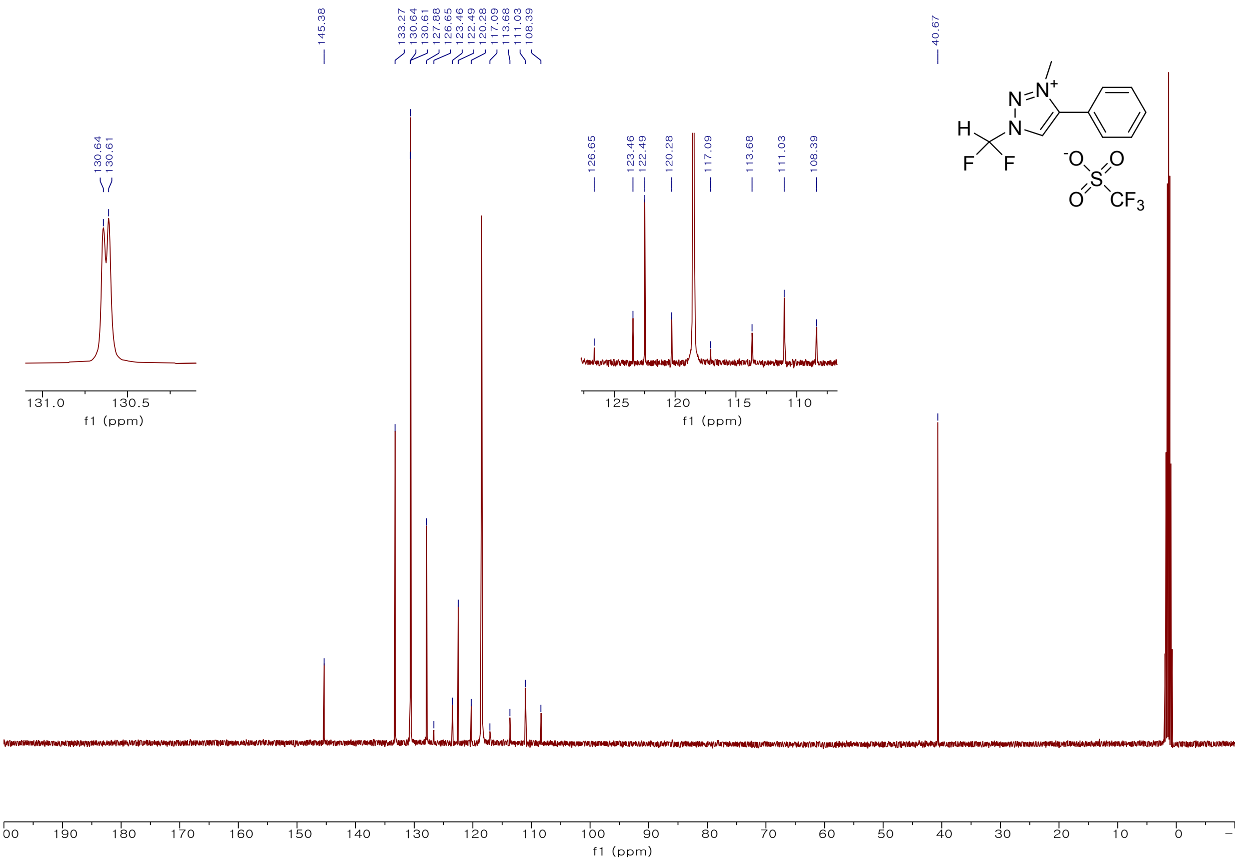
145.38
133.27
130.64
130.61
127.88
126.65
123.46
122.49
120.28
117.09
113.68
111.03
108.39
40.67
f1 (ppm)

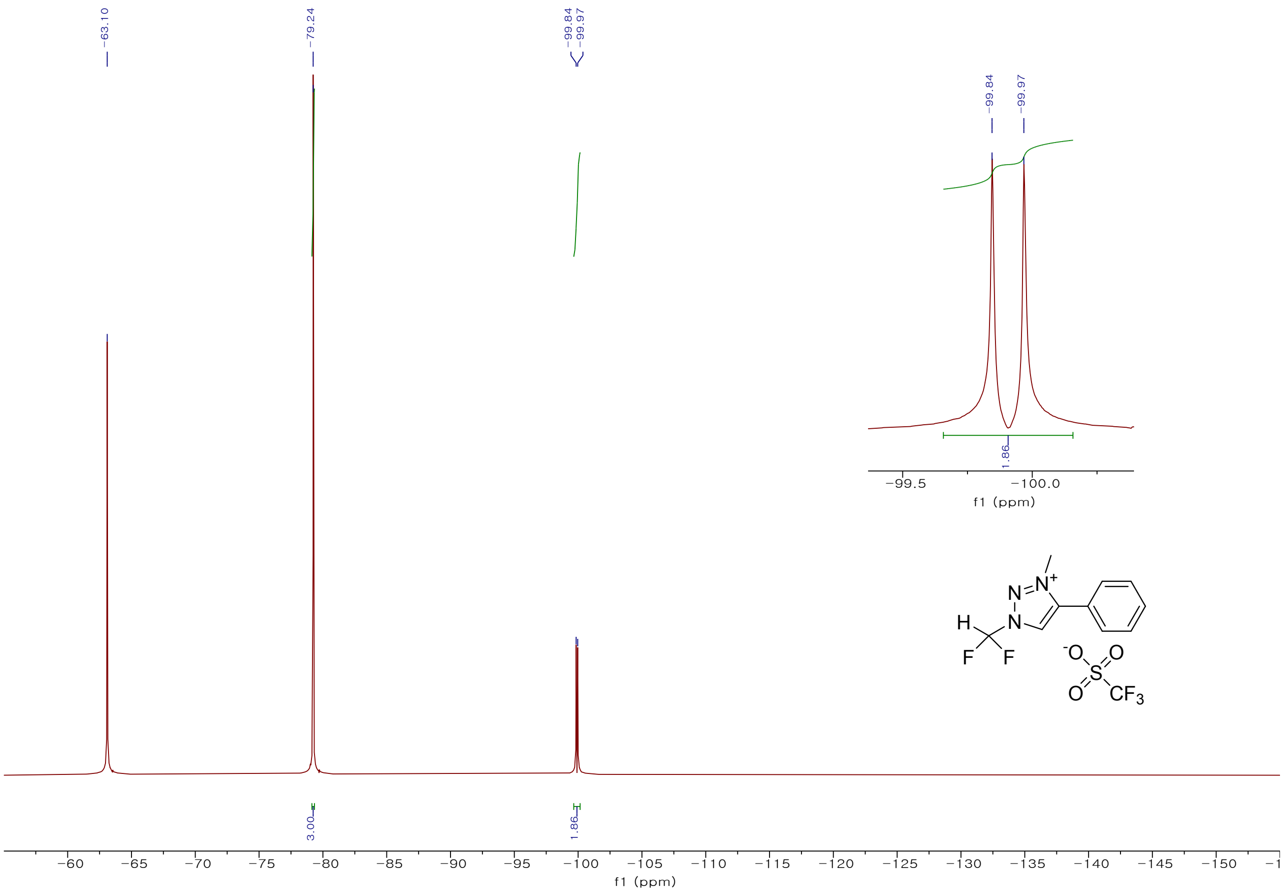

^1H and ^{13}C NMR spectra of **5** (400, 100 MHz, $CDCl_3$)

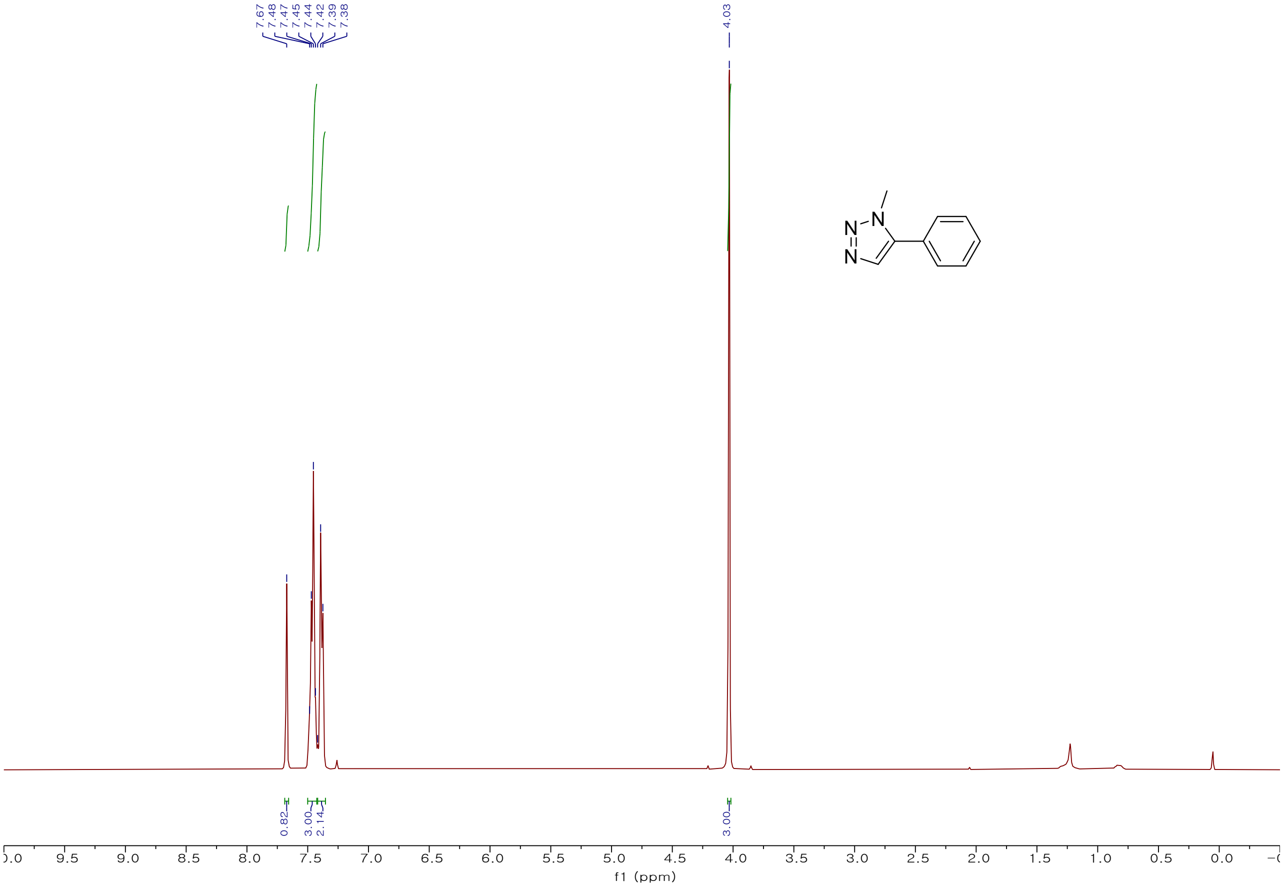

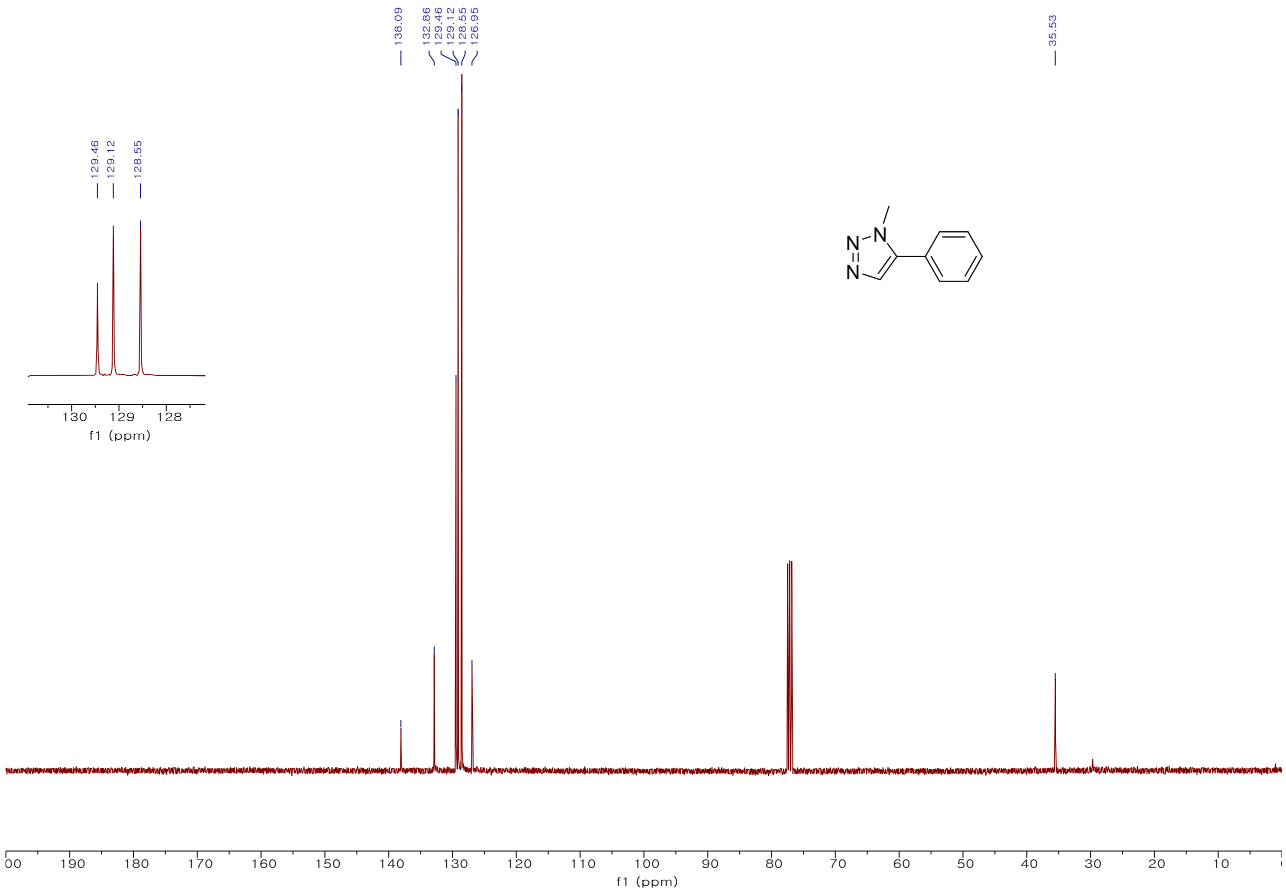

^{1}H, ^{13}C and ^{19}F NMR spectra of **7** (400, 100, 470 MHz, CD_3CN)

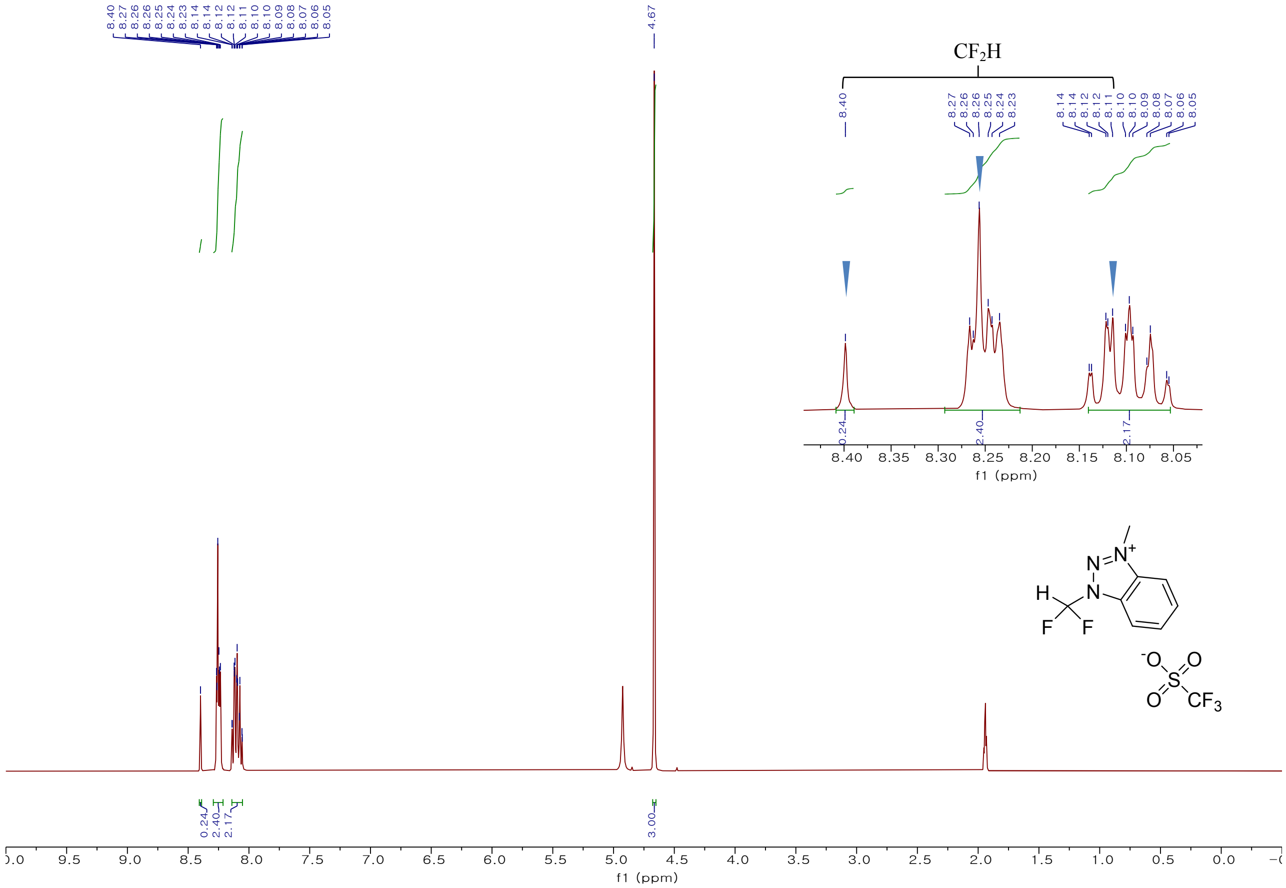

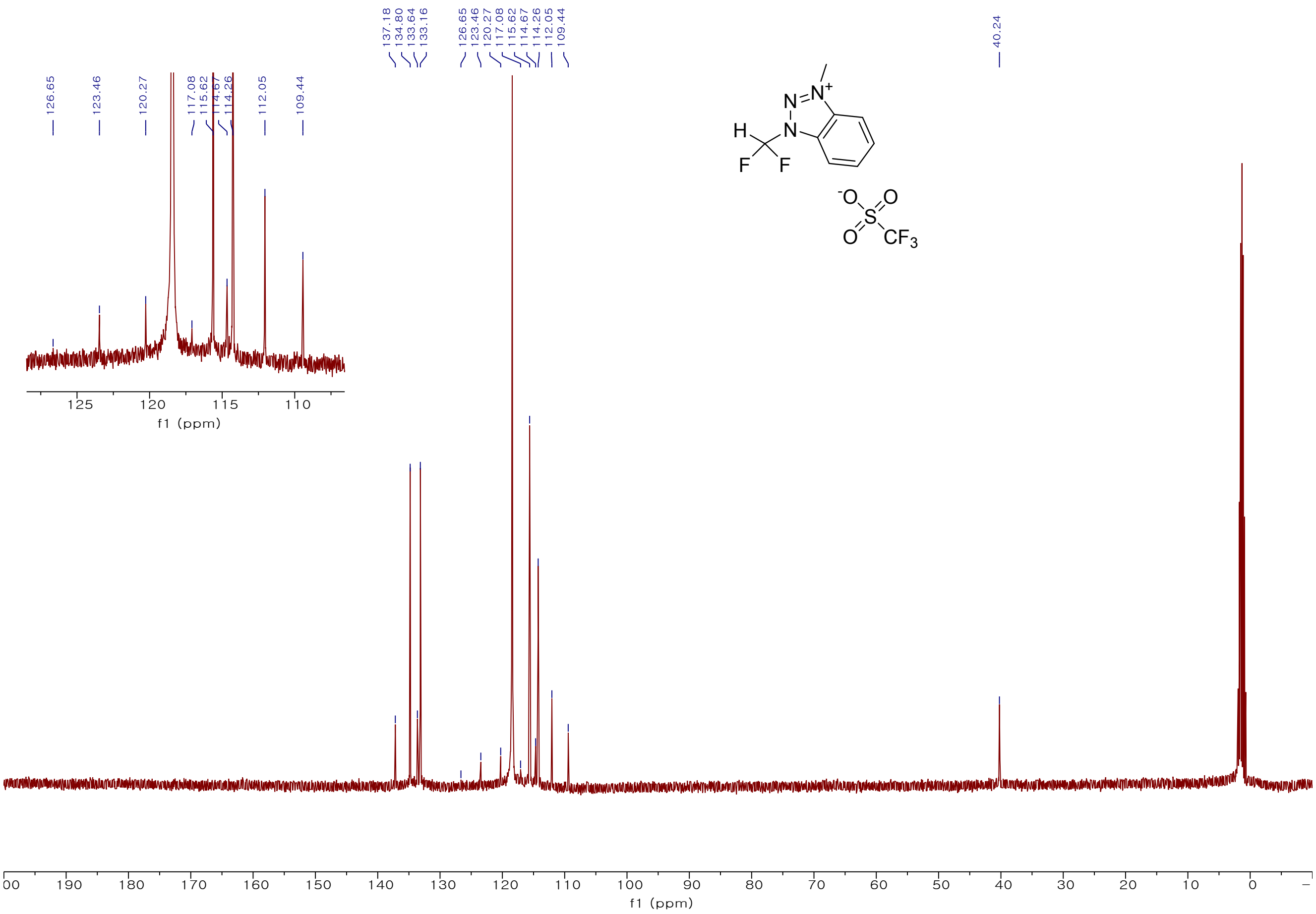
137.18
134.80
133.64
133.16
126.65
123.46
120.27
117.08
115.62
114.67
114.26
112.05
109.44
40.24
f1 (ppm)

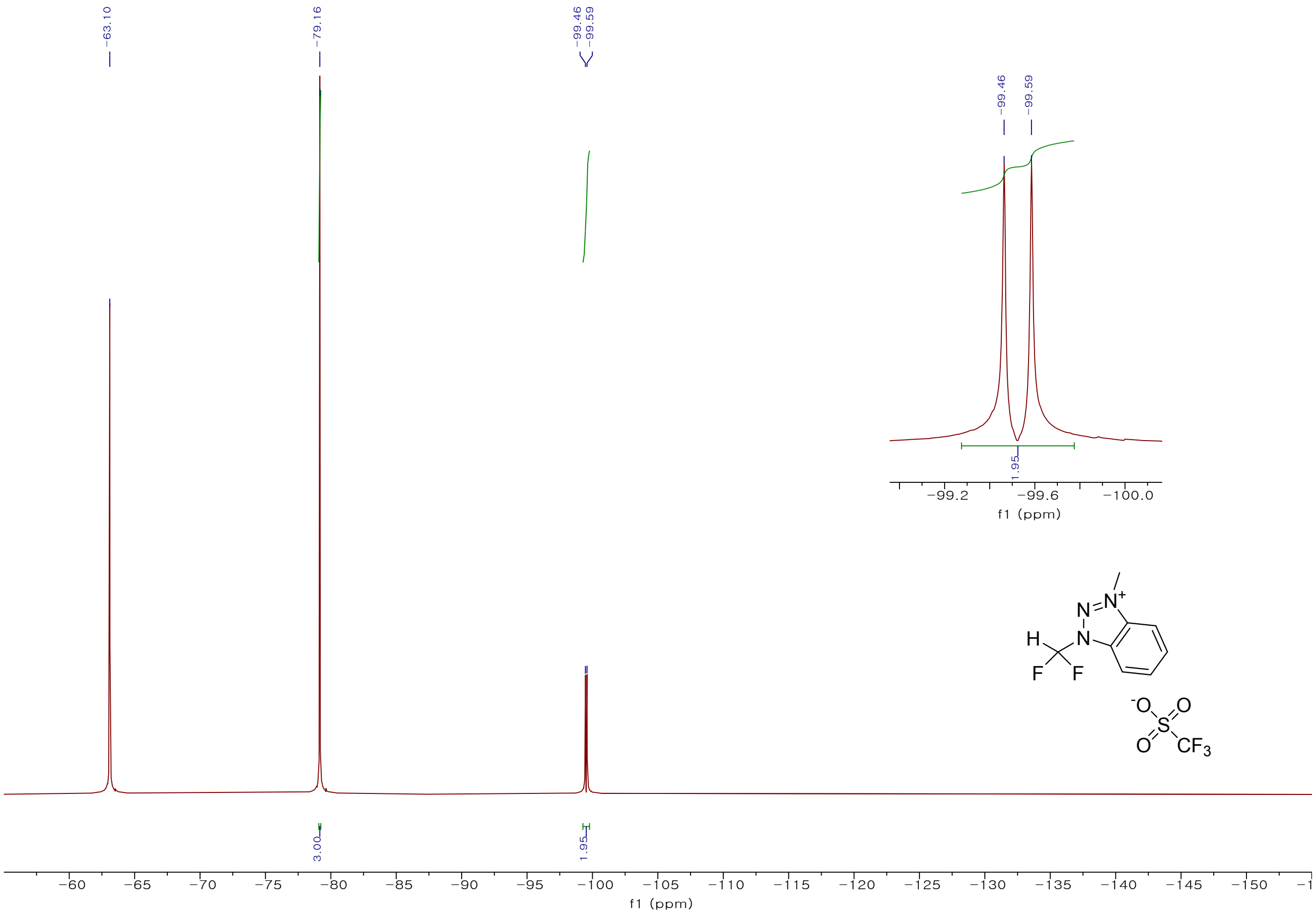
-63.10
-79.16
-99.46
-99.59
3.00
1.95
f1 (ppm)

^{1}H and ^{13}C NMR spectra of **8** (400, 100 MHz, $CDCl_3$)

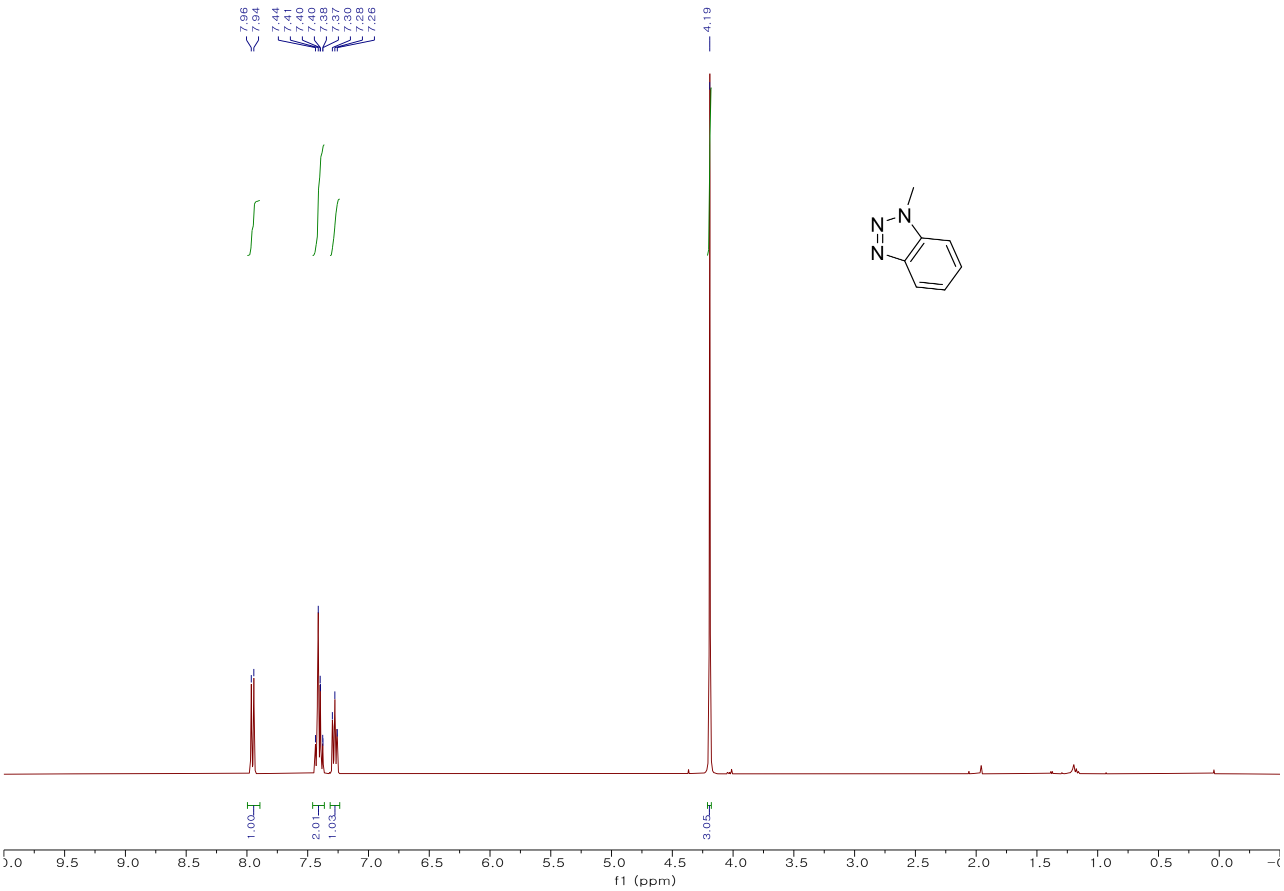

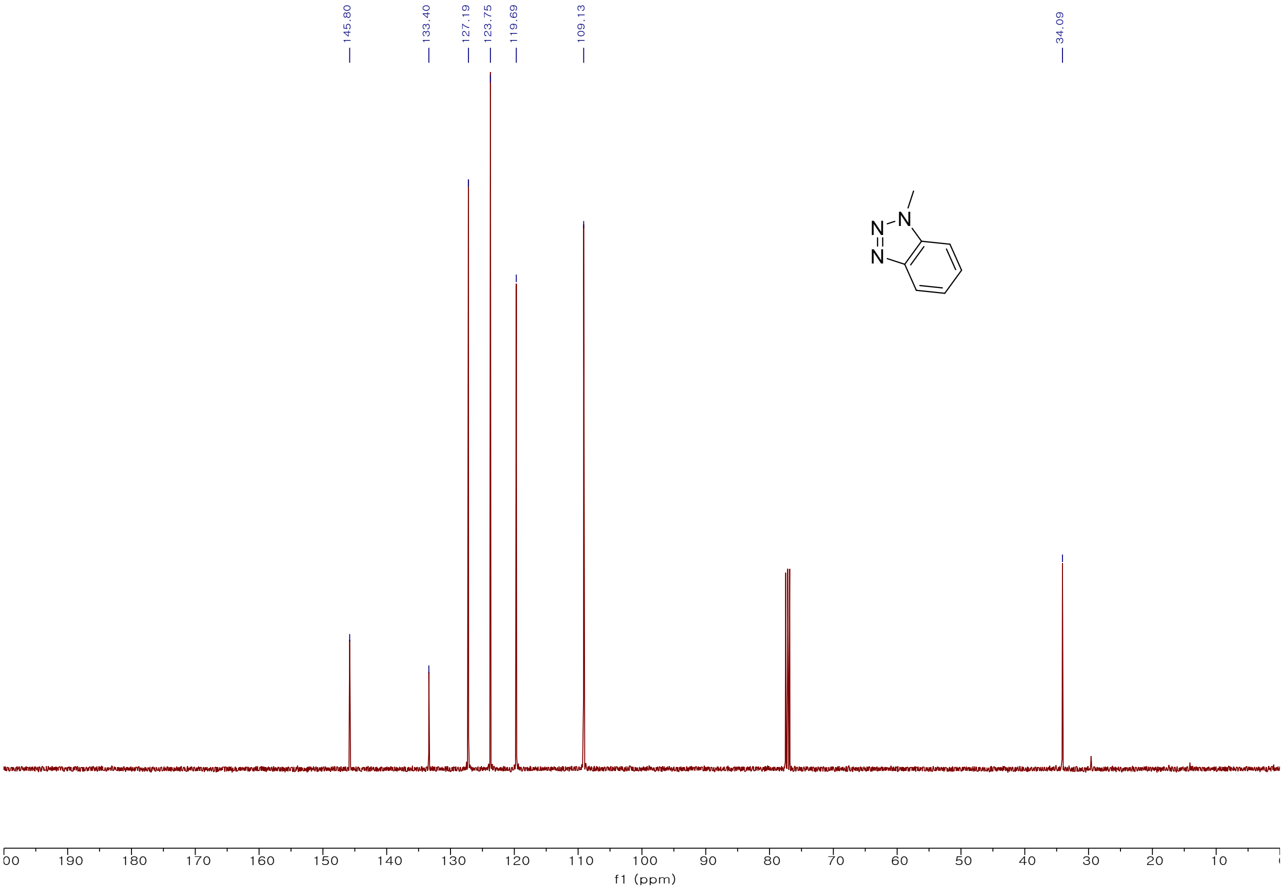

8. Cartesian coordinates

$(CsF\text{-}1)_{Pre}$

C	1.338702	1.729334	-0.845771
C	2.193930	1.290461	0.134443
N	0.361971	2.069046	1.113382
H	1.415597	1.723610	-1.921106
N	0.256186	2.190257	-0.181648
N	1.530134	1.539612	1.304570
C	-1.002747	2.694170	-0.721261
H	-1.816917	2.586250	0.020000
C	1.985764	1.301308	2.675722
H	1.409516	1.958476	3.322193
H	1.803630	0.258475	2.925646
H	3.045288	1.542349	2.729133
F	-1.249689	2.007514	-1.850397
F	-0.812711	3.982581	-1.080584
C	3.528164	0.687413	0.025408
C	3.860049	-0.455243	0.758339
C	4.451511	1.248136	-0.860765
C	5.120473	-1.022651	0.610930
H	3.124403	-0.913753	1.408777
C	5.708023	0.671222	-1.004775
H	4.186556	2.136122	-1.424366
C	6.044309	-0.461276	-0.267394
H	5.376614	-1.911297	1.175926
H	6.424743	1.108091	-1.690243
H	7.025126	-0.909034	-0.380569
S	-0.238843	-1.577530	0.569535
O	0.812375	-1.367796	1.565568
O	-1.296450	-2.515316	0.945783
O	-0.723988	-0.354913	-0.081056
C	0.655197	-2.439560	-0.803262

F	1.211900	-3.570632	-0.371194
F	-0.181556	-2.741634	-1.796493
F	1.620652	-1.662750	-1.294701
Cs	-3.881008	-0.424567	-0.005523
F	-3.188773	2.302690	1.029657

$(CsF\text{-}2)_{Pre}$

C	-0.809326	2.453134	-0.567335
C	0.328154	2.274994	0.184254
N	-1.424173	1.971425	1.506351
H	-0.953030	2.670797	-1.612592
N	-1.826530	2.256119	0.297778
N	-0.128968	1.991885	1.439648
C	-3.269002	2.281093	0.039470
H	-3.717187	1.311411	0.237243
C	0.643470	1.720671	2.655007
H	-0.037044	1.845162	3.493714
H	1.051198	0.705873	2.615557
H	1.450605	2.450335	2.708824
F	-3.426910	2.654160	-1.230010
F	-3.804142	3.225589	0.829653
C	1.734663	2.328924	-0.230009
C	2.675609	1.429837	0.282014
C	2.111716	3.265029	-1.198380
C	3.988304	1.479863	-0.177466
H	2.405537	0.681045	1.024646
C	3.424519	3.303545	-1.650618
H	1.380298	3.964884	-1.587707
C	4.365610	2.411791	-1.139826
H	4.715724	0.780108	0.219056
H	3.714022	4.032644	-2.398291
H	5.390076	2.444636	-1.492587
S	-1.524630	-1.048362	-0.822570

O	-0.676122	-0.553113	0.268118
O	-2.472207	-0.069995	-1.358917
O	-0.816539	-1.860259	-1.812744
C	-2.617105	-2.270139	0.034735
F	-3.370537	-1.662974	0.951658
F	-3.426079	-2.878887	-0.830921
F	-1.884007	-3.200959	0.646185
Cs	2.005628	-2.322721	-0.077779
F	2.423624	-0.818172	2.457851

$(PZ\text{-}1)_{Pre}$

C	2.561387	1.397630	-0.597789
C	3.447563	0.856489	0.298723
N	1.680040	1.568617	1.430030
H	2.593640	1.495486	-1.670682
N	1.512440	1.800458	0.156851
N	2.845596	1.010411	1.514736
C	0.273659	2.453150	-0.267768
H	-0.540768	2.189219	0.396119
C	3.320605	0.556961	2.821336
H	2.808615	1.147883	3.576547
H	3.077370	-0.499286	2.929750
H	4.395485	0.716395	2.870419
F	0.046170	2.066365	-1.525030
F	0.483756	3.783124	-0.281104
C	4.738682	0.188797	0.084186
C	4.944053	-1.101760	0.577619
C	5.739876	0.834145	-0.643803
C	6.159037	-1.738059	0.351760
H	4.150023	-1.610635	1.112686
C	6.951321	0.189142	-0.867597
H	5.570070	1.836329	-1.021513
C	7.162345	-1.093297	-0.367823

H	6.317962	-2.740612	0.731092
H	7.731585	0.690642	-1.427993
H	8.108500	-1.592711	-0.542283
S	-0.011057	-1.367437	0.430292
O	-0.762349	-0.108134	0.495560
O	1.413959	-1.243288	0.754458
O	-0.682329	-2.532023	0.999385
C	0.020139	-1.723766	-1.387580
F	0.659625	-0.754068	-2.045252
F	0.651050	-2.873395	-1.636651
F	-1.213366	-1.814598	-1.885727
C	-3.285608	2.292507	0.886168
C	-2.984388	1.751802	-1.453925
C	-4.471733	1.403618	-1.539706
C	-4.768797	1.930625	0.778179
H	-2.633650	2.155268	-2.406815
H	-2.723755	1.411162	1.217504
H	-3.146824	3.084452	1.626632
H	-5.034537	2.314450	-1.772099
H	-4.680604	0.687442	-2.333564
H	-5.339307	2.817287	0.481946
H	-5.156297	1.599479	1.738725
H	-2.400678	0.852061	-1.229673
N	-2.712363	2.731565	-0.392825
H	-3.146601	3.615476	-0.652124
N	-4.976709	0.909616	-0.256689
C	-4.788439	-0.437599	0.074426
C	-4.258994	-1.369543	-0.839574
C	-5.194858	-0.930920	1.332052
C	-4.167695	-2.720077	-0.516037
H	-3.899954	-1.050867	-1.808109
C	-5.089920	-2.279382	1.643710
H	-5.622793	-0.263798	2.068433

C	-4.580380	-3.194317	0.723811
H	-3.751220	-3.404552	-1.247779
H	-5.421806	-2.617460	2.619732
H	-4.500043	-4.245862	0.972005

$(PZ\text{-}1)_{TS}$

C	-2.576253	-1.363378	-0.632560
C	-3.476067	-0.808225	0.250732
N	-1.836164	-1.766762	1.380972
H	-2.565870	-1.365798	-1.710013
N	-1.606288	-1.927538	0.114843
N	-2.966045	-1.103010	1.473587
C	0.149223	-2.435225	-0.373027
H	0.485618	-1.497786	0.048656
C	-3.457644	-0.708598	2.787581
H	-3.117592	-1.452370	3.504576
H	-3.051755	0.271799	3.036424
H	-4.544957	-0.679029	2.763741
F	-0.170243	-2.586357	-1.640703
F	0.138394	-3.548615	0.323917
C	-4.680017	0.003162	0.021629
C	-4.797314	1.256195	0.629480
C	-5.684889	-0.456221	-0.832029
C	-5.924186	2.035902	0.394865
H	-3.995774	1.622650	1.261242
C	-6.807782	0.331029	-1.066240
H	-5.587733	-1.429658	-1.300223
C	-6.930080	1.574210	-0.450915
H	-6.011940	3.008410	0.865264
H	-7.589138	-0.029089	-1.725338
H	-7.807027	2.185119	-0.632132
S	-0.010708	1.424176	0.752423
O	0.867338	0.256312	0.921898

O	-1.404172	1.203154	1.139433
O	0.566378	2.698922	1.167667
C	-0.101978	1.572460	-1.094051
F	-0.411794	0.394697	-1.647469
F	-1.029232	2.457207	-1.459348
F	1.067098	1.965333	-1.600984
C	2.888069	-2.621123	0.456365
C	2.506675	-1.544924	-1.709226
C	4.021234	-1.479174	-1.900504
C	4.393157	-2.527765	0.206329
H	1.998827	-1.687413	-2.664237
H	2.553351	-1.743715	1.022309
H	2.645113	-3.519634	1.025241
H	4.365167	-2.408972	-2.362566
H	4.296048	-0.667575	-2.571714
H	4.728375	-3.412766	-0.342018
H	4.930460	-2.509564	1.150719
H	2.147148	-0.615583	-1.253060
N	2.127800	-2.646533	-0.805831
H	2.267995	-3.542457	-1.275289
N	4.709315	-1.354109	-0.615450
C	4.718978	-0.095114	0.013163
C	4.304803	1.074711	-0.649409
C	5.197379	0.044252	1.330230
C	4.367191	2.315155	-0.020921
H	3.914337	1.035080	-1.656805
C	5.250674	1.287543	1.946666
H	5.546962	-0.817804	1.882196
C	4.835992	2.439140	1.281686
H	4.030881	3.191727	-0.564562
H	5.625522	1.351722	2.962560
H	4.874041	3.405907	1.769123

(PZ-1)$_{Post}$

C	2.647188	2.063320	0.032746
C	3.467591	1.113914	0.609891
N	2.315661	2.185913	2.161234
H	2.512289	2.298508	-1.011099
N	1.959355	2.687937	1.013564
N	3.224314	1.244108	1.934287
C	-0.871166	2.735020	0.028965
H	-0.441101	1.868611	0.528156
C	3.779820	0.500384	3.053227
H	3.712449	1.135013	3.934475
H	3.207273	-0.414508	3.204499
H	4.822747	0.266747	2.845214
F	-0.182603	3.067537	-1.071941
F	-0.913004	3.805959	0.832777
C	4.376755	0.137583	-0.003899
C	4.407413	-1.193058	0.425104
C	5.192016	0.537663	-1.068041
C	5.256681	-2.104422	-0.193966
H	3.740549	-1.515786	1.215735
C	6.033462	-0.379111	-1.687928
H	5.170063	1.570304	-1.398895
C	6.071654	-1.700502	-1.248678
H	5.273676	-3.135013	0.141807
H	6.663377	-0.059832	-2.510381
H	6.732111	-2.413647	-1.728765
S	0.131344	-1.277079	1.015397
O	-0.893638	-0.221470	1.028142
O	1.388277	-0.914349	1.667741
O	-0.361054	-2.622505	1.297250
C	0.583838	-1.351705	-0.780530
F	0.886838	-0.137121	-1.243499
F	1.634228	-2.147348	-0.978450

F	-0.433198	-1.821768	-1.507357
C	-3.125201	2.102904	0.807598
C	-2.335706	1.373719	-1.442947
C	-3.800355	1.150640	-1.805953
C	-4.549793	1.831347	0.341422
H	-1.757479	1.712824	-2.300169
H	-2.669216	1.218878	1.257241
H	-3.089523	2.946060	1.495145
H	-4.216053	2.084308	-2.196064
H	-3.867094	0.412933	-2.602399
H	-4.965725	2.735503	-0.112125
H	-5.152018	1.599771	1.215084
H	-1.881684	0.488719	-0.994233
N	-2.270691	2.455395	-0.389082
H	-2.640163	3.326883	-0.802760
N	-4.598652	0.762026	-0.652259
C	-4.529674	-0.574384	-0.202274
C	-3.832367	-1.566212	-0.910921
C	-5.221150	-0.962956	0.958659
C	-3.836554	-2.888566	-0.475069
H	-3.271408	-1.326067	-1.803765
C	-5.208267	-2.283145	1.388487
H	-5.794928	-0.239489	1.523042
C	-4.516749	-3.261879	0.677704
H	-3.285410	-3.628696	-1.045394
H	-5.755485	-2.547626	2.286756
H	-4.509299	-4.290832	1.016477

$(PZ\text{-}2)_{Pre}$

C	-2.231338	-1.023482	1.589845
C	-1.121471	-0.992205	0.780554
N	-2.585933	-2.389668	-0.119039
H	-2.479281	-0.486933	2.490563

N	-3.072653	-1.886567	0.982572
N	-1.406419	-1.861613	-0.231145
C	-4.446207	-2.232881	1.351364
H	-5.123331	-2.040647	0.523989
C	-0.609578	-2.169510	-1.418404
H	-0.865093	-3.180107	-1.728792
H	-0.871805	-1.453021	-2.196982
H	0.449315	-2.098304	-1.167594
F	-4.752796	-1.504790	2.424947
F	-4.466746	-3.529702	1.698459
C	0.068487	-0.139867	0.876764
C	0.502218	0.587566	-0.235098
C	0.695580	0.021078	2.114811
C	1.569115	1.469178	-0.103339
H	-0.015887	0.492730	-1.182903
C	1.760621	0.906233	2.236980
H	0.348522	-0.545987	2.971823
C	2.197151	1.629832	1.129850
H	1.904473	2.039735	-0.962273
H	2.251436	1.030393	3.195618
H	3.029044	2.318969	1.225918
S	-3.851881	1.054146	-0.877128
O	-4.654917	0.363099	0.138722
O	-2.716139	0.277328	-1.382825
O	-4.611996	1.786418	-1.885567
C	-3.028162	2.403292	0.091761
F	-2.342112	1.910087	1.124776
F	-2.177798	3.083235	-0.678974
F	-3.929166	3.262863	0.569484
C	3.217136	-1.974345	0.076073
C	3.587362	-1.060451	-2.137979
C	5.094196	-1.111767	-1.877551
C	4.729066	-2.016526	0.310596

H	3.380117	-1.231678	-3.196917
H	2.827337	-1.029027	0.470486
H	2.728344	-2.792051	0.612704
H	5.468265	-2.098904	-2.169787
H	5.638034	-0.374088	-2.466914
H	5.130586	-2.971607	-0.043901
H	4.957857	-1.939904	1.371282
H	3.202318	-0.069661	-1.873878
N	5.392115	-0.950798	-0.452807
C	5.440295	0.337641	0.091011
C	5.339196	1.496837	-0.703821
C	5.661828	0.524029	1.471960
C	5.483004	2.764891	-0.146710
H	5.140413	1.425978	-1.764468
C	5.794623	1.794577	2.014972
H	5.760755	-0.330213	2.128387
C	5.711881	2.933329	1.214444
H	5.401564	3.631072	-0.795071
H	5.974464	1.891481	3.080578
H	5.817068	3.922767	1.643445
H	3.105931	-2.982312	-1.685347
N	2.856503	-2.055627	-1.343908

$(PZ\text{-}2)_{TS}$

C	-2.798496	-0.699217	1.882881
C	-1.551820	-0.332130	1.437428
N	-2.015455	-2.269199	0.523869
H	-3.516261	-0.218583	2.528493
N	-3.024995	-1.894805	1.291552
N	-1.134825	-1.338975	0.613863
C	-4.199160	-2.725301	1.352900
H	-4.158528	-3.480178	0.570855
C	0.450717	-1.577423	-0.354556

H	0.340236	-2.652875	-0.333318
H	0.064192	-1.012385	-1.190247
H	1.049166	-1.092048	0.403306
F	-5.284901	-1.945818	1.229587
F	-4.269451	-3.303120	2.571119
C	-0.760188	0.867759	1.744362
C	-0.015976	1.507838	0.749016
C	-0.776218	1.386225	3.041330
C	0.718180	2.647622	1.058649
H	-0.049641	1.133199	-0.267541
C	-0.046719	2.531429	3.342532
H	-1.349363	0.884778	3.813878
C	0.705625	3.160471	2.353953
H	1.290196	3.143862	0.282742
H	-0.060560	2.926995	4.351514
H	1.277043	4.050514	2.591923
S	-3.016153	0.391295	-1.991985
O	-3.688280	-0.564593	-1.110894
O	-1.551521	0.385804	-1.912901
O	-3.561852	0.488820	-3.344999
C	-3.465331	2.023922	-1.239840
F	-3.085077	2.089562	0.036695
F	-2.880747	3.029417	-1.895254
F	-4.785858	2.217988	-1.284975
C	3.191390	-2.460349	-0.520855
C	2.633228	-0.469383	-1.815727
C	4.038761	-0.509058	-2.415243
C	4.586759	-2.459903	-1.145152
H	1.911013	-0.085770	-2.539903
H	3.215831	-1.910159	0.427243
H	2.857077	-3.480268	-0.319071
H	4.026952	-1.147605	-3.303936
H	4.364412	0.479464	-2.735567

H	4.574379	-3.038853	-2.073247
H	5.297399	-2.934010	-0.472784
H	2.612309	0.179016	-0.932728
N	5.001701	-1.092401	-1.480395
C	5.514314	-0.283340	-0.452683
C	5.234522	1.092873	-0.367408
C	6.391823	-0.825047	0.508088
C	5.809529	1.881003	0.626154
H	4.558804	1.566115	-1.066502
C	6.950079	-0.029708	1.499190
H	6.664134	-1.871732	0.470884
C	6.666608	1.332968	1.573177
H	5.568264	2.938260	0.657915
H	7.625221	-0.483455	2.216917
H	7.105277	1.949879	2.348183
H	2.019052	-2.386984	-2.202342
N	2.199644	-1.805766	-1.384276

$(PZ\text{-}2)_{Post}$

C	3.679199	1.441424	-0.738638
C	2.315799	1.578372	-0.825102
N	2.876992	0.164464	-2.372084
H	4.424278	1.856872	-0.079570
N	3.975883	0.557059	-1.717754
N	1.877377	0.776011	-1.843476
C	5.241836	0.003438	-2.083444
H	5.100734	-0.887176	-2.690506
C	-1.064715	-0.836164	-2.146301
H	-0.937897	-1.262471	-3.140348
H	-0.149917	-0.925132	-1.563188
H	-1.376209	0.205689	-2.217860
F	5.944305	-0.270515	-0.968618
F	5.961791	0.925642	-2.770095

C	1.396566	2.367211	0.004413
C	0.112961	1.878518	0.263759
C	1.798229	3.580316	0.567558
C	-0.761402	2.601183	1.066976
H	-0.172216	0.914734	-0.140534
C	0.923048	4.297712	1.377441
H	2.791789	3.965759	0.363290
C	-0.359051	3.812935	1.625743
H	-1.754361	2.214037	1.268881
H	1.240403	5.239793	1.809935
H	-1.040494	4.375399	2.253823
S	2.140573	-1.930723	0.923114
O	3.295978	-1.601756	0.090397
O	0.843697	-1.485148	0.400334
O	2.139878	-3.277637	1.494405
C	2.378148	-0.853056	2.413334
F	2.432701	0.436866	2.084531
F	1.372873	-1.014074	3.278798
F	3.514219	-1.163026	3.043940
C	-3.429016	-1.548842	-2.217707
C	-2.339696	-1.097262	-0.044597
C	-3.451635	-1.905298	0.613070
C	-4.506583	-2.330714	-1.477439
H	-1.387244	-1.212608	0.472787
H	-3.696615	-0.493932	-2.313089
H	-3.231886	-1.970065	-3.203481
H	-3.160267	-2.960261	0.629137
H	-3.577785	-1.594996	1.647937
H	-4.207908	-3.379853	-1.395636
H	-5.422725	-2.298613	-2.060261
H	-2.594524	-0.038184	-0.127301
N	-4.706411	-1.811824	-0.124105
C	-5.506800	-0.664608	0.042669

C	-5.206512	0.327867	0.991264
C	-6.686690	-0.511829	-0.709354
C	-6.058272	1.413175	1.182193
H	-4.308421	0.272697	1.591699
C	-7.521711	0.580045	-0.515921
H	-6.973691	-1.262476	-1.434226
C	-7.219292	1.556026	0.431961
H	-5.795964	2.157990	1.925923
H	-8.425684	0.660691	-1.109905
H	-7.874980	2.405336	0.582072
H	-1.848519	-2.571750	-1.391885
N	-2.142896	-1.589083	-1.449710

9. Natural population analysis

$(PZ\text{-}1)_{Pre}$

Natural Population

Atom	No	Natural Charge	Core	Valence	Rydberg	Total
C	1	0.02917	1.99907	3.95333	0.01843	5.97083
C	2	0.18862	1.99907	3.78947	0.02284	5.81138
N	3	0.01969	1.99948	4.95032	0.03051	6.98031
H	4	0.29446	0.00000	0.70397	0.00157	0.70554
N	5	-0.20452	1.99932	5.17631	0.02888	7.20452
N	6	-0.10480	1.99928	5.08190	0.02362	7.10480
C	7	0.78972	1.99924	3.16638	0.04466	5.21028
H	8	0.23810	0.00000	0.75919	0.00271	0.76190
C	9	-0.37696	1.99934	4.36464	0.01297	6.37696
H	10	0.24854	0.00000	0.75039	0.00107	0.75146
H	11	0.24536	0.00000	0.75343	0.00121	0.75464
H	12	0.24472	0.00000	0.75426	0.00102	0.75528
F	13	-0.35058	1.99992	7.34118	0.00948	9.35058
F	14	-0.36160	1.99993	7.35512	0.00656	9.36160
C	15	-0.11849	1.99903	4.10334	0.01612	6.11849
C	16	-0.18820	1.99912	4.17333	0.01575	6.18820
C	17	-0.18186	1.99913	4.16689	0.01584	6.18186
C	18	-0.21269	1.99921	4.19573	0.01775	6.21269
H	19	0.24330	0.00000	0.75522	0.00148	0.75670
C	20	-0.21438	1.99921	4.19749	0.01768	6.21438
H	21	0.24093	0.00000	0.75790	0.00116	0.75907
C	22	-0.20090	1.99922	4.18410	0.01758	6.20090
H	23	0.23620	0.00000	0.76268	0.00112	0.76380
H	24	0.23591	0.00000	0.76297	0.00112	0.76409
H	25	0.23398	0.00000	0.76499	0.00103	0.76602
S	26	2.28814	9.99895	3.46566	0.24725	13.71186

O	27	-1.01756	1.99981	6.99981	0.01794	9.01756
O	28	-1.01904	1.99981	7.00207	0.01716	9.01904
O	29	-0.98883	1.99981	6.97490	0.01412	8.98883
C	30	0.84391	1.99980	3.07775	0.07853	5.15609
F	31	-0.36203	1.99991	7.35111	0.01101	9.36203
F	32	-0.36143	1.99991	7.35182	0.00970	9.36143
F	33	-0.35932	1.99991	7.34815	0.01127	9.35932
C	34	-0.21798	1.99933	4.20076	0.01790	6.21798
C	35	-0.22046	1.99932	4.20241	0.01873	6.22046
C	36	-0.20221	1.99930	4.18415	0.01875	6.20221
C	37	-0.19406	1.99929	4.17697	0.01780	6.19406
H	38	0.20883	0.00000	0.78986	0.00131	0.79117
H	39	0.20531	0.00000	0.79273	0.00196	0.79469
H	40	0.20883	0.00000	0.78988	0.00129	0.79117
H	41	0.20649	0.00000	0.79170	0.00181	0.79351
H	42	0.21768	0.00000	0.78084	0.00148	0.78232
H	43	0.20382	0.00000	0.79428	0.00190	0.79618
H	44	0.21794	0.00000	0.78056	0.00149	0.78206
H	45	0.20798	0.00000	0.78972	0.00230	0.79202
N	46	-0.72199	1.99958	5.69860	0.02381	7.72199
H	47	0.36750	0.00000	0.63009	0.00241	0.63250
N	48	-0.54349	1.99940	5.51857	0.02552	7.54349
C	49	0.18641	1.99903	3.78968	0.02488	5.81359
C	50	-0.29133	1.99913	4.27608	0.01612	6.29133
C	51	-0.27848	1.99914	4.26329	0.01606	6.27848
C	52	-0.20146	1.99922	4.18349	0.01874	6.20146
H	53	0.22963	0.00000	0.76874	0.00163	0.77037
C	54	-0.20694	1.99922	4.18972	0.01799	6.20694
H	55	0.22800	0.00000	0.77062	0.00138	0.77200
C	56	-0.28166	1.99920	4.26278	0.01968	6.28166
H	57	0.22429	0.00000	0.77446	0.00125	0.77571
H	58	0.22471	0.00000	0.77417	0.00113	0.77529
H	59	0.22509	0.00000	0.77373	0.00117	0.77491

* Total *		0.00000	77.97865	191.04368	0.97766	270.00000

$(PZ\text{-}1)_{TS}$

Natural Population

Atom	No	Natural Charge	Core	Valence	Rydberg	Total
C	1	-0.03128	1.99908	4.01225	0.01995	6.03128
C	2	0.16517	1.99906	3.81300	0.02277	5.83483
N	3	-0.05466	1.99950	5.02358	0.03159	7.05466
H	4	0.26453	0.00000	0.73407	0.00140	0.73547
N	5	-0.26550	1.99940	5.22606	0.04004	7.26550
N	6	-0.14688	1.99927	5.12244	0.02516	7.14688
C	7	0.81970	1.99924	3.12790	0.05316	5.18030
H	8	0.25415	0.00000	0.74294	0.00291	0.74585
C	9	-0.37747	1.99935	4.36519	0.01292	6.37747
H	10	0.23956	0.00000	0.75933	0.00112	0.76044
H	11	0.23578	0.00000	0.76295	0.00127	0.76422
H	12	0.23572	0.00000	0.76320	0.00108	0.76428
F	13	-0.34537	1.99990	7.33621	0.00927	9.34537
F	14	-0.34050	1.99989	7.33221	0.00840	9.34050
C	15	-0.10709	1.99904	4.09196	0.01609	6.10709
C	16	-0.19515	1.99912	4.17998	0.01604	6.19515
C	17	-0.18927	1.99913	4.17421	0.01593	6.18927
C	18	-0.21514	1.99921	4.19811	0.01781	6.21514
H	19	0.24213	0.00000	0.75638	0.00149	0.75787
C	20	-0.21681	1.99921	4.19985	0.01775	6.21681
H	21	0.23767	0.00000	0.76112	0.00121	0.76233
C	22	-0.20960	1.99922	4.19265	0.01774	6.20960
H	23	0.23387	0.00000	0.76500	0.00113	0.76613
H	24	0.23361	0.00000	0.76526	0.00113	0.76639

H	25	0.23211	0.00000	0.76684	0.00105	0.76789
S	26	2.28654	9.99894	3.46470	0.24983	13.71346
O	27	-1.02273	1.99980	7.00550	0.01743	9.02273
O	28	-1.00497	1.99981	6.98778	0.01739	9.00497
O	29	-0.98589	1.99981	6.97488	0.01120	8.98589
C	30	0.84442	1.99980	3.07795	0.07783	5.15558
F	31	-0.36540	1.99991	7.35329	0.01220	9.36540
F	32	-0.35805	1.99991	7.34862	0.00952	9.35805
F	33	-0.35807	1.99991	7.34841	0.00974	9.35807
C	34	-0.21872	1.99931	4.20308	0.01634	6.21872
C	35	-0.22284	1.99930	4.20707	0.01646	6.22284
C	36	-0.19872	1.99930	4.18042	0.01900	6.19872
C	37	-0.19220	1.99928	4.17468	0.01824	6.19220
H	38	0.22973	0.00000	0.76894	0.00133	0.77027
H	39	0.21874	0.00000	0.77962	0.00164	0.78126
H	40	0.22901	0.00000	0.76970	0.00129	0.77099
H	41	0.21719	0.00000	0.78140	0.00142	0.78281
H	42	0.22710	0.00000	0.77164	0.00126	0.77290
H	43	0.21436	0.00000	0.78419	0.00145	0.78564
H	44	0.22674	0.00000	0.77200	0.00125	0.77326
H	45	0.21440	0.00000	0.78270	0.00290	0.78560
N	46	-0.62952	1.99954	5.60457	0.02541	7.62952
H	47	0.40922	0.00000	0.58935	0.00143	0.59078
N	48	-0.54035	1.99941	5.51710	0.02385	7.54035
C	49	0.18271	1.99903	3.79479	0.02347	5.81729
C	50	-0.28603	1.99913	4.27139	0.01552	6.28603
C	51	-0.27500	1.99913	4.26019	0.01568	6.27500
C	52	-0.20252	1.99922	4.18457	0.01873	6.20252
H	53	0.23049	0.00000	0.76803	0.00148	0.76951
C	54	-0.20425	1.99922	4.18733	0.01770	6.20425
H	55	0.22983	0.00000	0.76884	0.00132	0.77017
C	56	-0.27395	1.99920	4.25545	0.01930	6.27395
H	57	0.22636	0.00000	0.77244	0.00119	0.77364

H	58	0.22622	0.00000	0.77267	0.00111	0.77378
H	59	0.22687	0.00000	0.77198	0.00114	0.77313
* Total *		0.00000	77.97858	191.02796	0.99345	270.00000

$(PZ\text{-}2)_{Pre}$

Natural Population

Atom	No	Natural Charge	Core	Valence	Rydberg	Total
C	1	0.03222	1.99906	3.94993	0.01879	5.96778
C	2	0.18447	1.99905	3.78956	0.02691	5.81553
N	3	0.00653	1.99948	4.96480	0.02919	6.99347
H	4	0.29604	0.00000	0.70234	0.00163	0.70396
N	5	-0.20206	1.99931	5.17440	0.02834	7.20206
N	6	-0.10177	1.99928	5.07806	0.02442	7.10177
C	7	0.80229	1.99932	3.15400	0.04439	5.19771
H	8	0.22208	0.00000	0.77630	0.00162	0.77792
C	9	-0.39378	1.99932	4.38053	0.01393	6.39378
H	10	0.24587	0.00000	0.75306	0.00107	0.75413
H	11	0.24394	0.00000	0.75499	0.00107	0.75606
H	12	0.26009	0.00000	0.73796	0.00196	0.73991
F	13	-0.34522	1.99992	7.33804	0.00725	9.34522
F	14	-0.35422	1.99993	7.34882	0.00547	9.35422
C	15	-0.12103	1.99902	4.09948	0.02254	6.12103
C	16	-0.19578	1.99911	4.17340	0.02327	6.19578
C	17	-0.18280	1.99913	4.16849	0.01518	6.18280
C	18	-0.21362	1.99919	4.19753	0.01689	6.21362
H	19	0.24283	0.00000	0.75521	0.00195	0.75717
C	20	-0.21445	1.99920	4.19843	0.01683	6.21445
H	21	0.24068	0.00000	0.75815	0.00116	0.75932
C	22	-0.20294	1.99920	4.18596	0.01778	6.20294

H	23	0.23723	0.00000	0.76162	0.00115	0.76277
H	24	0.23606	0.00000	0.76278	0.00116	0.76394
H	25	0.23675	0.00000	0.76081	0.00243	0.76325
S	26	2.28094	9.99896	3.46903	0.25107	13.71906
O	27	-1.01901	1.99981	7.00221	0.01699	9.01901
O	28	-1.01692	1.99981	6.99998	0.01713	9.01692
O	29	-0.98828	1.99981	6.97757	0.01090	8.98828
C	30	0.84678	1.99980	3.07545	0.07797	5.15322
F	31	-0.36073	1.99991	7.34998	0.01083	9.36073
F	32	-0.35939	1.99991	7.34999	0.00949	9.35939
F	33	-0.36003	1.99991	7.35084	0.00928	9.36003
C	34	-0.21850	1.99931	4.20199	0.01720	6.21850
C	35	-0.21286	1.99932	4.19642	0.01712	6.21286
C	36	-0.20180	1.99930	4.18444	0.01807	6.20180
C	37	-0.19239	1.99929	4.17605	0.01706	6.19239
H	38	0.21048	0.00000	0.78831	0.00121	0.78952
H	39	0.20025	0.00000	0.79634	0.00342	0.79975
H	40	0.20982	0.00000	0.78895	0.00123	0.79018
H	41	0.20823	0.00000	0.79028	0.00149	0.79177
H	42	0.21868	0.00000	0.77983	0.00149	0.78132
H	43	0.20553	0.00000	0.79288	0.00158	0.79447
H	44	0.21831	0.00000	0.78027	0.00141	0.78169
H	45	0.19974	0.00000	0.79850	0.00176	0.80026
N	46	-0.54296	1.99940	5.51782	0.02574	7.54296
C	47	0.18815	1.99903	3.78725	0.02557	5.81185
C	48	-0.29217	1.99913	4.27747	0.01557	6.29217
C	49	-0.27682	1.99913	4.26185	0.01584	6.27682
C	50	-0.20475	1.99922	4.18758	0.01796	6.20475
H	51	0.23131	0.00000	0.76730	0.00139	0.76869
C	52	-0.20706	1.99922	4.18987	0.01798	6.20706
H	53	0.22961	0.00000	0.76911	0.00128	0.77039
C	54	-0.28301	1.99920	4.26457	0.01925	6.28301
H	55	0.22642	0.00000	0.77246	0.00111	0.77358

H	56	0.22634	0.00000	0.77255	0.00111	0.77366
H	57	0.22711	0.00000	0.77173	0.00115	0.77289
H	58	0.36808	0.00000	0.62975	0.00217	0.63192
N	59	-0.71849	1.99957	5.69320	0.02571	7.71849
* Total *		0.00000	77.97858	191.03649	0.98493	270.00000

$(PZ\text{-}2)_{TS}$

Natural Population

Atom	No	Natural Charge	Core	Valence	Rydberg	Total
C	1	0.01810	1.99907	3.96531	0.01751	5.98190
C	2	0.12600	1.99907	3.84725	0.02768	5.87400
N	3	-0.01998	1.99950	4.98969	0.03079	7.01998
H	4	0.28244	0.00000	0.71608	0.00148	0.71756
N	5	-0.23205	1.99932	5.20692	0.02582	7.23205
N	6	-0.18272	1.99935	5.14577	0.03760	7.18272
C	7	0.79045	1.99928	3.16699	0.04328	5.20955
H	8	0.21328	0.00000	0.78517	0.00156	0.78672
C	9	-0.29165	1.99947	4.27947	0.01271	6.29165
H	10	0.22603	0.00000	0.77261	0.00136	0.77397
H	11	0.24304	0.00000	0.75530	0.00166	0.75696
H	12	0.22291	0.00000	0.77574	0.00135	0.77709
F	13	-0.35479	1.99993	7.34798	0.00688	9.35479
F	14	-0.36341	1.99993	7.35822	0.00526	9.36341
C	15	-0.10157	1.99903	4.08297	0.01958	6.10157
C	16	-0.20136	1.99912	4.18597	0.01627	6.20136
C	17	-0.19248	1.99914	4.17838	0.01496	6.19248
C	18	-0.21608	1.99921	4.19936	0.01752	6.21608
H	19	0.24645	0.00000	0.75108	0.00247	0.75355
C	20	-0.21653	1.99921	4.20009	0.01723	6.21653

H	21	0.23603	0.00000	0.76279	0.00118	0.76397
C	22	-0.21104	1.99921	4.19431	0.01751	6.21104
H	23	0.23229	0.00000	0.76656	0.00115	0.76771
H	24	0.23311	0.00000	0.76582	0.00106	0.76689
H	25	0.23148	0.00000	0.76744	0.00108	0.76852
S	26	2.28314	9.99896	3.46679	0.25111	13.71686
O	27	-1.00894	1.99981	6.99166	0.01747	9.00894
O	28	-1.01596	1.99980	6.99868	0.01747	9.01596
O	29	-0.99600	1.99982	6.98531	0.01087	8.99600
C	30	0.84467	1.99980	3.07754	0.07799	5.15533
F	31	-0.35998	1.99991	7.34873	0.01134	9.35998
F	32	-0.36266	1.99991	7.35310	0.00964	9.36266
F	33	-0.36317	1.99991	7.35356	0.00969	9.36317
C	34	-0.20532	1.99931	4.19030	0.01570	6.20532
C	35	-0.21083	1.99931	4.19540	0.01612	6.21083
C	36	-0.20015	1.99930	4.18207	0.01878	6.20015
C	37	-0.19211	1.99929	4.17506	0.01776	6.19211
H	38	0.22563	0.00000	0.77292	0.00145	0.77437
H	39	0.20880	0.00000	0.78969	0.00151	0.79120
H	40	0.22447	0.00000	0.77426	0.00127	0.77553
H	41	0.21516	0.00000	0.78329	0.00155	0.78484
H	42	0.22548	0.00000	0.77319	0.00134	0.77452
H	43	0.21176	0.00000	0.78663	0.00161	0.78824
H	44	0.22476	0.00000	0.77388	0.00136	0.77524
H	45	0.20839	0.00000	0.78898	0.00262	0.79161
N	46	-0.54063	1.99940	5.51801	0.02322	7.54063
C	47	0.18644	1.99903	3.79411	0.02042	5.81356
C	48	-0.28957	1.99913	4.27470	0.01574	6.28957
C	49	-0.27408	1.99913	4.25854	0.01640	6.27408
C	50	-0.20365	1.99922	4.18627	0.01815	6.20365
H	51	0.23123	0.00000	0.76736	0.00141	0.76877
C	52	-0.20614	1.99922	4.18904	0.01788	6.20614
H	53	0.23017	0.00000	0.76850	0.00133	0.76983

C	54	-0.27594	1.99920	4.25743	0.01931	6.27594
H	55	0.22642	0.00000	0.77248	0.00110	0.77358
H	56	0.22656	0.00000	0.77236	0.00108	0.77344
H	57	0.22681	0.00000	0.77205	0.00114	0.77319
H	58	0.39730	0.00000	0.59942	0.00329	0.60270
N	59	-0.61002	1.99954	5.58831	0.02217	7.61002
* Total *		0.00000	77.97887	191.04690	0.97423	270.00000
